# Lighting up solid states using a rubber

**DOI:** 10.1038/s41467-021-21253-w

**Published:** 2021-02-10

**Authors:** Zhongyu Li, Yanjie Wang, Gleb Baryshnikov, Shen Shen, Man Zhang, Qi Zou, Hans Ågren, Liangliang Zhu

**Affiliations:** 1 State Key Laboratory of Molecular Engineering of Polymers, Department of Macromolecular Science, Fudan University, Shanghai, China; 2 Division of Theoretical Chemistry and Biology School of Biotechnology, KTH Royal Institute of Technology, Stockholm, Sweden; 3 Tomsk State University, Tomsk, Russia; 4 Shanghai Key Laboratory of Materials Protection and Advanced Materials in Electric Power, Shanghai University of Electric Power, Shanghai, China; 5 Department of Physics and Astronomy, Uppsala University, Uppsala, Sweden

**Keywords:** Polymers, Organic molecules in materials science, Polymers

## Abstract

It is crucial and desirable to develop green and high-efficient strategies to regulate solid-state structures and their related material properties. However, relative to solution, it is more difficult to break and generate chemical bonds in solid states. In this work, a rubbing-induced photoluminescence on the solid states of ortho-pyridinil phenol family was achieved. This rubbing response relied on an accurately designed topochemical tautomerism, where a negative charge, exactly provided by the triboelectric effect of a rubber, can induce a proton transfer in a double H-bonded dimeric structure. This process instantaneously led to a bright-form tautomer that can be stabilized in the solid-state settings, leading to an up to over 450-fold increase of the fluorescent quantum yield of the materials. The property can be repeatedly used due to the reversibility of the tautomerism, enabling encrypted applications. Moreover, a further modification to the structure can be accomplished to achieve different properties, opening up more possibilities for the design of new-generation smart materials.

## Abstract (web summary)

Changes in molecular properties due to stimuli response are critically important for the development of new materials. However, most processes are slow or inefficient in the solid state. Here the authors demonstrate property switching in the solid state using a rubbing-induced tautomerism in multiple hydrogen-bonded donor-acceptor couples.

## Introduction

Changes in molecular properties owing to stimuli response are critically important for the development of new materials^1–9^. Thus far, numerous materials have been reported as being sensitive to a variety of physical factors, such as light, heat, magnetic fields, pressure, and mechanical forces^10–21^. However, most of these stimuli-responsive processes tend to be slow and incomplete, fostering generally mild changes in material properties. By contrast, chemical reaction-driven stimuli responses, such as pH change, redox reactions, and coordination effects, are usually rapid and sufficiently extensive because of direct contact between the interacting species^22–29^. Nonetheless, this type of stimuli-responsive behavior is generally only effective in solutions. When performed in the solid-state, physical and chemical stimuli approaches tend to be less efficient^30,31^, thus restricting their material applications.

In recent decades, various solid-state reaction methods have been developed, most of which rely on the principle of topochemical reactions for creating new substances and materials^32–34^. The preorganization of the reactants for minimal atom displacement during the breaking and making of bonds is key to topochemistry, and so highly ordered circumstances or systems with complex technical efforts are necessary^35^. On the other hand, in solid-state reactions, the accumulation of byproducts and the irreversibility of reactions are also aggravating effects that prohibit their further use for the design of materials with certain properties^36,37^. The development of green and highly efficient strategies to regulate solid-state structures and related material properties, therefore, remains an urgent challenge to be overcome.

This prompts us to consider an integration of tautomerization and topochemistry, which may provide a unique solution to reaction efficiency and reversibility issues. Multiple hydrogen-bonded structures have been widely employed for the design and development of new supramolecular, polymeric, and nanomaterials^38–43^. Because of the relatively high binding constant, multiple hydrogen-bonded structures can also be maintained in the solid state^44,45^, which can therefore play a role in topochemical reactions^46^. In addition, energy and information transfer (e.g., chirality, configurational information, DNA sequences, etc.) can be easily conducted in such hydrogen-bonded structures^47–49^. Drawing inspiration from these advantageous properties, herein, we propose that a tautomerism in multiple hydrogen-bonded donor-receptor couples can be favorable for material property switches driven by green and mild stimuli, such as triboelectric effects upon rubbing. The H-bond donor–receptor couple proposed here is assumed to be enrolled in a π-skeleton push-pull system to regulate its optoelectronic properties upon the tautomerism process.

**Fig. 1:**
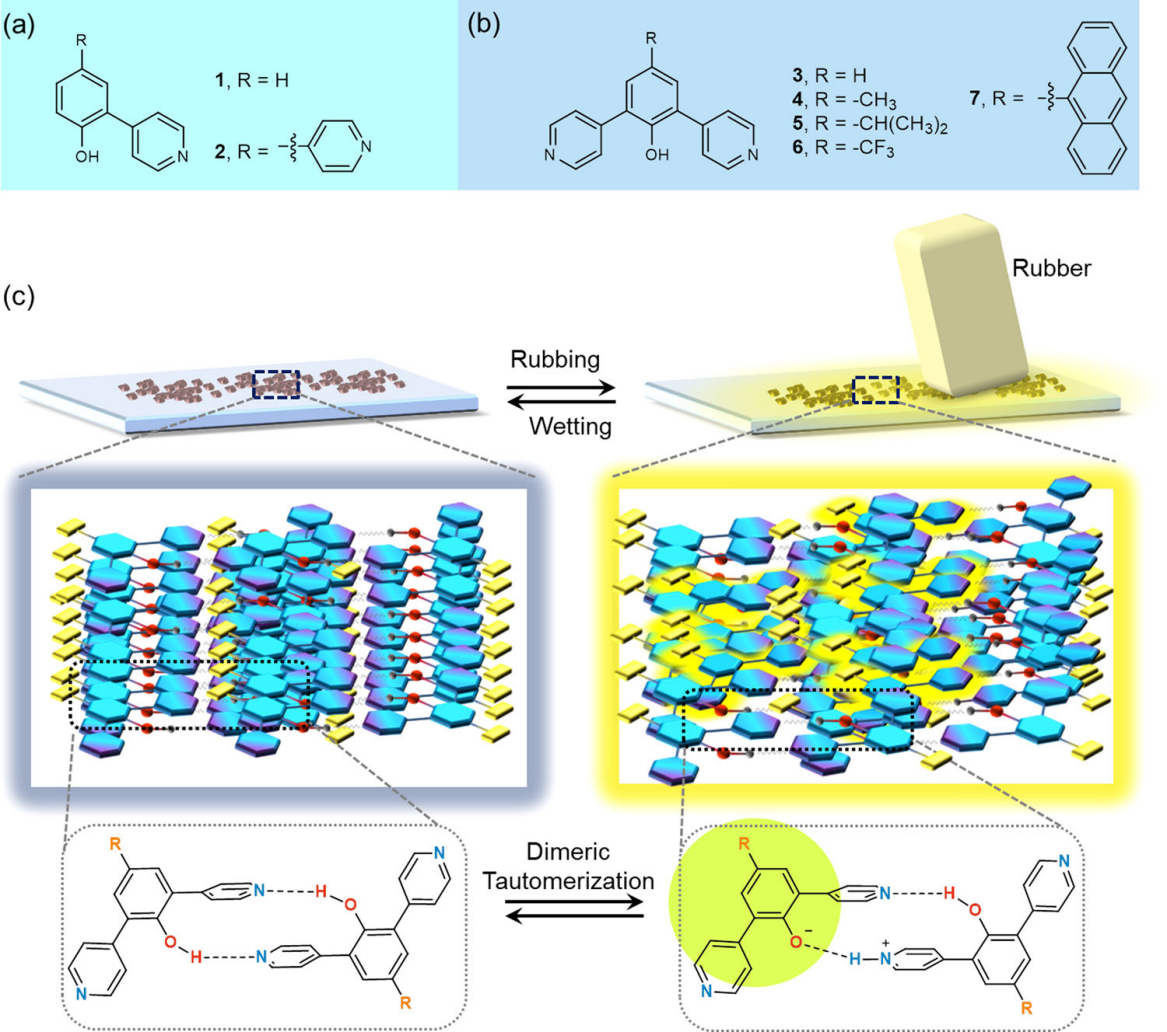
Research outline. Chemical structure of (**a**) mono-ortho-pyridinil phenols 1 and 2, and (**b**) bis-ortho-pyridinil phenols 3~7. **c** Illustration of rubbing-induced photoluminescence on the solid sample of bis-ortho-pyridinil phenols in conjunction with a topochemical tautomerization, relying on a negative charge (exactly provided by the triboelectric effect of a rubber)-induced proton transfer process in the double H-bonded dimeric tautomers. The material was simply spread on a frosted glass surface and then was directly abraded with a rubber.

In this work, we employ ortho-pyridinil phenol-based compounds as prototypes for our studies (compound 1 and 2; see the chemical structure in Fig. 1a). At the same time, we also designed and synthesized a series of reference compounds for control studies in order to explore the role of substituent positions and end groups. In our structural design, a double H-bonded dimer is formed by the intermolecular hydrogen bonds between the phenolic hydroxyl and the pyridinyl groups of the ortho-pyridinil phenol structure (Fig. 1b). Eventually, we identified a rubbing-induced photoluminescence mechanism based on a negative charge (exactly provided by the triboelectric effect of a rubber)-induced proton transfer in the H-bonded dimers, which leads to topochemical tautomerism and that can be stabilized in the solid-state (as illustrated in Fig. 1b). The superior behavior of photoluminescence enhancement in this system can be employed to visualize and quantify the stimuli-responsive process in its entirety.

## Results

A series of mono- and bis-ortho-pyridinil phenols were synthesized by means of Suzuki–Miyaura cross-coupling reactions (Schemes S1–S9). The structures of these compounds were confirmed by ^1^H NMR, ^13^C NMR, and mass spectrometry (Figures S15–S71). All of these compounds were shown to exhibit a rubbing-induced photoluminescence property owing to the skeleton design of the ortho-pyridinil phenols. Bis-ortho-pyridinil phenols were found to possess a higher quantum yield of the rubbing-induced photoluminescence than the mono-ortho-pyridinil phenols (see a comparison of the luminescent quantum yields in Table S1). Thus, we focused on bis-ortho-pyridinil phenols for further investigation.

**Fig. 2:**
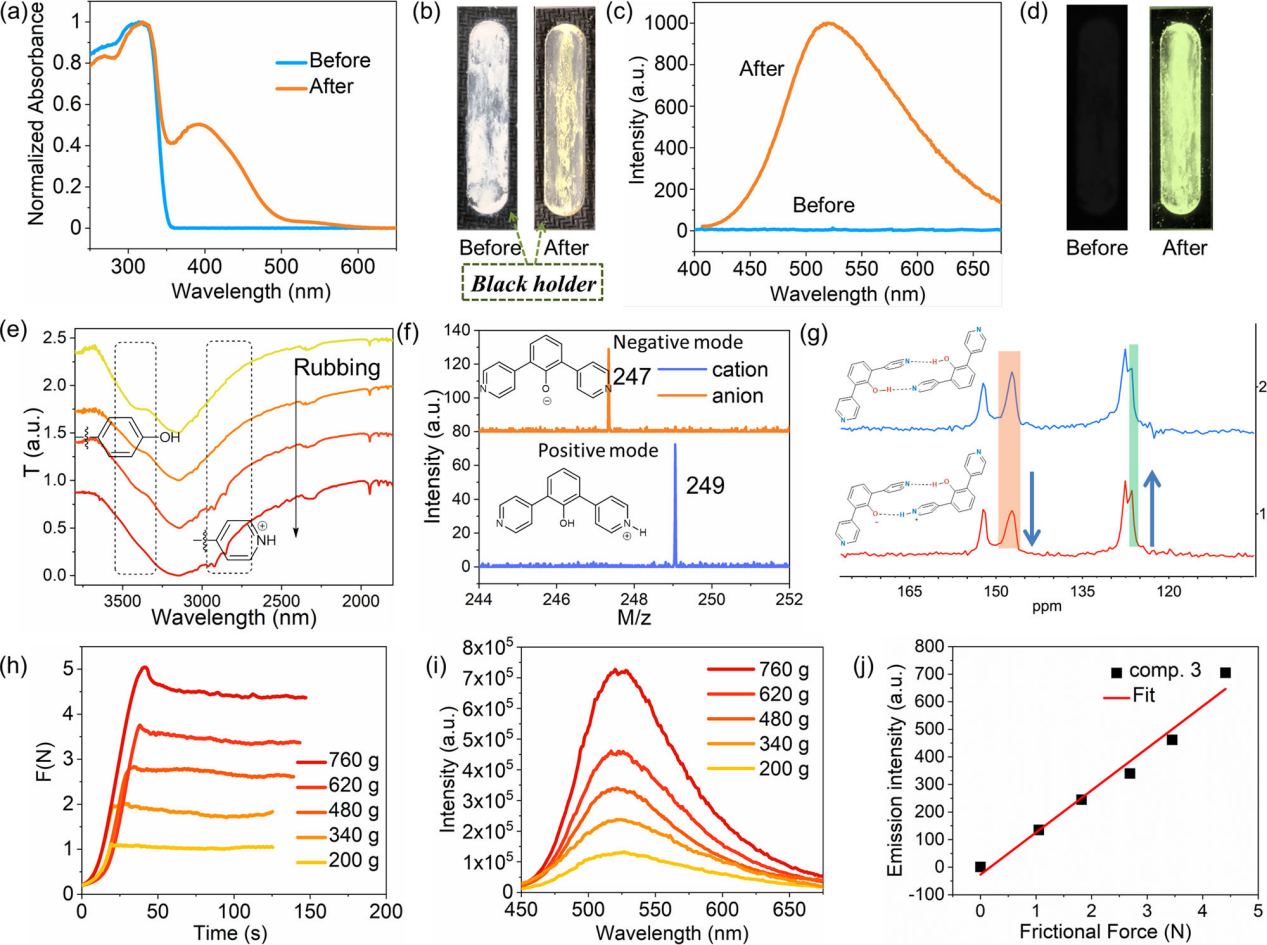
Rubbing luminescence. **a** Absorption spectra and (**b**) the corresponding photograph under daylight of 3 in the solid state before and after rubbing; **c** emission spectra and (**d**) the corresponding photograph under UV light of 3 in the solid state before and after rubbing. **e** FTIR spectra of 3 upon a continuous rubbing process. **f** MALDI-TOF results of 3 after rubbing; **g** partially magnified solid state ^13^C NMR spectra of compound 3 before (blue) and after rubbing (red). **h** Friction diagram between the rubber and glass substrate of the solid sample of 3 under different loads. **i** Friction force-dependent emission spectra of 3. **j** Plots of the emission intensity vs. the friction force.

Compound 3 is a white powder in the initial state without any emission signal. After being rubbed, it becomes a yellow solid and emits a strong green fluorescence under UV light excitation (see Supplementary Movie 1). As is shown in Fig. 2a–d, a distinct absorption band at ~400 nm and an emission band maximum at 528 nm appears after rubbing. This newly generated absorption band corresponds to the excitation, producing the emergent emission peak (see the excitation spectra in Figure S1 for this association). The nanosecond scale of the luminescent lifetime confirms the fluorescent nature dominant in the observed emission (Figure S2).

Owing to such a unique optical property of compound 3 prior to and after rubbing, we sought to investigate the possible changes in structural factors that took place during this process. In this paper, we used fourier-transform infrared spectroscopy to track the in situ solid-state structural information upon rubbing. Figure 2e demonstrates that the characteristic stretching frequency of the phenolic hydroxyl group (probably also in an H-bonding environment) initially shows up at ~3300–3500 cm^−1^. During continuous rubbing, this peak is substantially weakened and is accompanied by the emergence of the characteristic N-H absorption peak at 2800–2900 cm^−1^ corresponding to a pyridinium group. This indicates that a rubbing-induced proton transfer occurred between the hydroxyl group and the pyridinyl one. A MALDI-TOF study clearly indicates the corresponding products following the proton transfer process (Fig. 2f). Although the initial molecular weight of 3 must be obtained through matrix doping, the molecular mass of a protonated and deprotonated product after rubbing can be both be determined with the switch of the positive and negative ion modes, but without matrix doping. Moreover, the resonance of the hydroxyl group-linked carbon atom in the solid-state ^13^C NMR spectra was reduced upon rubbing, whereas the resonance of the oxygen anion-linked carbon atom increases (Figs. 2g and S3). These changes in resonance can further verify the rubbing-induced proton transfer. The rubbing-induced photoluminescence boosted along with the increase of temperature (Figure S4), indicating that temperature can also facilitate the proton transfer within the dimer.

The frictional force, which can be controlled by the mass load, is, therefore, an important factor the affects the proton transfer. As the mass of the loads increases from 200 g to 760 g, the frictional force at the interface between the rubber and glass substrate of the solid sample increased from 1.1 N to 4.5 N (see Fig. 2h). The emission intensity of compound 3 in its solid-state form was correspondingly boosted. Figure 2i shows the frictional force-dependent emission spectra of 3, with the signal reaching a very high level upon a large frictional force. The amount of charge generated by friction is proportional to the energy from friction, leading to the increase of the number of proton-transferred dimers. The linear relationship indicates that the intensity of the emission was approximately proportional to the friction force (Fig. 2j), implying that quantitative control of the rubbing luminescence can be achieved.

**Fig. 3:**
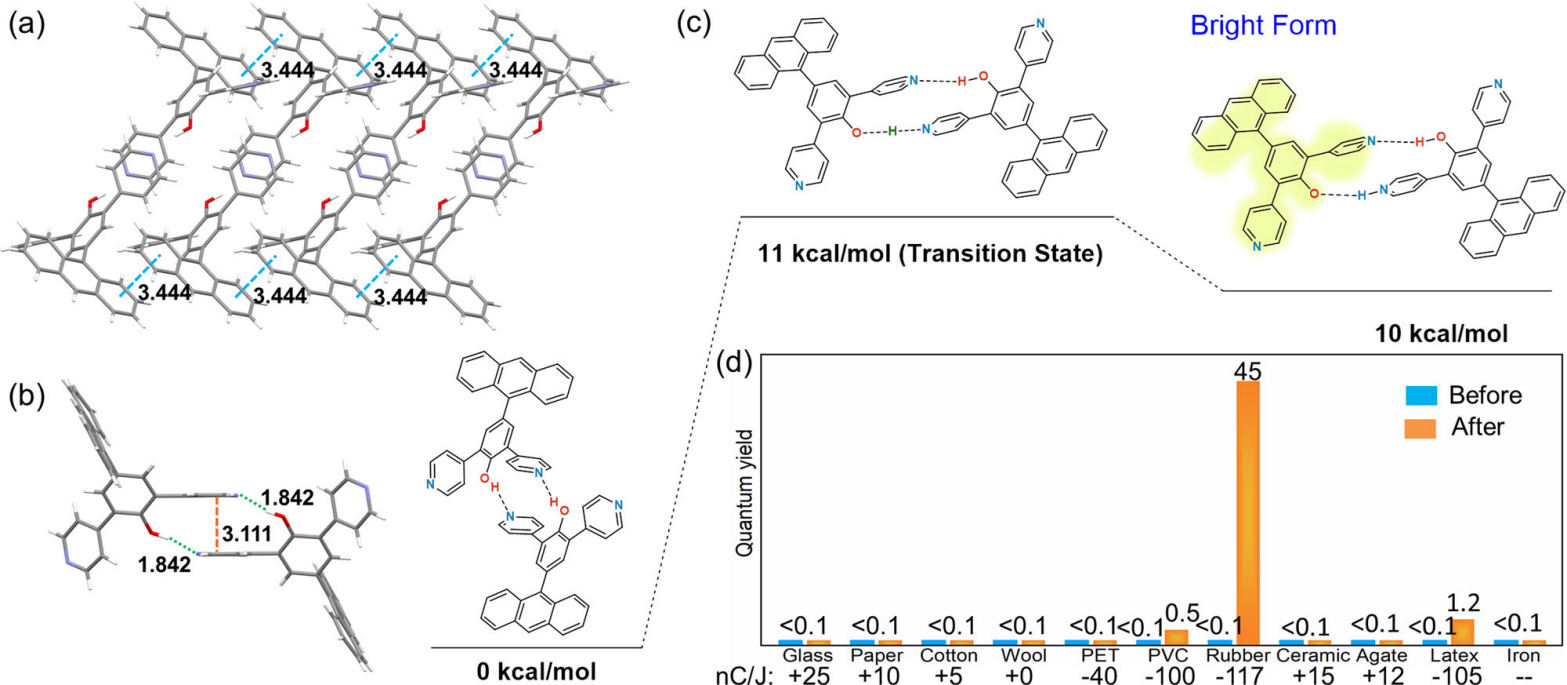
Mechanism investigation. X-ray crystallographic structure of seven in (**a**) a packing view and (**b**) a dimeric view; **c** energy calculation diagram of a proposed proton transfer process in the double H-bonded dimer of 7; **d** The quantum yield of the solid sample of 3 before and after rubbing with different materials.

To gain more evidence of intermolecular proton transfer, we attempted to obtain information about the hydrogen bonds in the solid state from the single-crystal structure. By means of solvent optimization, we eventually obtained a single crystal of 7, owing to the fact that the anthracene group at the para-position of the hydroxyl group in this compound can promote single-crystal growth (Figure S5 and Table S2). As can be from the single-crystal structural analysis in Fig. 3a and b, it is evident that two molecules of seven can indeed form a dimer via the two intermolecular hydrogen bonds. The length of each of the two hydrogen bonds is 1.842 Å (Fig. 3b), which is much shorter than typical hydrogen bonds^50–53^. Such a short distance indicates a unique topochemical probability in which a proton transfer can be more easily facilitated within the dimer. On the other hand, the rubbing process that can exert pressure will probably shorten the distance of hydrogen bonds further and increase the possibility of proton transfer. The distance between the adjacent anthracene surfaces is 3.444 Å (Fig. 3a), which is a typical π-π stacking distance. Meanwhile, the π-π stacking effect promotes the single-crystal growth. With the increase of the concentration of compound 3, there has been an obvious chemical shift for the α-H pyridine group (see the concentration-dependent ^1^H NMR in Figure S6). This indicates that the intermolecular hydrogen bonds are widespread in the compound.

At the next stage, we carried out quantum-chemical calculations based on the crystal structure of compound **7**. It is predicted that the energy required for a single-proton transfer in the double hydrogen bond dimer is very low (the energy barrier is only 11 kcal/mol, see Fig. 3c), whereas that required to break the dimer is much higher (Figure S7). Unfortunately, the crystal structure can be easily crushed into powder by the rubbing process, which makes it impossible for us to obtain a crystal structure after rubbing on the basis of the single-crystal data of **7** and the energy required for the proton transfer. We conclude that the proton transfer process occurs in the double H-bonded dimers of the ortho-pyridinil phenols structure for tautomerism. It can be inferred that the molecule with the oxygen anion in the tautomeric dimer is the bright form, and so contributes to the strong luminescence, as we also note that the pyridium group is normally unfavorable for luminescence effects^54–56^. As a result, we can further confirm that the tautomerism is essentially induced by a single-proton transfer, as a possible double proton transfer (Figure S7) leads to a non-luminescent (PyH^+^) tautomer.

The density functional theory (DFT) and time-dependent density functional theory (TDDFT) calculations based on the crystal structure of 7 were also carried out (Figure S8). The results demonstrated that the intensity of S_1_–S_0_ transition for the initial dimers is extremely small (*f* = 0.01). Thus, the initial crystal state of compound 7 is non-emissive, as the radiative lifetime of S_1_ state is large enough to be quenched through non-radiative pathways. In contrast, the intensity of S_1_–S_0_ transition in the proton-transferred state is very strong (*f* = 0.55, 55 times stronger than the initial state). The frontier molecular orbitals for the initial dimer (both monomers are in neutral state) and the final dimer (one monomer is cationic, and second monomer is anionic) are also presented, including the spectrally active orbitals for individual anionic “bright” species.

To further illuminate the rubbing-induced photoluminescence, a series of materials that can generate positive, neutral, and negative charges upon the triboelectric effect were employed to test the rubbing stimuli response. The efficiency of the stimuli response was determined by the luminescent quantum yield of compound 3 before and after sufficient rubbing (Fig. 3d). It was found that the rubber had the strongest tendency to generate a negative charge (−117 nC/J)^57^, leading to a notable enhancement in the quantum yield relative to the other materials. This result suggests that, to a certain degree, proton transfer in the double H-bonded dimer requires the induction of a negative charge. Meanwhile, the results also indicate that the extra static electric field in the solid state created by rubbing could help stabilize the bright-state tautomer^57,58^, making the reversion to the initial state unfavorable. However, the powder XRD patterns of the sample before and after running shows that a superior crystallinity can be maintained in spite of a packing change (Figure S9). This explains that the topochemical tautomerism is rational and a reversible proton transfer in the dimer is easy as long as the extra static electric field can be dispersed.

**Fig. 4:**
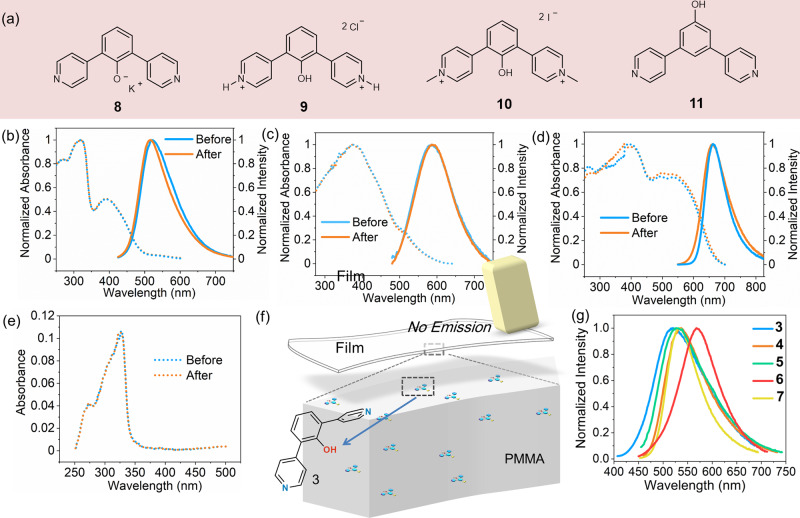
Control study. **a** Chemical structure of reference compounds 8~11. Normalized absorption and emission spectra of **b** 8, **c** 9, **d** 10, and **e** absorption spectra of 11 in their solid-state form; **f** Silent rubbing-induced photoluminescence in the film made from the mixture of compound 3 and PMMA; **g** normalized emission spectra of the rubbed solid samples of 3~7.

More reference compounds (Fig. 4a) were prepared to verify the key mechanisms involved in the proton transfer process. First, compound 3 was mixed with an equivalent ratio of potassium hydroxide to obtain compound 8, which showed similar absorption and emission spectra (see Fig. 4b as well as the luminescent lifetime in Figure S10) as those of 3 following rubbing, further evidencing that the bright form of the tautomeric H-bonded dimer of 3 originated from the molecule with the oxygen anion. Second, compound 3 was fully protonated to prepare compound 9 and eliminate the intermolecular hydrogen bonding. This compound demonstrated weak fluorescence emission, with a quantum yield of 2.5% and the emission peak red-shifting to 588 nm (Fig. 4c). As expected, no changes in the absorption, emission and luminescent lifetime (Figure S10) of this compound were observed upon rubbing. The methylated compound 10 showed a similar tendency towards the optical properties as compound 9 (Fig. 4d). Considering the special angle required to construct the double H-bonded dimer, a bis-para-pyridinil phenol (compound 11) was prepared for comparison. This compound showed weak absorption with the emission quenched in the solid state, both before and after rubbing (Fig. 4e). These control studies demonstrate the significant role of the double H-bonded dimers, which facilitate the proton transfer in the tautomerization process.

The rubbing-induced photoluminescence of 3 becomes less efficient when doped into a PMMA film (Fig. 4f), probably because the important charge contacts and dimeric formation are thereby prevented. In addition, the tautomerization can only be stabilized in the solid state, rather than in the solution. This explains the unchanged resonance of 3 in the liquid ^1^H NMR spectra (Figure S11), but reflects the superior stimuli response at the material level. For the bis-ortho-pyridinil phenol series, the luminescence performance can be effectively adjusted via a change of the substituent group. As is shown in Fig. 3g, a rubbing-induced photoluminescence band with different wavelengths is observed among compounds 3~7. This indicates that a further modification of the H-bonded dimers can be achieved to acquire further material properties.

**Fig. 5:**
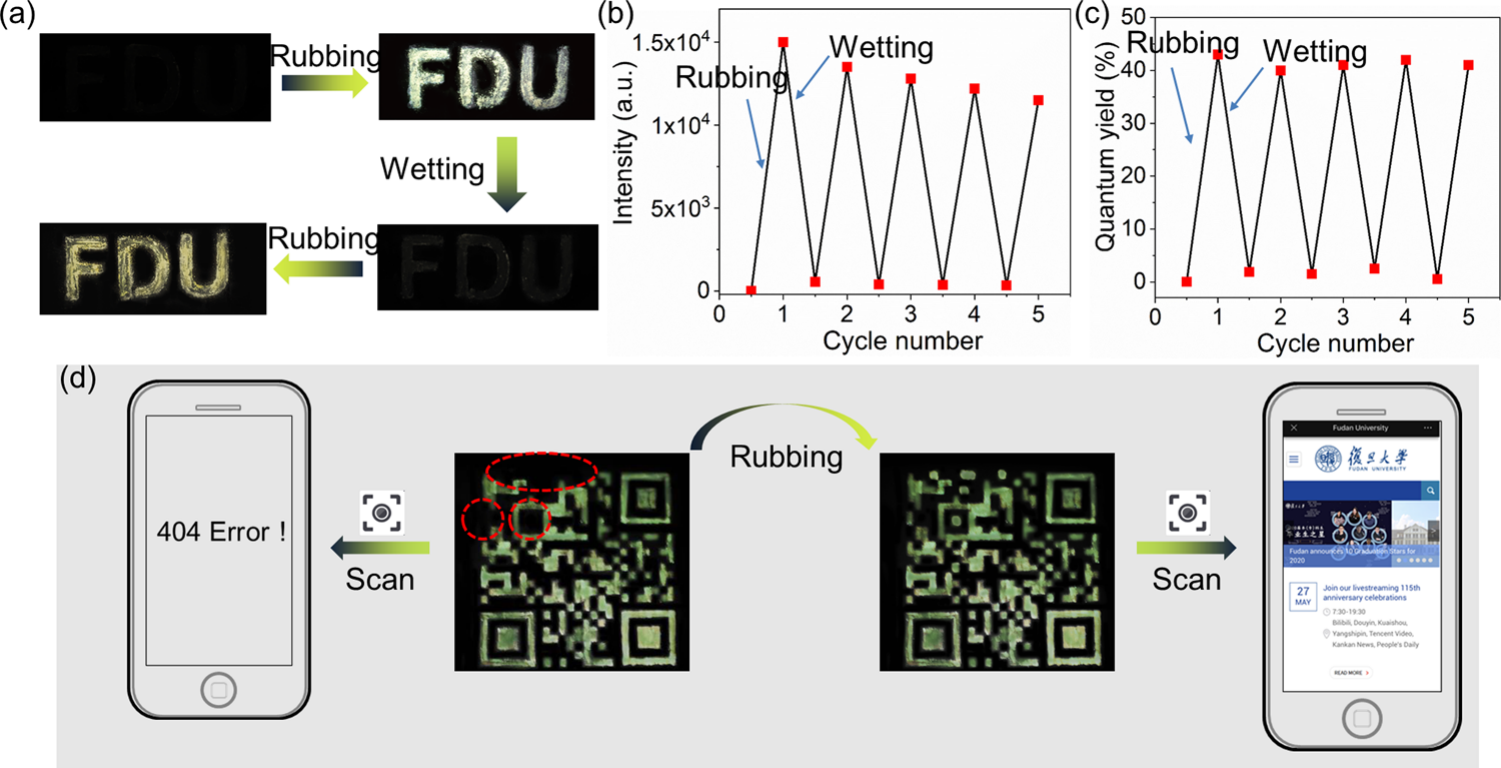
Material application. **a** Photographs under a UV light showing the “FDU” character printed using 3 upon the reversible operation of the rubbing luminescence in the solid state; **b** emission intensity; and **c** luminescent quantum yield of 3 in the solid state with repeated rubbing and wetting for several cycles; **d** photographs of a QR code printed using 3 (for the areas with red circles) and a persulfurated arene as the other routine luminescent material to showcase an application of information encryption.

In principle, the rubbing-induced photoluminescence is reversible. Following investigation, it was found that the simplest and effective method for resetting the rubbing luminescence of 3 is to soak the rubbed solid sample or wipe it with water. Such wetting probably dispersed the electrostatic field created by rubbing and caused the proton transfer back in the tautomerism process. As a result, the material luminescence becomes silent again. After drying has occurred, the rubbing-induced photoluminescence can be remeasured (Fig. 5a). Such a repeated operation can be conducted for a couple of cycles; even though the material luminescent intensity gradually decreased owing to the sample loss during rubbing (Fig. 5b), the quantum yield can be well maintained and feature the stable reproduction of the topochemical reaction (Fig. 5c). The 1H NMR of spectra 3, recovered from water without further purification in the cycling experiment for at least five rounds, shows identical resonance to its fresh sample (Figure S12), further exhibiting chemical structural stability.

Employing such an optical switching approach, we demonstrate an information encryption application with 3. A routinely lab-available luminescent material, persulfurated arene^58,59^, which has an emission band similar to that of 3 after rubbing, was selected to print a QR code on, together with 3. As is shown in Fig. 5d, as the area of 3 has no emission at the initial state (and is highlighted with red circles), the QR code lost its information and became non-functional under UV light excitation. After rubbing, emission was produced in the areas of 3 and thus the complete QR code information could be scanned with a smart phone. Furthermore, a cytotoxicity test demonstrated that the 3 has good biocompatibility (Figure S13). For instance, the 12-hour survival rate of HeLa cells to 2.84 μg/mL of compound 3 was 96.2%. This can provide a good reference for the further development of this material for green and environment-friendly applications in the future.

## Discussion

A green and efficient strategy for rubbing-induced photoluminescence was demonstrated, based on an accurately designed topochemical tautomerism in solid states. This stimuli-responsive behavior could be quantitatively controlled, where the unique double H-bonded dimeric design, with a negative charge (exactly provided by the triboelectric effect of a rubber) induced proton transfer characteristic, played a key role. The advantages of this system are manifold: only rubber can cause such a solid-state topochemical tautomerism, featuring the material choice is selective but still common; the topochemical tautomerism is reversible, allowing a repeated use of the material property; the luminescence quantum yield and wavelength of the system can also be further tuned by structural modification, enabling flexibility in the material property. We believe that the results presented in this work can be of value and make a significant contribution to the fields of chemical methodology and smart-material manufacturing.

## Supplementary information

Supplementary Information

Description of Additional Supplementary Files

Supplementary Movie 1

## Data Availability

The authors declare that all data supporting the findings of this study are available within this article and Supplementary Information files.
