# Supplementary material for: Lighting up solid states using a rubber
Source: Nat Commun. 2021 Feb 10;12:908. doi: 10.1038/s41467-021-21253-w (PMC7876014; doi:10.1038/s41467-021-21253-w)
Supplement: Supplementary file 1 — Supplementary Information [file 41467_2021_21253_MOESM1_ESM.pdf]

Supplementary Information for:

Lighting up Solid States Using a Rubber

Zhongyu Li,[1] Yanjie Wang,[1] Gleb Baryshnikov,[2,3] Shen Shen,[1] Man Zhang,[1] Qi Zou,[4] Hans Ågren,[3,5] Liangliang Zhu*[1]

[1] State Key Laboratory of Molecular Engineering of Polymers, Department of Macromolecular Science, Fudan University, Shanghai 200438, China.

[2] Division of Theoretical Chemistry and Biology School of Biotechnology, KTH Royal Institute of Technology, SE-10691 Stockholm, Sweden

[3] Tomsk State University, 36 Lenin Avenue, Tomsk, Russia

[4] Shanghai Key Laboratory of Materials Protection and Advanced Materials in Electric Power, Shanghai University of Electric Power, Shanghai 200090, China

[5] Department of Physics and Astronomy, Uppsala University, Box 516, SE-751 20 Uppsala, Sweden

[1*] Correspondence to: zhuliangliang@fudan.edu.cn (L. Z.)

Methods

All reagents were commercially available and used as supplied without further purification. Deuterated solvents were purchased from Cambridge Isotope Laboratory (Andover, MA). The ^{1}H NMR, ^{13}C NMR and ^{19}F NMR were measured on a Bruker 400L spectrometer in chloroform-*d* and DMSO-*d6* using tetramethylsilane as internal standard. Mass spectra were recorded on a Matrix Assisted Laser Desorption Ionization-Time of Flight/Time of Flight (MALDI-TOF) Mass Spectrometer (5800). Powder X-ray diffraction data were collected using a Bruker D8 ADVANCE. The UV-Vis absorption spectra were recorded on a Perkin-Elmer Lambda 750 spectrophotometer. Photoluminescence (PL) spectra were collected on an Edinburgh FLS-1000 luminescence spectrometer equipped with a xenon lamp. The PL decay spectra were recorded on Edinburgh FLS-1000 luminescence spectrometer equipped with a microsecond flashlamp as the excitation source (frequency = 100 Hz) and EPL-375 nm picosecond pulsed diode laser as the excitation source for microsecond and time correlated single-photon counting (TCSPC) measurements, respectively. The IRF was measured using silica solution as the reference standard. The absolute fluorescence quantum efficiencies were measured on QM40 with an integrating sphere (φ 150 mm) from Photo Technology International, Inc. (PTI, USA). Single crystal X-ray diffraction signals were collected by a Bruker D8 Venture operating at room temperature, and their structures were resolved and analyzed with the assistance of shelx-2014 software. Optical fluorescence images were taken by using a Nikon microscope at the excitation of UV (365 nm) light.

Molecular geometry optimization was carried out at the DFT level by using the B3LYP exchange-correlation functional[1, 2] and 6-31+G(d) basis set.[3-5] Grimme's empirical dispersion correction (GD3)[6] was employed in all performed calculations. Transition state (TS) calculations were carried by the same GD3-B3LYP/6-31+G(d) method using a synchronous transit-guided quasi-Newton (STQN) algorithm.[7] The relative energies of reference and TS structures were estimated with accounting of zero-point energy and Gibbs free energy corrections. Excited state calculations (vertical absorption and emission singlet-singlet transitions as well as excited states optimization) were performed by TDDFT method[8, 9] using B3LYP/6-31+G(d) approach. For better describing the twisted charge-transfer S_1 state in anionic compound 7, the long-range corrected CAM-B3LYP functional[10] was used together with 6-31+G(d) basis set. All the calculations were carried out using the Gaussian 16 (Revision A.03) program package[11].

Synthesis and characterizations of compounds.

Synthesis of compound **1**

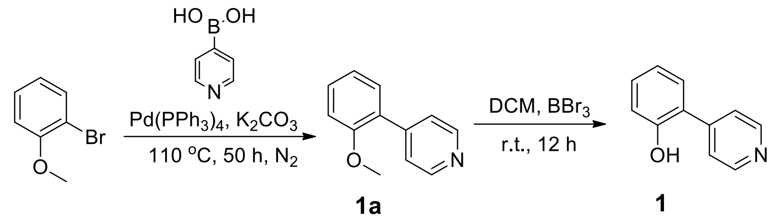

The synthesis of 1a. 1-bromo-2-methoxybenzene (935 g, 5 mmol), pyridin-4-yl boronic acid (1.23 g, 10 mmol), and $Pd(PPh_3)_4$ (577 mg, 0.5 mmol), K_2CO_3 (1.38 g, 10 mmol) were dissolved in a mixture of DMF (50 mL) / H_2O (2 mL). The mixture was stirred at 110 °C for 50 h under nitrogen. After cooling to room temperature, the mixture was poured into water (200 mL) and extracted three times with CH_2Cl_2. The organic layer was dried over anhydrous Na_2SO_4. After removing the solvent under reduced pressure, the residue was chromatographed on a silica gel column with CH_2Cl_2. 786 mg white solid **1a** was obtained, yield: 85%. ^{1}H NMR (400 MHz, $CDCl_3$) δ 8.61 (dd, *J* = 4.5, 1.6 Hz, 4H), 7.47 (dd, *J* = 4.5, 1.6 Hz, 4H), 7.39 (ddd, *J* = 8.2, 7.5, 1.8 Hz, 1H), 7.34 (dd, *J* = 7.6, 1.7 Hz, 1H), 7.06 (td, *J* = 7.5, 1.0 Hz, 1H), 7.01 (d, *J* = 8.3 Hz, 1H), 3.83 (s, 3H). ^{13}C NMR (101 MHz, $CDCl_3$) δ 156.7, 149.6, 146.5, 130.6, 130.3, 127.8, 124.4, 121.2, 111.6, 55.7.

The synthesis of compound 1. **1a** (370 mg, 2 mmol) was dissolved into 30 mL fresh distilled DCM in a 100 mL schlenk flask, the system was cooling down to 0 °C by a mixture of ice and water, then 10 mL 1M DCM solution of BBr_3 was dropped slowly into the system, the solution was stirred for 5 min and then allowed to worm to room temperature and further stirred overnight, under argon atmosphere. 100 mL of 10% $NaHCO_3$ aqueous solution was added into the resulting solution. Then the mixture was neutralized to pH = 8~9, the precipitated was collected and further purified by column (MeOH/DCM : *v*/*v* = 30/1). A light pink powder (222 mg) was obtained, yield = 65%. ^{1}H NMR (400 MHz, DMSO) δ 10.54 (s, 1H), 8.89 (s, 2H), 8.29 (d, *J* = 4.5 Hz, 2H), 7.59 (d, *J* = 7.1 Hz, 1H), 7.39 (d, *J* = 6.8 Hz, 1H), 7.11 (d, *J* = 7.8 Hz, 1H), 7.00 (t, *J* = 6.8 Hz, 1H). ^{13}C NMR (101 MHz, DMSO) δ 155.8, 154.7, 141.6, 132.7, 130.7, 126.0, 121.7, 120.1, 116.9.

Synthesis of compound **2**

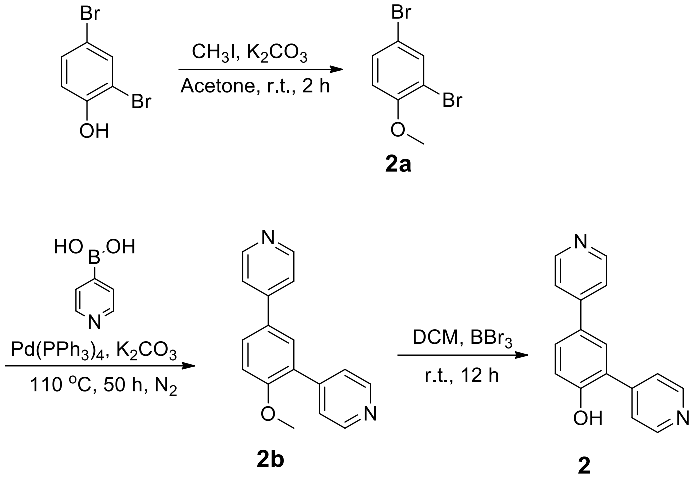

The synthesis of 2a. 2,6-dibromophenol (2.52 g, 10 mmol) and K_2CO_3 (2.76 g, 20 mmol) were dropped into a 100 mL round bottle flask, then, 50 mL acetone and iodomethane (2.8 g, 1.25 mL, 20 mmol) was also injected into the system, the reaction was carried under room temperature for 2 h. The solvent was removed under reduce pressure and purified by column (Hexane), 2.47 g of clear liquid was obtained, yield = 93%, ^{1}H NMR (400 MHz, $CDCl_3$) δ 7.66 (d, *J* = 2.4 Hz, 1H), 7.38 (m, 1H), 6.76 (d, *J* = 8.8 Hz, 1H), 3.87 (s, 3H). ^{13}C NMR (101 MHz, $CDCl_3$) δ 155.3, 135.5, 131.3, 113.2, 112.9, 112.6, 56.5.

The synthesis of 2b. **2a** (1.33 g, 5 mmol), pyridin-4-yl boronic acid (1.85 g, 15 mmol), and $Pd(PPh_3)_4$ (577 mg, 0.5 mmol), K_2CO_3(2.76 g, 20 mmol) were dissolved in a mixture of DMF (50 mL) / H_2O (2 mL). The mixture was stirred at 110 °C for 50 h under nitrogen. After cooling to room temperature, the mixture was poured into water (200 mL) and extracted three times with CH_2Cl_2. The organic layer was dried over anhydrous Na_2SO_4. After removing the solvent under reduced pressure, the residue was chromatographed on a silica gel column with CH_2Cl_2/MeOH (100:1, v/v). 668 mg white solid **2b** was obtained, yield: 51%. m.p. 204.5-205.9 °C. ^{1}H NMR (400 MHz, $CDCl_3$) δ 8.68 (d, *J* = 5.2 Hz, 4H), 7.54 (d, *J* = 6.0 Hz, 4H), 7.23 (s, 2H), 3.16 (s, 3H), 2.42 (s, 3H). ^{13}C NMR (101 MHz, $CDCl_3$) δ 153.1, 149.9, 146.2, 134.6, 133, 131.8, 124.1, 61.2, 20.9.

The synthesis of compound 2. **2b** (524 mg, 2 mmol) was dissolved into 30 mL of fresh distilled DCM in a 100 mL schlenk flask, the system was cooling down to 0 °C by a mixture of ice and water, then 10 mL of 1M DCM solution of BBr_3 was dropped slowly into the system, the solution was stirred for 5 min and then allowed to worm to room temperature and further stirred overnight, under argon atmosphere. 100 mL of 10% $NaHCO_3$ aqueous solution was added into the resulting solution. Then the mixture was neutralized to pH = 8~9, the precipitated was collected and further purified by column (MeOH/DCM : *v*/*v* = 30/1). A light pink powder (188 mg) was obtained, yield = 38%. ^{1}H NMR (400 MHz, $CDCl_3/CD_3OD$) δ 8.49 (dd, *J* = 4.6, 1.6 Hz, 2H), 8.45 (dd, *J* = 4.6, 1.6 Hz, 2H), 7.58 (dd, *J* = 4.6, 1.6 Hz, 2H), 7.54 (d, *J* = 2.3 Hz, 1H), 7.50 – 7.44 (m, 3H), 7.02 – 6.99 (m, 1H). ^{13}C NMR (101 MHz, $CDCl_3$/MeOD) δ 156.1, 149.3, 148.7, 148.5, 147.0, 129.2, 128.9, 128.7, 126.0, 124.5, 121.2, 117.1.

Synthetic route of compound **3**

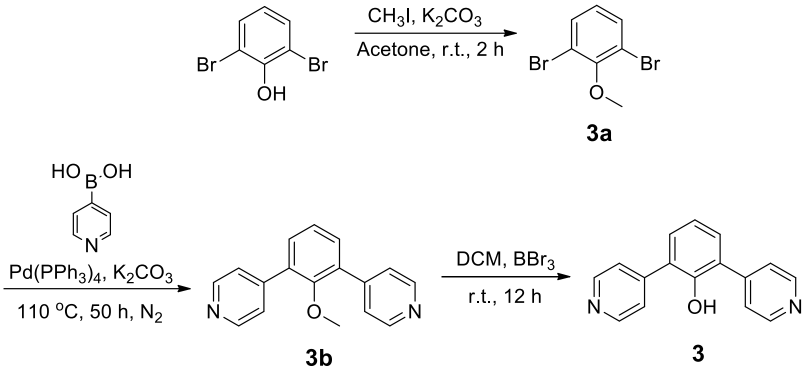

The synthesis of 3a. 2,6-dibromophenol (2.52 g, 10 mmol) and K_2CO_3 (2.76 g, 20 mmol) were dropped into a 100 mL round bottle flask, then, 50 mL of acetone and iodomethane (2.8 g, 1.25 mL, 20 mmol) were also injected into the system, the reaction was carried under room temperature for 2 h. The solvent was removed under reduce pressure and purified by column (hexane), 2.47 g of clear liquid was obtained, yield = 93%, ^{1}H NMR (400 MHz, $CDCl_3$) δ 7.49 (d, J = 8.0 Hz, 2H), 6.86 (t, J = 8.0 Hz, 1H), 3.89 (s, 3H). ^{13}C NMR (101 MHz, $CDCl_3$) δ 154.3, 132.8, 126.4, 118.4, 60.7.

The synthesis of 3b. **3a** (1.33 g, 5 mmol), pyridin-4-yl boronic acid (1.85 g, 15 mmol), and $Pd(PPh_3)_4$ (577 mg, 0.5 mmol), K_2CO_3(2.76 g, 20 mmol) were dissolved in a mixture of DMF (50 mL) / H_2O (2 mL). The mixture was stirred at 110 °C for 48 h under nitrogen. After cooling to room temperature, the mixture was poured into water (200 mL) and extracted three times with CH_2Cl_2. The organic layer was dried over anhydrous Na_2SO_4. After removing the solvent under reduced pressure, the residue was chromatographed on a silica gel column with CH_2Cl_2/MeOH (100:1, v/v), 773 mg white solid **3b** was obtained, Yield: 59%. ^{1}H NMR (400 MHz, $CDCl_3$) δ 8.70 (dd, J = 4.5, 1.6 Hz, 4H), 7.54 (dd, J = 4.5, 1.7 Hz, 4H), 7.45 (t, J = 1.5 Hz, 1H), 7.22 (d, J = 1.5 Hz, 2H), 3.95 (s, 3H). 132.5 **-** 133.6 °C. ^{13}C NMR (101 MHz, $CDCl_3$) δ 155.8, 149.9, 146.0, 133.4, 131.3, 125.1, 124.1, 61.2. m/z: $[M+H]^+$, 263.1121.

The synthesis of compound 3. **3b** (524 mg, 2 mmol) was dissolved into 30 mL fresh distilled DCM in a 100 mL schlenk flask, the system was cooling down to 0 °C by a mixture of ice and water, then 10 mL 1M DCM solution of BBr_3 was dropped slowly into the system, the solution was stirred for 5 min and then allowed to worm to room temperature and further stirred overnight, under argon atmosphere. 100 mL 10% $NaHCO_3$ aqueous solution was added into the resulting solution. Then the mixture was neutralized to pH = 8~9, the precipitated was collected and further purified by column (MeOH/DCM : *v*/*v* = 30/1). A light pink powder (228 mg) was obtained, yield = 46%. m.p. 209.9 – 211.5 °C. ^{1}H NMR (400 MHz, DMSO) δ 8.99 (s, 1H), 8.62 (d, J = 5.6 Hz, 4H), 7.57 (d, J = 5.6 Hz, 4H), 7.38 (d, J = 7.6 Hz, 2H), 7.13 (t, J = 7.6 Hz, 1H). ^{13}C NMR (101 MHz, DMSO) δ 150.9, 149.5, 146.0, 130.9, 128.8, 124.2, 121.3. m/z: $[M+H]^+$, 249.1045.

Synthesis of compound **4**

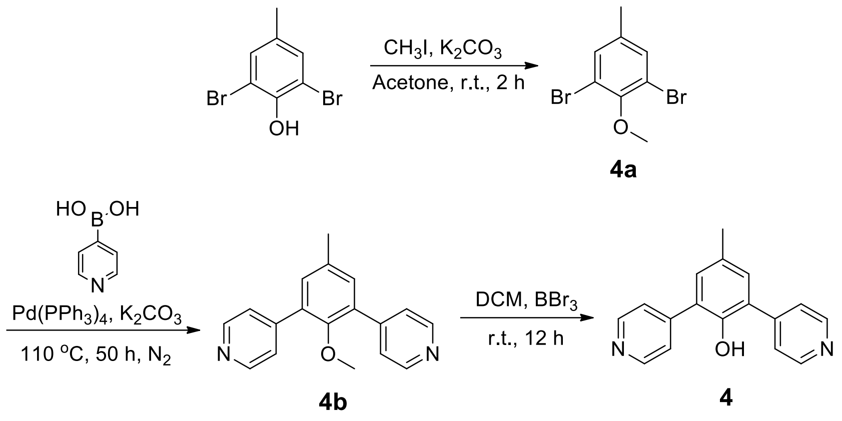

The synthesis of 4a. 2,6-dibromo-4-methylphenol (2.66 g, 10 mmol) was dropped into a 100 mL round bottle flask, then, 50 mL acetone and iodomethane (2.8 g, 1.25 mL, 20 mmol) was also injected into the system, the reaction was carried under nitrogen for 12 hours. The solvent was removed under reduce pressure and purified by column (Hexane), 2.72 g clear liquid was obtained, yield = 98%, ^{1}H NMR (400 MHz, $CDCl_3$) δ 7.31 (s, 2H), 3.85 (s, 3H), 2.28 (s, 3H). ^{13}C NMR (101 MHz, $CDCl_3$) δ 151.9, 136.5, 133.1, 117.6, 60.6, 20.3.

The synthesis of 4b. **4a** (1.4 g, 5 mmol), (1.85 g, 15 mmol), and $Pd(PPh_3)_4$ (577 mg, 0.5 mmol), K_2CO_3(2.76 g, 20 mmol) were dissolved in a mixture of DMF (50 mL) / H_2O (2 mL). The mixture was stirred at 110 °C for 48 h under nitrogen. After cooling to room temperature, the mixture was poured into water (200 mL) and extracted three times with CH_2Cl_2. The organic layer was dried over anhydrous Na_2SO_4. After removing the solvent under reduced pressure, the residue was chromatographed on a silica gel column with CH_2Cl_2/MeOH (100:1, v/v), 814 mg white solid **4c** was obtained, Yield: 59%. m.p. 204.5 - 205.9 °C. ^{1}H NMR (400 MHz, $CDCl_3$) δ 8.68 (d, J = 5.2 Hz, 4H), 7.54 (d, J = 6.0 Hz, 4H), 7.23 (s, 2H), 3.16 (s, 3H), 2.42 (s, 3H). ^{13}C NMR (101 MHz, $CDCl_3$) δ 153.1, 149.9, 146.2, 134.6, 133, 131.8, 124.1, 61.2, 20.9. m/z: $[M+H]^+$, 277.1342.

The synthesis of compound 4. **4c** (522 mg, 2 mmol) was dissolved into 30 mL fresh distilled DCM in a 100 schlenk flask, the system was cooling down to 0 °C by a mixture of ice and water, then 10 mL 1M DCM solution of BBr_3 was dropped slowly into the system, the solution was stirred for 5 min and then allowed to worm to room temperature and further stirred overnight, under argon atmosphere. 100 mL 10% $NaHCO_3$ aqueous solution was added into the resulting solution. Then the mixture was neutralized to pH = 8~9, the precipitated was collected and further purified by column (MeOH/DCM : *v*/*v* = 30/1). A light yellow powder (241 mg) was obtained, yield = 46%. m.p. 205.3-206.8 °C. ^{1}H NMR (400 MHz, $CDCl_3$) δ 8.56 (d, *J* = 4.4 Hz, 4H), 7.48 (d, *J* = 6.0 Hz, 4H), 7.14 (s, 2H), 6.76 (s, 1H), 2.38 (s, 3H). ^{13}C NMR (101 MHz, $CDCl_3$) δ 149.5, 148.5, 146.8, 131.6, 130.7, 127.8, 124.5, 20.6.

m/z: $[M+H]^+$, 263.1208.

Synthetic route of compound **5**

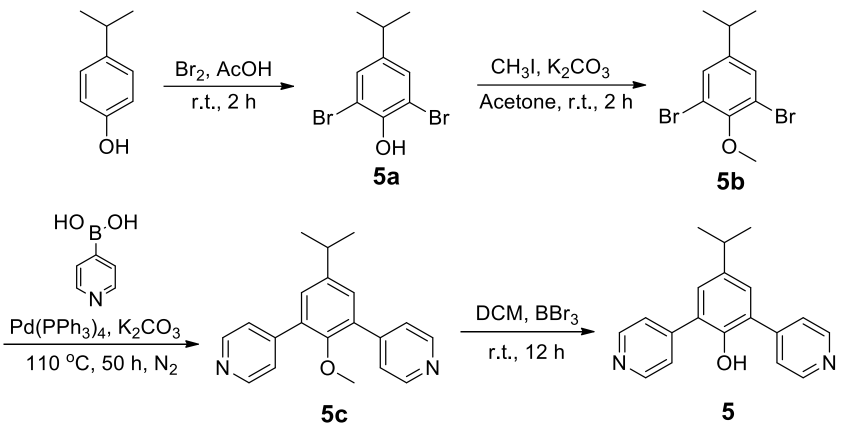

The synthesis of 5a. 4-isopropylphenol (1.36 g, 10 mmol) was dropped into a 100 mL round bottle flask, then, 50 mL acetic acid was injected into the system. After cooling down to 0 ~ 4 °C by ice bath, 3.52 g (22 mmol) Br_2 was dropped slowly into the system. The solution was stirred for 5 min and then allowed to worm to room temperature and further stirred for 2 hours. The solvent was removed under reduce pressure and purified by column (Hexane), 2.6 g clear liquid was obtained, yield = 89%, ^{1}H NMR (400 MHz, $CDCl_3$) δ 7.29 (s, 2H), 5.73 (s, 1H), 2.80 (hept, *J* = 6.9 Hz, 1H), 1.21 – 1.19 (d, *J* = 8 Hz, 6H). ^{13}C NMR (101 MHz, $CDCl_3$) δ 147.3, 143.6, 130.1, 109.7, 33.2, 24.0.

The synthesis of 5b. **5a** (1.47 g, 5 mmol) and K_2CO_3 (1.38 g, 10 mmol) was dropped into a 100 mL round bottle flask, then, 50 mL acetone and iodomethane (2.8 g, 1.25 mL, 20 mmol) was also injected into the system, the reaction was carried under nitrogen for 2 hours. The solvent was removed under reduce pressure and purified by column (Hexane), 1.43 g clear liquid was obtained, yield = 93%, ^{13}C NMR (101 MHz, $CDCl_3$) δ 152.0, 147.5, 130.7, 117.9, 60.6, 33.4, 23.8.

The synthesis of 5c. **5b** (924 mg, 3 mmol), pyridin-4-yl boronic acid (1.47 g, 12 mmol), and $Pd(PPh_3)_4$ (350 mg, 303 μmol), K_2CO_3(2.07 g, 15 mmol) were dissolved in a mixture of DMF (50 mL) / H_2O (2 mL). The mixture was stirred at 110 °C for 50 h under nitrogen. After cooling to room temperature, the mixture was poured into water (200 mL) and extracted three times with CH_2Cl_2. The organic layer was dried over anhydrous Na_2SO_4. After removing the solvent under reduced pressure, the residue was chromatographed on a silica gel column with CH_2Cl_2/MeOH (100:1, v/v), 538 mg white solid **8** was obtained, Yield: 59%. ^{1}H NMR (400 MHz, $CDCl_3$) δ 8.66 (dd, *J* = 4.5, 1.6 Hz, 4H), 7.54 (dd, *J* = 4.5, 1.6 Hz, 4H), 7.25 (s, 2H), 3.16 (s, 3H), 2.97 (hept, *J* = 6.9 Hz, 1H), 1.29 (d, *J* = 6.9 Hz, 2H). ^{13}C NMR (101 MHz, $CDCl_3$) δ 153.2, 149.9, 146.5, 145.6, 133.0, 129.3, 124.2, 61.1, 33.8, 24.1. m/z: $[M+H]^+$, 305.1651.

The synthesis of compound 5. **5c** (304 mg, 1 mmol) was dissolved into 30 mL fresh distilled DCM in

a 100 mL schlenk flask, the system was cooling down to 0 °C by a mixture of ice and water, then 10 mL 1M DCM solution of BBr_3 was dropped slowly into the system, the solution was stirred for 5 min and then allowed to worm to room temperature and further stirred overnight, under argon atmosphere. 100 mL 10% $NaHCO_3$ aqueous solution was added into the resulting solution. Then the mixture was neutralized to pH = 8~9, the precipitated was collected and further purified by column (MeOH/DCM : *v*/*v* = 30/1). A light yellow powder (133 mg) was obtained, yield = 46%. ^{1}H NMR (400 MHz, $CDCl_3$) δ 8.48 (s, 4H), 7.54 – 7.45 (m, 4H), 7.17 (s, 2H), 2.99 – 2.85 (m, 1H), 1.28 (d, *J* = 6.9 Hz, 6H). ^{13}C NMR (101 MHz, $CDCl_3$) δ 156.6, 149.7, 146.8, 141.95 (s), 141.9, 129.0, 124.5, 33.5, 24.3. m/z: $[M+H]^+$, 291.1502.

Synthesis of compound **6**

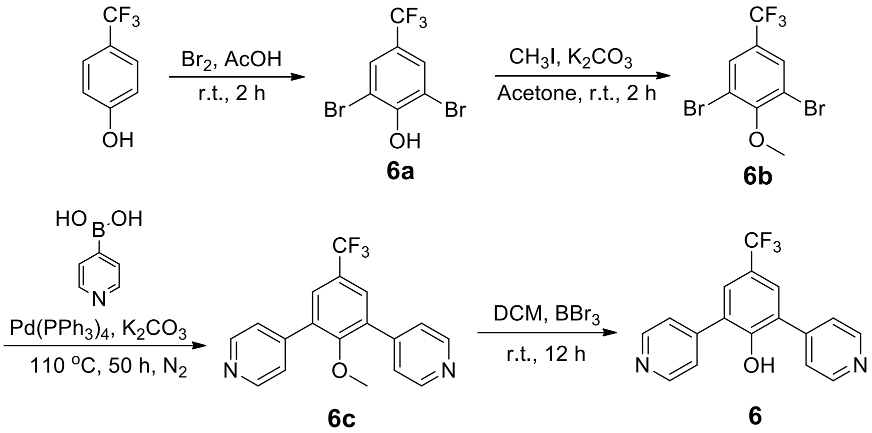

The synthesis of 6. 4-(trifluoromethyl)phenol (1.62 g, 10 mmol) was dropped into a 100 mL round bottle flask, then, 50 mL acetic acid was injected into the system. After cooling down to 0 ~ 4 °C by ice bath, 3.52 g (22 mmol) Br_2 was dropped slowly into the system. The solution was stirred for 5 min and then allowed to worm to room temperature and further stirred for 2 hours. The solvent was removed under reduce pressure and purified by column (Hexane), 2.85 g clear liquid was obtained, yield = 89%, ^{1}H NMR (400 MHz, $CDCl_3$) δ 7.73 (s, 2H), 6.21 (s, 1H). ^{13}C NMR (101 MHz, $CDCl_3$) δ 154.50 (t, *J* = 1.0 Hz), 129.37 (q, *J* = 3.8 Hz), 122.64 (dq, *J* = 330.4, 272.1 Hz), 122.50 (q, *J* = 33.5 Hz), 111.9.

The synthesis of 6b. **6a** (1.6 g, 5 mmol) and K_2CO_3 (1.38 g, 10 mmol) was dropped into a 100 mL round bottle flask, then, 50 mL acetone and iodomethane (2.8 g, 1.25 mL, 20 mmol) was also injected into the system, the reaction was carried under room temperature for 2 hours. The solvent was removed under reduce pressure and purified by column (Hexane), 1.42 g clear liquid was obtained, yield = 85%, ^{1}H NMR (400 MHz, $CDCl_3$) δ 7.77 (d, *J* = 0.6 Hz, 2H), 3.93 (s, 6H). ^{13}C NMR (101 MHz, $CDCl_3$) δ 157.35 (d, *J* = 1.2 Hz), 130.13 – 130.00 (m), 128.72 (d, *J* = 34.0 Hz), 126.64 – 118.23 (m), 60.82 (d, *J* = 0.5 Hz).

The synthesis of 6c. **6b** (1 g, 3 mmol), pyridin-4-yl boronic acid (1.47 g, 12 mmol), and $Pd(PPh_3)_4$

(350 mg, 303 μmol), K_2CO_3(2.07 g, 15 mmol) were dissolved in a mixture of DMF (50 mL) / H_2O (2 mL). The mixture was stirred at 110 °C for 50 h under nitrogen. After cooling to room temperature, the mixture was poured into water (200 mL) and extracted three times with CH_2Cl_2. The organic layer was dried over anhydrous Na_2SO_4. After removing the solvent under reduced pressure, the residue was chromatographed on a silica gel column with CH_2Cl_2/MeOH (100:1, v/v), 614 mg white solid **6c** was obtained, Yield: 62%. m.p. 165.1 - 167.8 °C. ^{1}H NMR (400 MHz, $CDCl_3$) δ 8.72 (br, 4H), 7.67 (br, 4H), 7.54 (br, 2H), 3.24 (s, 1H). ^{13}C NMR (101 MHz, $CDCl_3$) δ 157.9, 150.3, 144.8, 134.2, 128.3, 123.9, 61.5. m/z: $[M+H]^+$, 331.1073.

The synthesis of compound 6. **6c** (330 mg, 1 mmol) was dissolved into 30 mL fresh distilled DCM in a 100 schlenk flask, the system was cooling down to 0 °C by a mixture of ice and water, then 10 mL 1M DCM solution of BBr_3 was dropped slowly into the system, the solution was stirred for 5 min and then allowed to worm to room temperature and further stirred overnight, under argon atmosphere. 100 mL 10% $NaHCO_3$ aqueous solution was added into the resulting solution. Then the mixture was neutralized to pH = 8~9, the precipitated was collected and further purified by column (MeOH/DCM : *v*/*v* = 30/1). A light yellow powder (73 mg) was obtained, yield = 23%. m.p. 175.6 – 177.8 °C. ^{1}H NMR (400 MHz, DMSO) δ 8.66 (dd, *J* = 4.5, 1.6 Hz, 4H), 7.68 (d, *J* = 0.6 Hz, 2H), 7.62 (dd, *J* = 4.5, 1.6 Hz, 4H). ^{13}C NMR (101 MHz, DMSO) δ 154.96 – 154.92 (m), 150.1, 145.1, 129.6, 129.02 – 128.94 (m), 128.07 (d, *J* = 3.7 Hz), 126.08 – 125.97 (m), 124.7, 123.37 – 123.27 (m), 121.90 (d, *J* = 32.4 Hz). ^{19}F NMR (376 MHz, DMSO) δ -59.91 (s). m/z: $[M+H]^+$, 317.0895.

Synthesis of compound **7**

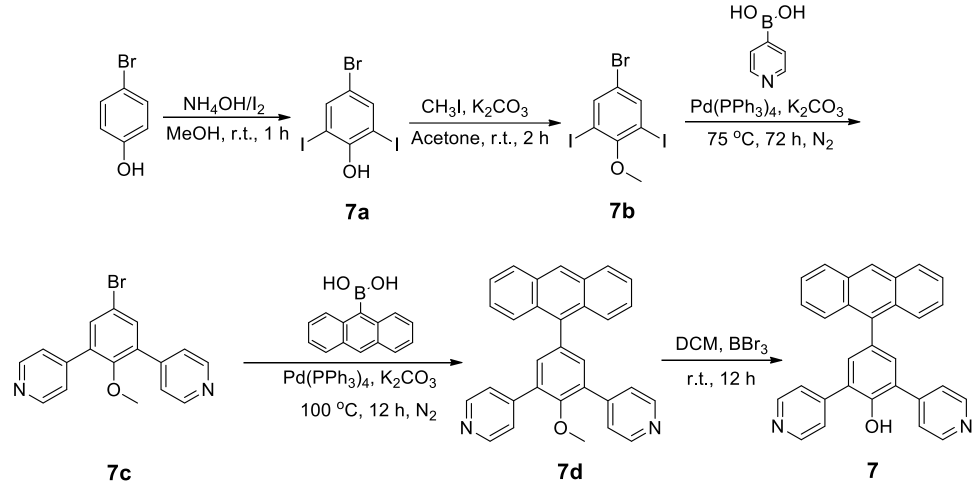

The synthesis of 7a. In a 500 mL round bottom flask equipped with magnetic stirring bar and dropping funnel, 4-bromophenol (8.65 g, 50 mmol) was dissolved in methanol (50 mL), 20% ammonium hydroxide (50 mL) was added and solution of iodine (27.9 g; 110 mmol) in methanol (50 mL) was added dropwise. The mixture was stirred at room temperature until the substrate was consumed, as judged by TLC (~2h). Methanol was removed by rotary evaporation, water (100 mL) was added and the mixture was acidified with 2M HCl to pH=2. Product was extracted with ethyl

acetate (3×100 mL). The combined extracts were washed with water, 5% aqueous sodium thiosulfate solution, brine and dried with anhydrous magnesium sulfate. Solvent was removed by rotary evaporation and the crude product was purified by recrystallization. 19.7 g white solid was obtained (93% yield). ^{1}H NMR (400 MHz, $CDCl_3$) δ 7.78 (s, 2H), 5.74 (s, 1H). ^{13}C NMR (101 MHz, $CDCl_3$) δ 153.3, 141.0, 113.7, 82.6.

The synthesis of 7b. **7a** (16.9 g, 40 mmol) and K_2CO_3 (13.8 g, 100 mmol) was dropped into a 250 mL round bottle flask, then, 100 mL acetone and iodomethane (14 g, 6.25 mL, 100 mmol) was also injected into the system, the reaction was carried under nitrogen for 12 hours. The solvent was removed under reduce pressure and purified by column (Hexane), 16.3 g white solid was obtained, yield = 93%, ^{1}H NMR (400 MHz, $CDCl_3$) δ 7.88 (s, 2H), 3.83 (s, 3H). ^{13}C NMR (101 MHz, $CDCl_3$) δ 158.6, 141.8, 118.4, 91.0, 60.9.

The synthesis of 7c. **7b** (4.39 g, 10 mmol), pyridin-4-yl boronic acid (2.7 g, 22 mmol), and $Pd(PPh_3)_4$ (1.15 g, 1 mmol), K_2CO_3(5.5 g, 40 mmol) were dropped into a 100 mL Schlenk flask and dissolved in a mixture of DMF (50 mL) / H_2O (2 mL). The mixture was stirred at 75 ^oC for 72 h under nitrogen. After cooling to room temperature, the mixture was poured into water (200 mL) and extracted three times with CH_2Cl_2. The organic layer was dried over anhydrous Na_2SO_4. After removing the solvent under reduced pressure, the residue was chromatographed on a silica gel column with CH_2Cl_2/MeOH (100:1, v/v), 1.2 g white solid was obtained, Yield: 35%. ^{1}H NMR (400 MHz, $CDCl_3$) δ 8.69 (dd, *J* = 4.5, 1.6 Hz, 4H), 7.54 (s, 2H), 7.51 (dd, *J* = 4.5, 1.6 Hz, 4H), 3.16 (s, 3H). ^{13}C NMR (101 MHz, $CDCl_3$) δ 153.1, 149.9, 146.2, 134.6, 133, 131.8, 124.1, 61.2, 20.9.

The synthesis of 7d. **7c** (341 mg, 1 mmol), anthracen-9-ylboronic acid (444 mg, 2 mmol), and $Pd(PPh_3)_4$ (56 mg, 50 μmol), K_2CO_3(345 mg, 2.5 mmol) were dissolved in a mixture of DMF (25 mL) / H_2O (1 mL). The mixture was stirred at 110 ^oC for 12 h under nitrogen. After cooling to room temperature, the mixture was poured into water (100 mL) and extracted three times with CH_2Cl_2. The organic layer was dried over anhydrous Na_2SO_4. After removing the solvent under reduced pressure, the residue was chromatographed on a silica gel column with CH_2Cl_2/MeOH (100:1, v/v), 302 mg light yellow solid **7d** was obtained, Yield: 69%. ^{1}H NMR (400 MHz, $CDCl_3$) δ 8.68 (dd, *J* = 4.6, 1.6 Hz, 4H), 8.53 (s, 1H), 8.07 (d, *J* = 8.4 Hz, 2H), 7.79 (dd, *J* = 8.7, 0.8 Hz, 2H), 7.66 (dd, *J* = 4.5, 1.6 Hz, 4H), 7.53 (s, 2H), 7.52 – 7.39 (m, 4H), 3.38 (s, 3H). ^{13}C NMR (101 MHz, $CDCl_3$) δ 154.8, 150.1, 145.8, 135.8, 135.0, 134.1, 133.4, 131.5, 130.4, 128.7, 127.4, 126.4, 126.0, 125.4, 124.2, 61.4.

The synthesis of compound 7. **7d** (219 mg, 0.5 mmol) was dissolved into 30 mL fresh distilled DCM in a 100 schlenk flask, the system was cooling down to 0 ^oC by a mixture of ice and water, then 10 mL 1M DCM solution of BBr_3 was dropped slowly into the system, the solution was stirred for 5 min and then allowed to worm to room temperature and further stirred overnight, under argon atmosphere. 100 mL 10% $NaHCO_3$ aqueous solution was added into the resulting solution. Then the mixture was neutralized to pH = 8~9, the precipitated was collected and further purified by column (MeOH/DCM :

v/*v* = 30/1). A light yellow powder (116 mg) was obtained, yield = 55%. ^{1}H NMR (400 MHz, $CDCl_3$) δ 8.51 (s, 1H), 8.46 (d, *J* = 5.7 Hz, 4H), 8.05 (d, *J* = 8.4 Hz, 2H), 7.81 (t, *J* = 9.0 Hz, 2H), 7.61 (d, *J* = 5.8 Hz, 4H), 7.50 – 7.37 (m, 6H). ^{13}C NMR (101 MHz, $CDCl_3$) δ 150.8, 149.7, 146.4, 135.4, 133.8, 131.9, 131.5, 130.6, 128.7, 128.0, 127.1, 126.6, 125.9, 125.3, 124.6. m/z: $[M+H]^+$, 425.1646.

Synthesis of compound **8**

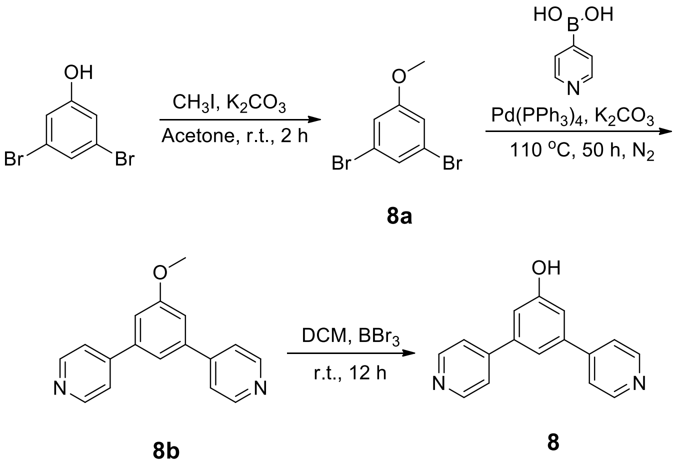

The synthesis of 8a. 3,5-dibromophenol (2.52 g, 10 mmol) and K_2CO_3 (2.76 g, 20 mmol) was dropped into a 100 mL round bottle flask, then, 50 mL acetone and iodomethane (2.8 g, 1.25 mL, 20 mmol) was also injected into the system, the reaction was carried under room temperature for 2 hours. The solvent was removed under reduce pressure and purified by column (Hexane), 2.44 g clear liquid was obtained, yield = 92%, ^{1}H NMR (400 MHz, $CDCl_3$) δ 7.24 (t, *J* = 1.6 Hz, 1H), 6.98 (d, *J* = 1.6 Hz, 2H), 3.77 (s, 3H).

The synthesis of 8b. **8a** (1.33 g, 5 mmol), pyridin-4-yl boronic acid (1.85 g, 15 mmol), and $Pd(PPh_3)_4$ (577 mg, 0.5 mmol), K_2CO_3(2.76 g, 20 mmol) were dissolved in a mixture of DMF (50 mL) / H_2O (2 mL). The mixture was stirred at 110 °C for 48 h under nitrogen. After cooling to room temperature, the mixture was poured into water (200 mL) and extracted three times with CH_2Cl_2. The organic layer was dried over anhydrous Na_2SO_4. After removing the solvent under reduced pressure, the residue was chromatographed on a silica gel column with CH_2Cl_2/MeOH (100:1, v/v), 851 mg white solid **8b** was obtained, Yield: 65%. m.p. 155.1-156.3 °C. ^{1}H NMR (400 MHz, $CDCl_3$) δ 8.74 (dd, *J* = 4.5, 1.5 Hz, 4H), 8.02 (s, 2H), 7.90 (s, 1H), 7.62 (s, 1H), 7.60 (dd, *J* = 4.5, 1.6 Hz, 4H), 3.26 (s, 3H). ^{13}C NMR (101 MHz, $CDCl_3$) δ 160.8, 150.4, 147.8, 140.6, 121.8, 118.3, 113.2, 55.7.

The synthesis of compound 8. **8b** (524 mg, 2 mmol) was dissolved into 30 mL fresh distilled DCM in a 100 mL schlenk flask, the system was cooling down to 0 °C by a mixture of ice and water, then 10 mL 1M DCM solution of BBr_3 was dropped slowly into the system, the solution was stirred for 5 min and then allowed to worm to room temperature and further stirred overnight, under argon atmosphere.

100 mL 10% $NaHCO_3$ aqueous solution was added into the resulting solution. Then the mixture was neutralized to pH = 8~9, the precipitated was collected and further purified by column (MeOH/DCM : *v*/*v* = 30/1). A white solid (104 mg) was obtained, yield = 21%. m.p. > 250 ℃. ^{1}H NMR (400 MHz, DMSO-d_6) δ 8.67 (d, J = 6.0 Hz, 4H), 7.79 (d, *J* = 6.0 Hz, 4H), 7.62 (s, 1H), 7.26 (s, 2H). ^{13}C NMR (101 MHz, DMSO-d_6) δ 159.2, 150.4, 147.6, 140.0, 122.0, 116.9, 114.9.

Synthesis of compound **10**

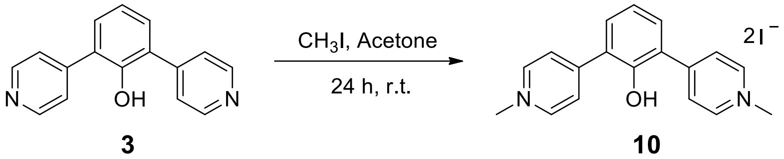

The synthesis of compound 10. **Compound 3** (145 mg, 0.5 mmol) and iodomethane (0.7 g, 0.31 mL, 5 mmol) was dropped into a 100 mL round bottle flask, then, 50 mL acetone was also injected into the system, the reaction was carried under room temperature for 24 hours. The solvent was removed under reduce pressure and washed by DCM, 202 mg red solid was obtained, yield = 85%, ^{1}H NMR (400 MHz, D_2O) δ 8.79 (d, *J* = 4.2 Hz, 4H), 8.28 (br, 4H), 7.62 (s, 2H), 4.41 (s, 6H), 3.04 (br, 1H), 1.29 (d, *J* = 6.1 Hz, 6H). ^{13}C NMR (101 MHz, $CDCl_3$) δ 151.9, 136.5, 133.1, 117.6, 60.6, 20.3.

Table S1. The optical data of compounds **1-10** before and after rubbing.

**Comp.**	λ_{exc}/ nm	λ_{emiss}/nm(Before)	Φ/%(Before)	λ_{emiss}/nm(After)	Φ/%(After)
**1**	365	-	-	-	-
**2**	365	-	-	480	23
**3**	390	-	-	525	45
**4**	410	-	-	535	43
**5**	425	-	-	530	40
**6**	400	-	-	568	25
**7**	450	-	-	542	35
**8**	400	525	40	525	42
**9**	390	595	12	595	10
**10**	550	680	8.5	680	7.5

*3. Steady optical spectra and lifetime of **3-7** in solid state*

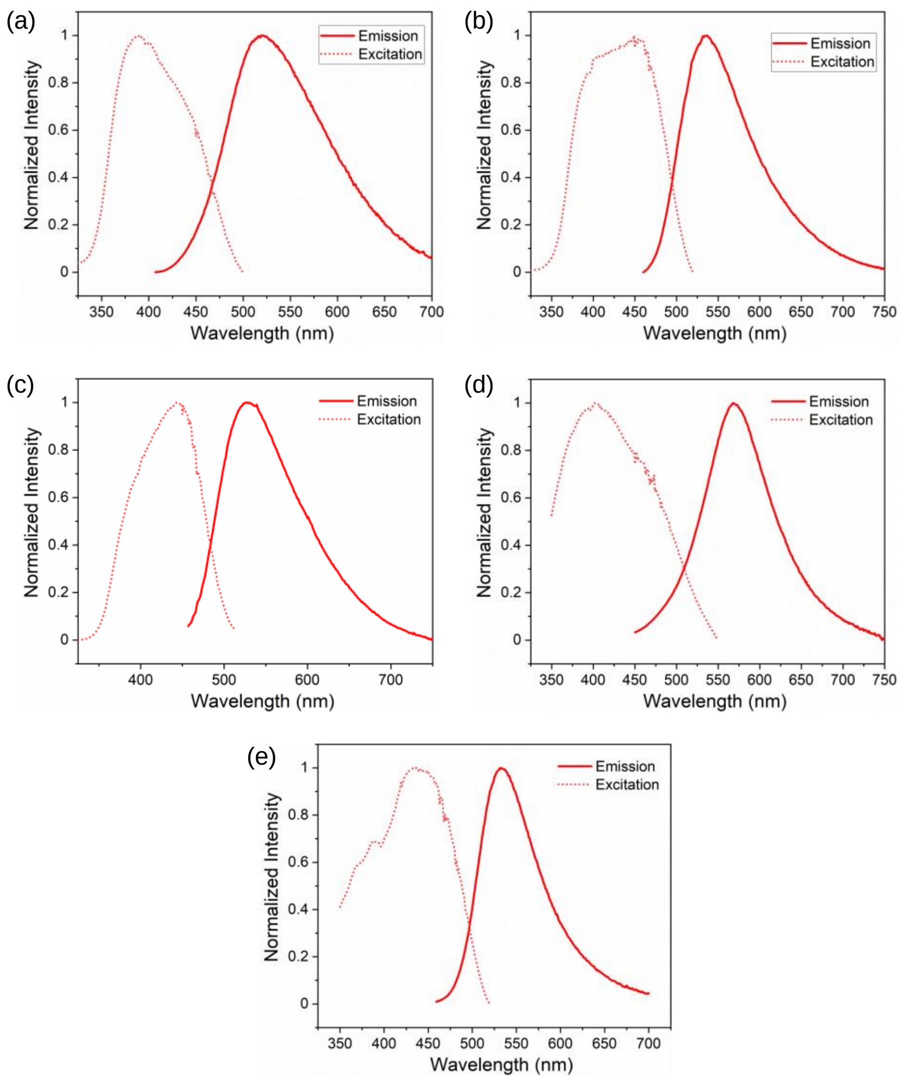

Figure S1. Excitation and Emission spectra of compound **3** - **7** in solid state after rubbed.

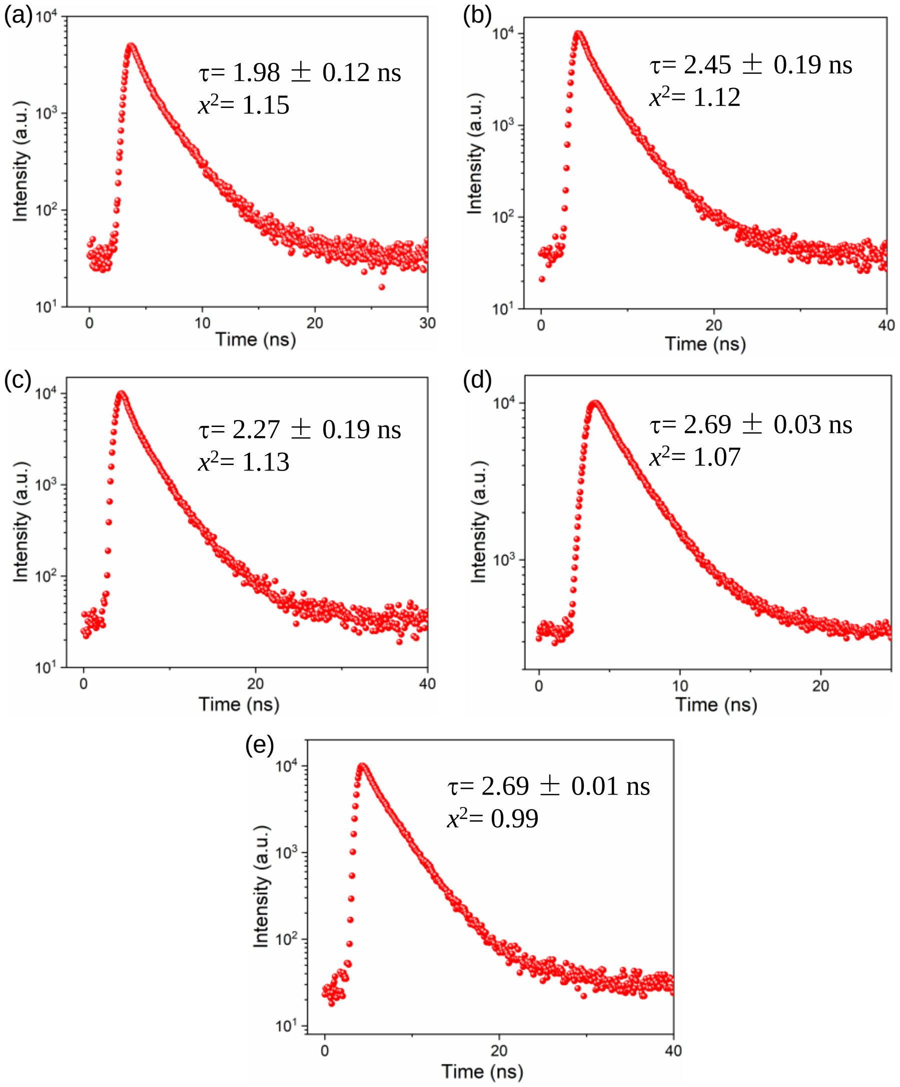

Figure S2. Lifetime decay of compound **3 - 7** in solid state after rubbed.

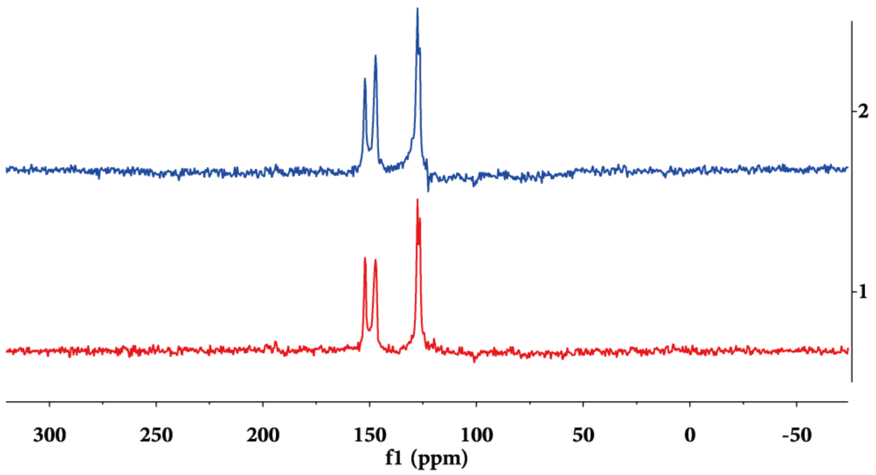

Figure S3. A full solid state ^{13}C NMR spectra of compound **3** before (blue) and after rubbing (red).

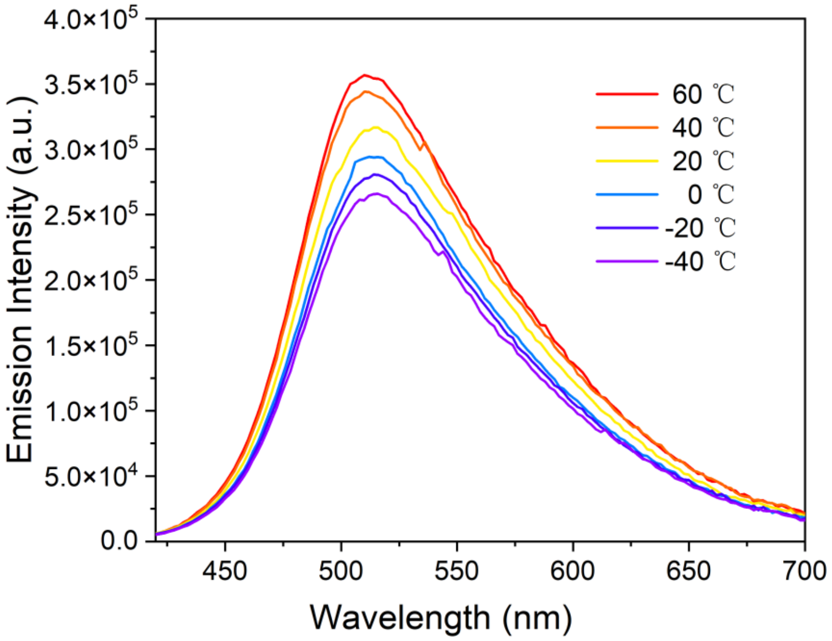

Figure S4. Temperature-dependent emission spectra of **3** in the solid state after rubbing.

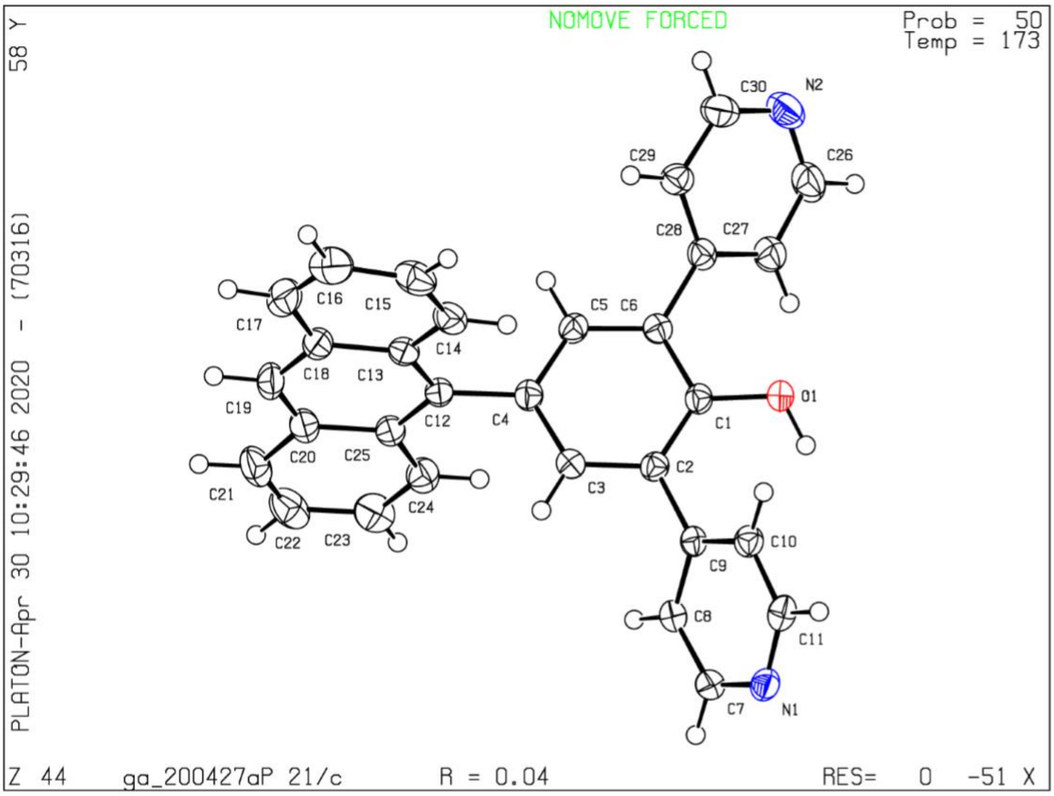

Figure S5. Single crystal structure of compound **7**.

Table S2 Crystal data and refinement details for **7**

Compound	**7**
CCDC Number	2034503
Empirical formula	C30 H20 N2 O
Formula weight	424.49
Temperature	173 K
Wavelength	0.71073 A
Crystal system, space group	Monoclinic, P 21/c
Unit cell dimensions	a = 12.9765(5) Å α = 90 º
	b = 6.5928(3) Å β= 103.5340(10)º
	c = 25.3671(10) Å γ = 90 º
Volume	2109.93(15) A^3
Z, Calculated density	4, 1.336 Mg/m^3
Absorption coefficient	0.081 mm^-1
F(000)	888.0
Crystal size	0.10 x 0.05 x 0.045 mm^3
Theta range for data collection	1.61 to 27.11º
Limiting indices	-16<=h<=16, -8<=k<=8, -30<=l<=32
Reflections collected / unique	32630 / 4671 [R(int) = 0.0627]
Completeness to theta = 27.11	99.8 %
Absorption correction	None
Max. and min. transmission	0.996 and 0.995
Refinement method	Full-matrix least-squares on F^2
Data / restraints / parameters	4671 / 0 / 298
Goodness-of-fit on F^2	2.934
Final R indices [I>2sigma(I)]	R1 = 0.0413, wR2 = 0.1046
R indices (all data)	R1 = 0.0477, wR2 = 0.1205
Largest diff. peak and hole	0.331 and -0.201 e.A^-3

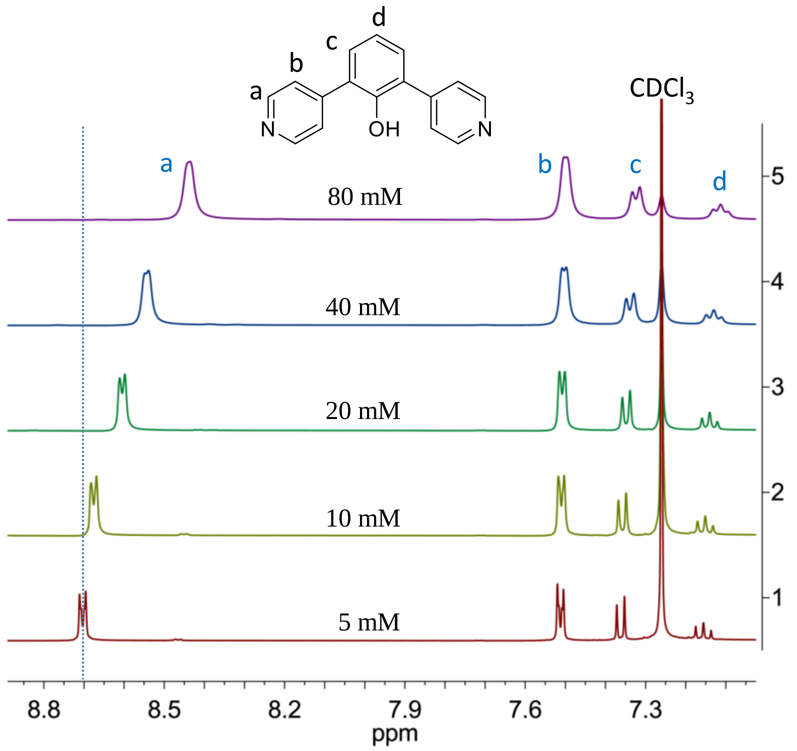

Figure S6. The concentration-dependent ^{1}H NMR of **3**.

State	State structure	Energy
Single proton transfer free state	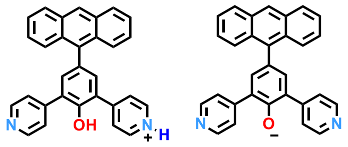	**95 kcal/mol**
Double proton transfer free state	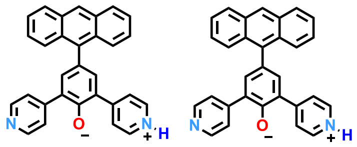	**36 kcal/mol**
Ground free state	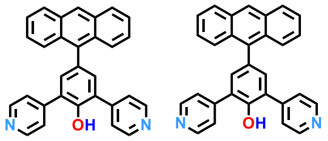	**20 kcal/mol**
Double proton trasfer	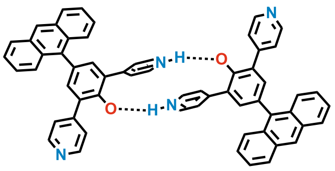	**14 kcal/mol**
Double proton trasfer Transition State	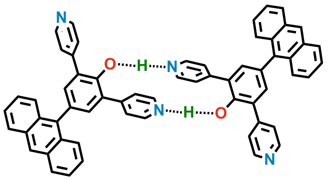	**15 kcal/mol (Transition State)**
Single proton trasfer	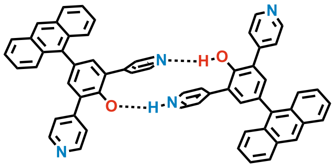	**10 kcal/mol**
Single proton trasfer Transition State	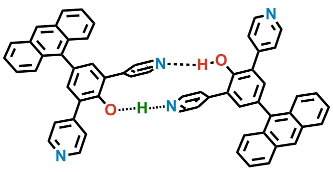	**11 kcal/mol (Transition State)**
Ground State	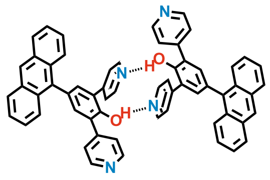	**0 kcal/mol**

Figure S7. The calculation result of the energy level at different state.

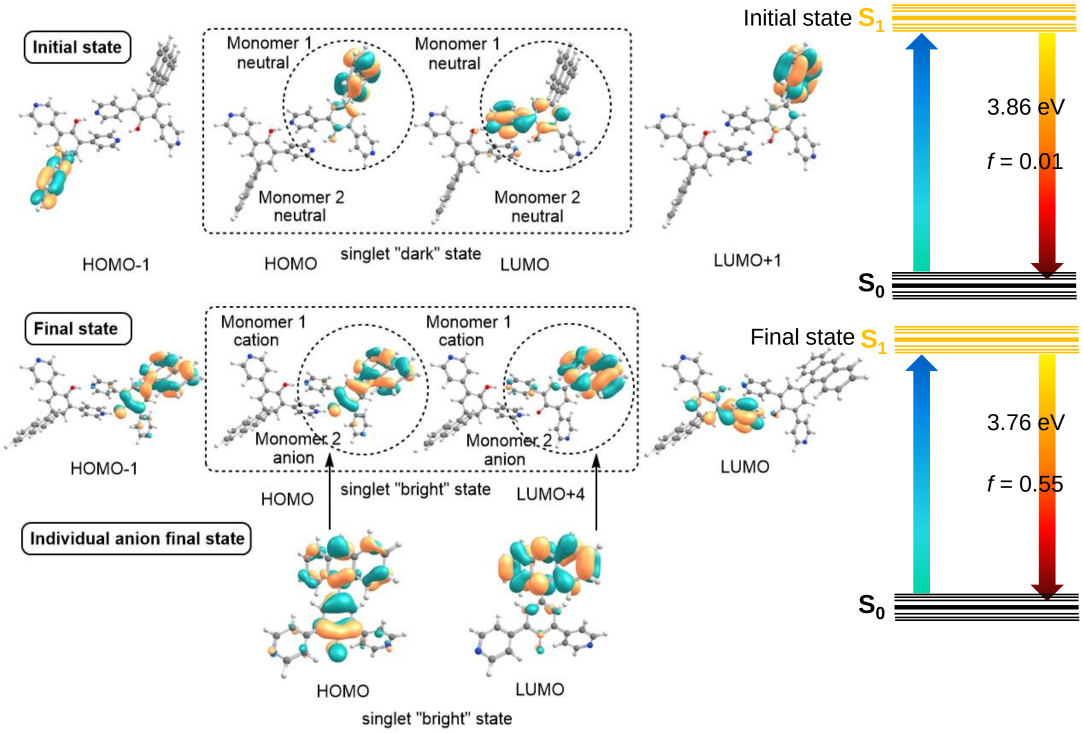

Figure S8. The shape of frontier molecular orbitals for the initial and final (rubbing-induced) state of dimeric compound **7**. The spectrally active orbitals for individual anionic “bright” species are also presented.

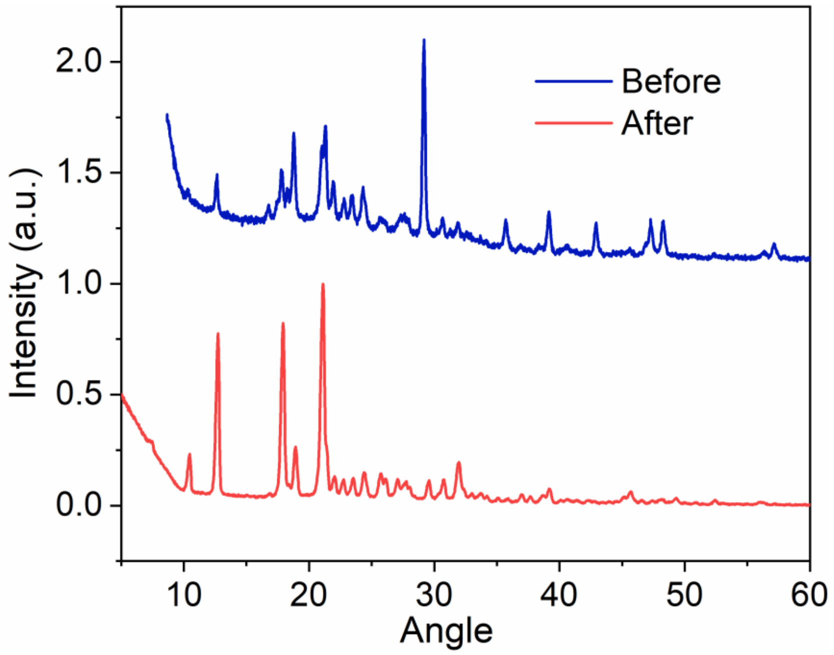

Figure S9. The powder XRD spectrum of **3** before and after rubbing.

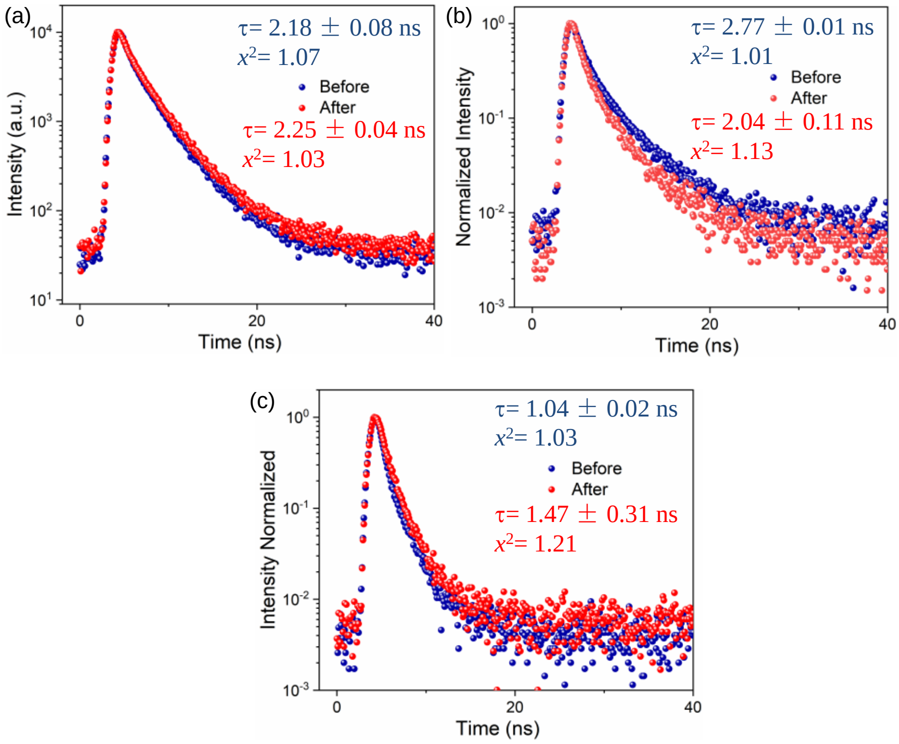

Figure S10. Lifetime decay of compound **8 - 10** in solid state after rubbing.

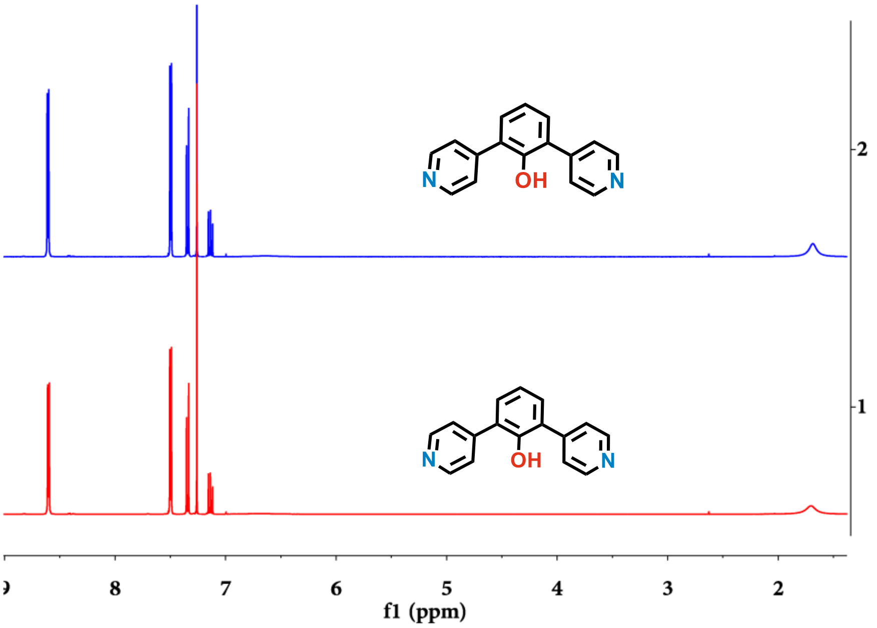

Figure S11. The ^{1}H NMR spectrum of compound **3** in $CDCl_3$ before (blue) and after (red) rubbing.

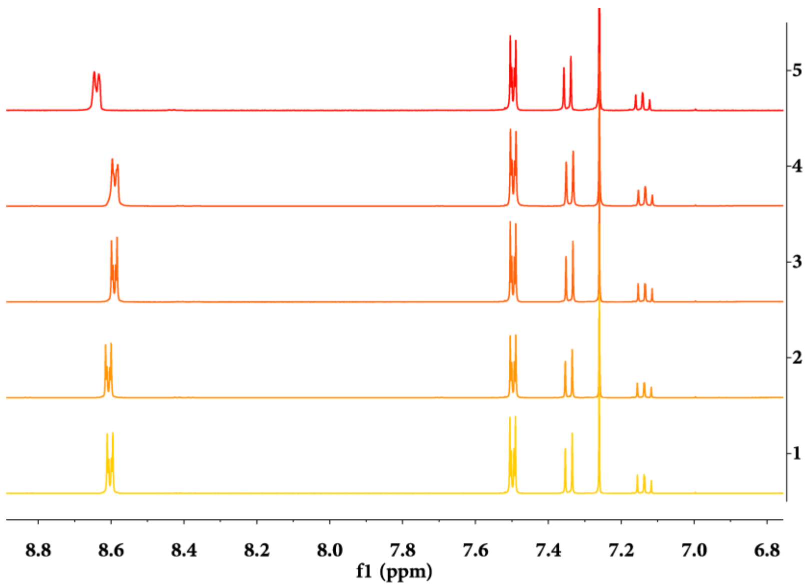

Figure S12. The ^{1}H NMR spectra of compound **3** recovered from water without further purification in 5 rounds.

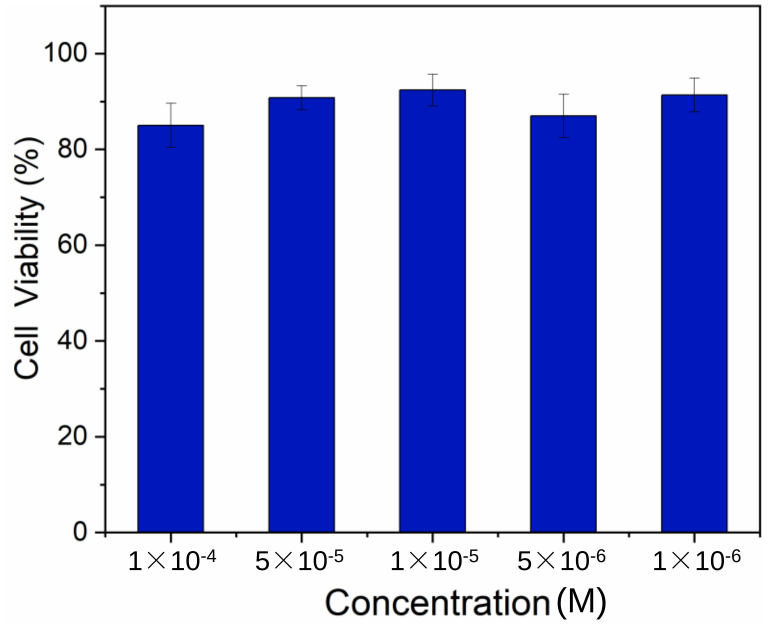

Figure S13. The 12 hour survival rate of HeLa cells.

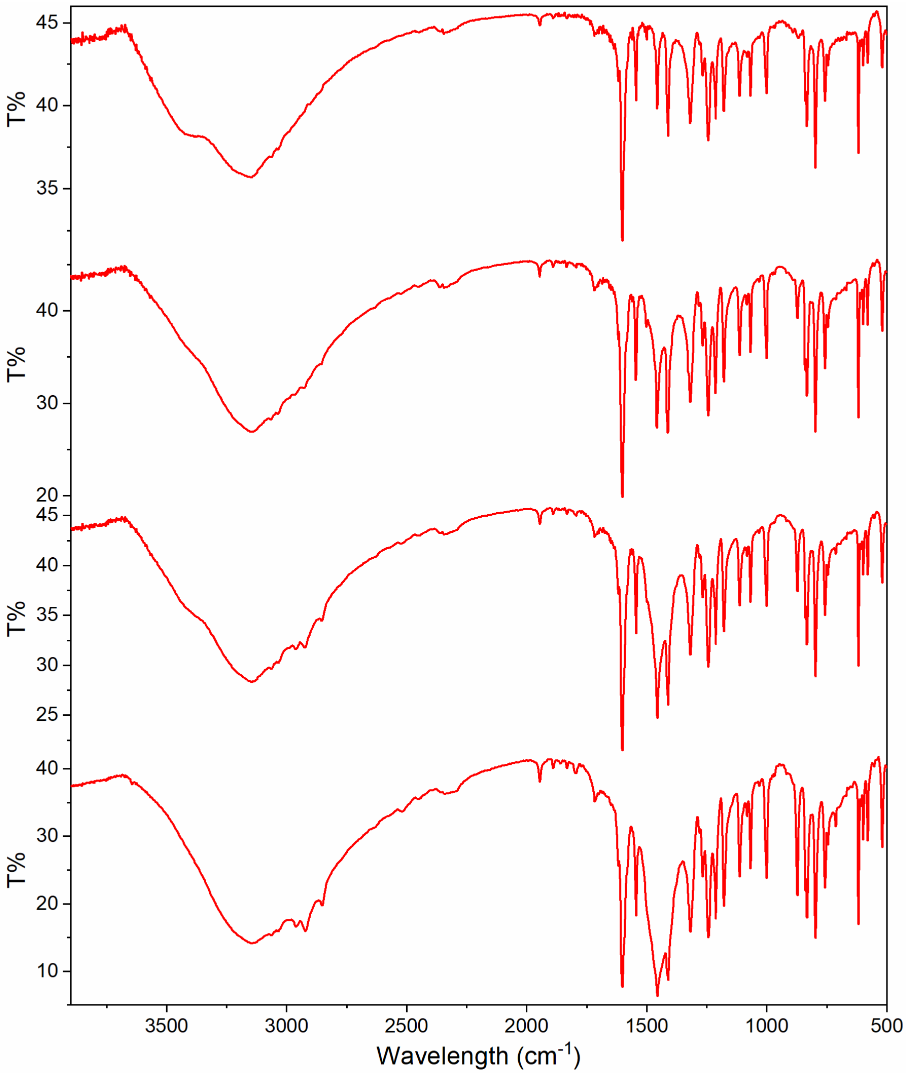

Figure S14. Full rubbing dependent IR spectra of compound **3**.

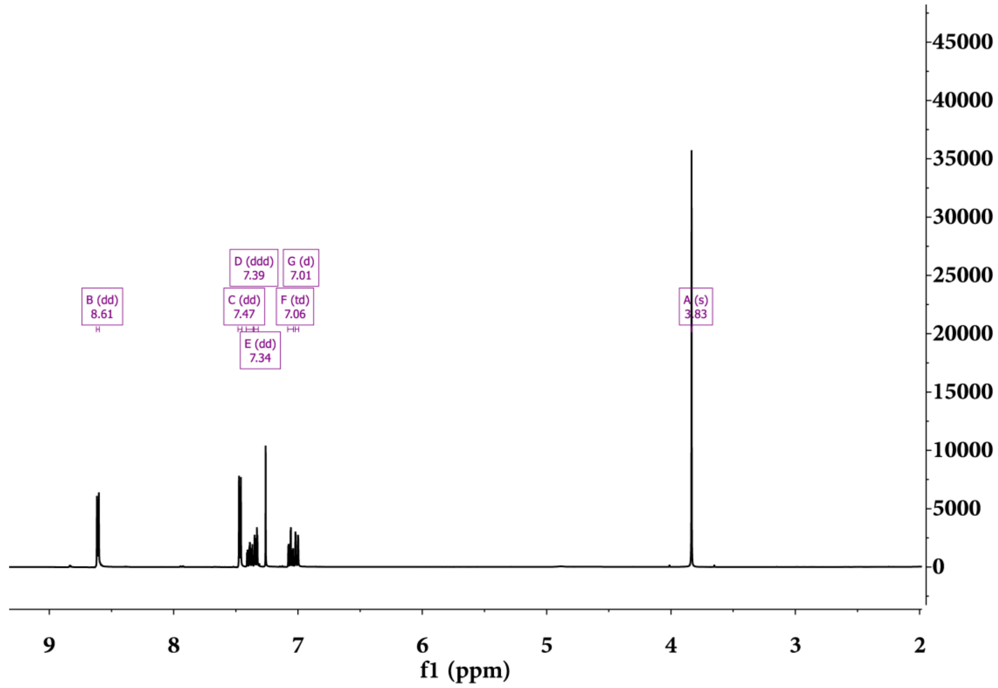

Figure S15. ^{1}H NMR spectrum of **1a**.

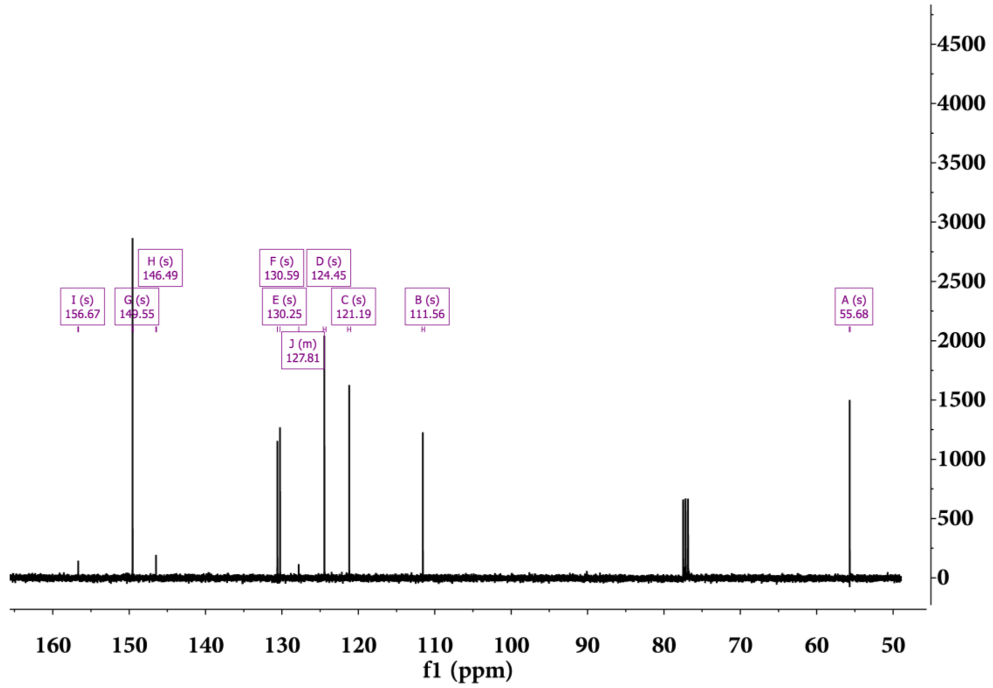

Figure S16. ^{13}C NMR spectrum of **1a**.

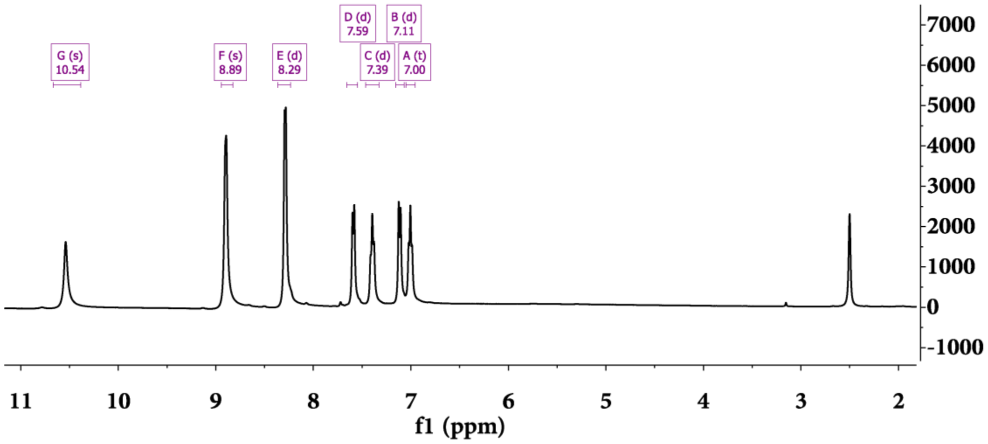

Figure S17. ^{1}H NMR spectrum of **1**.

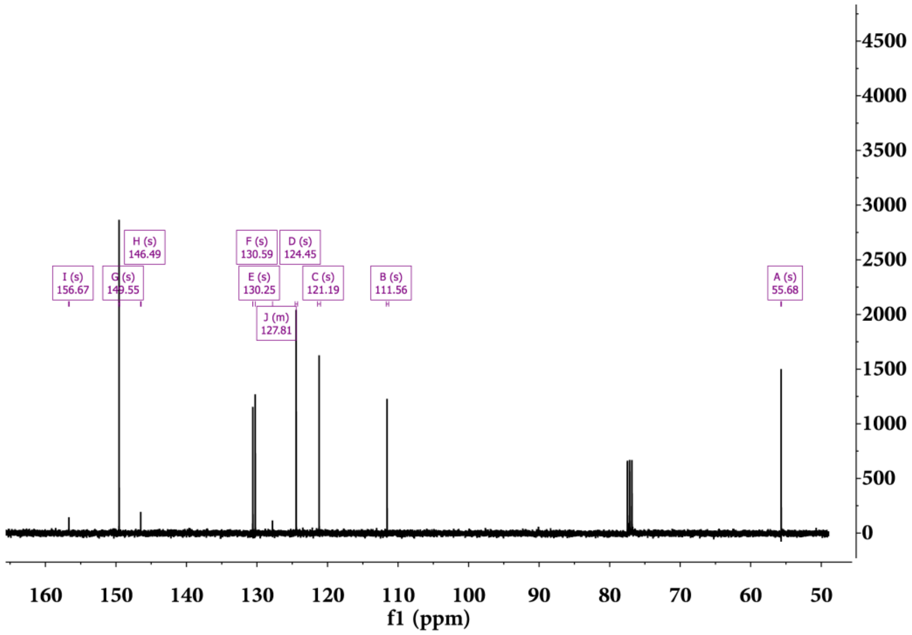

Figure S18. ^{13}C NMR spectrum of **1**.

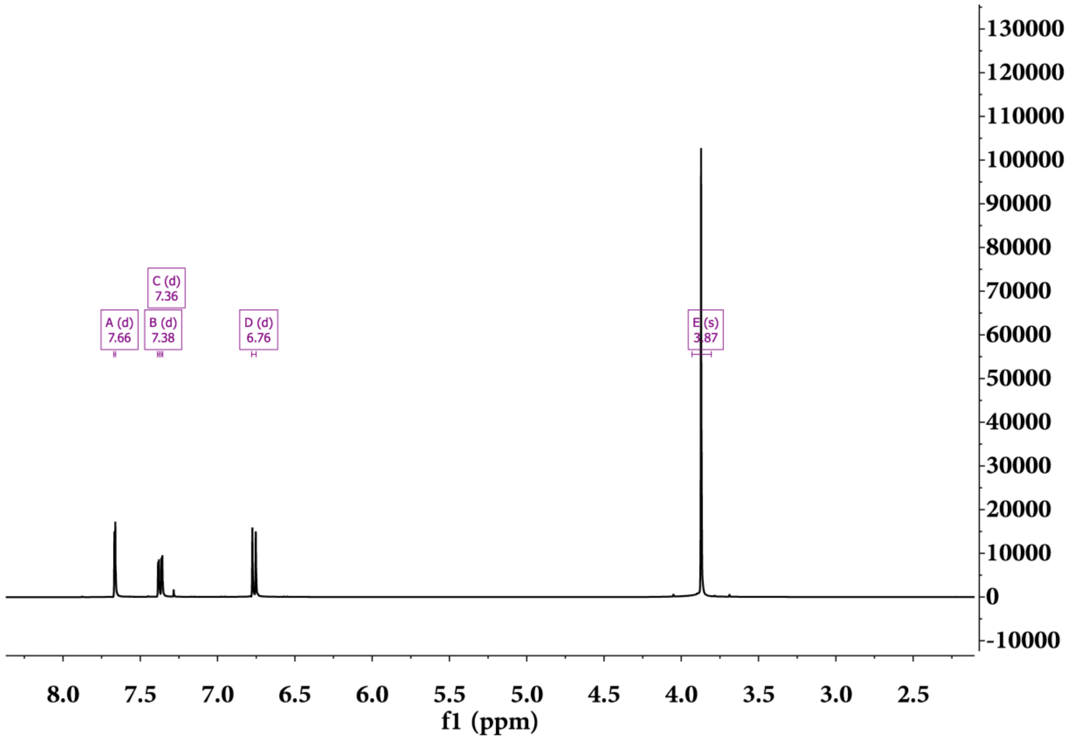

Figure S19. ^{1}H NMR spectrum of **2a**.

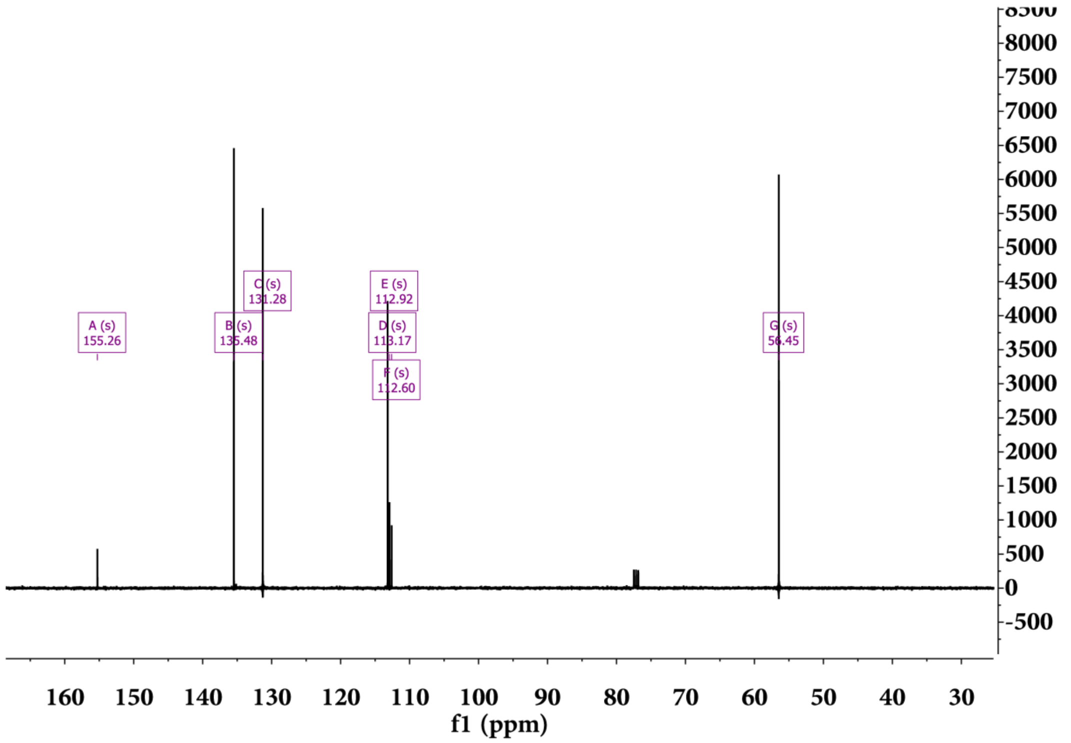

Figure S20. ^{13}C NMR spectrum of **2a**.

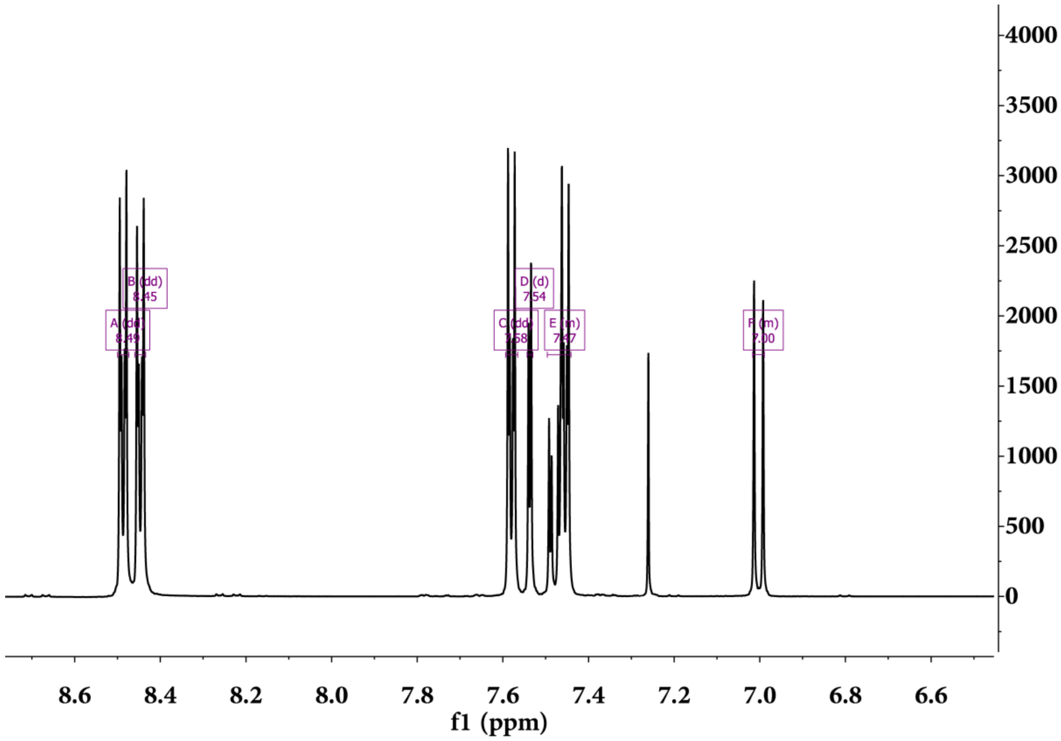

Figure S21. ^{1}H NMR spectrum of **2**.

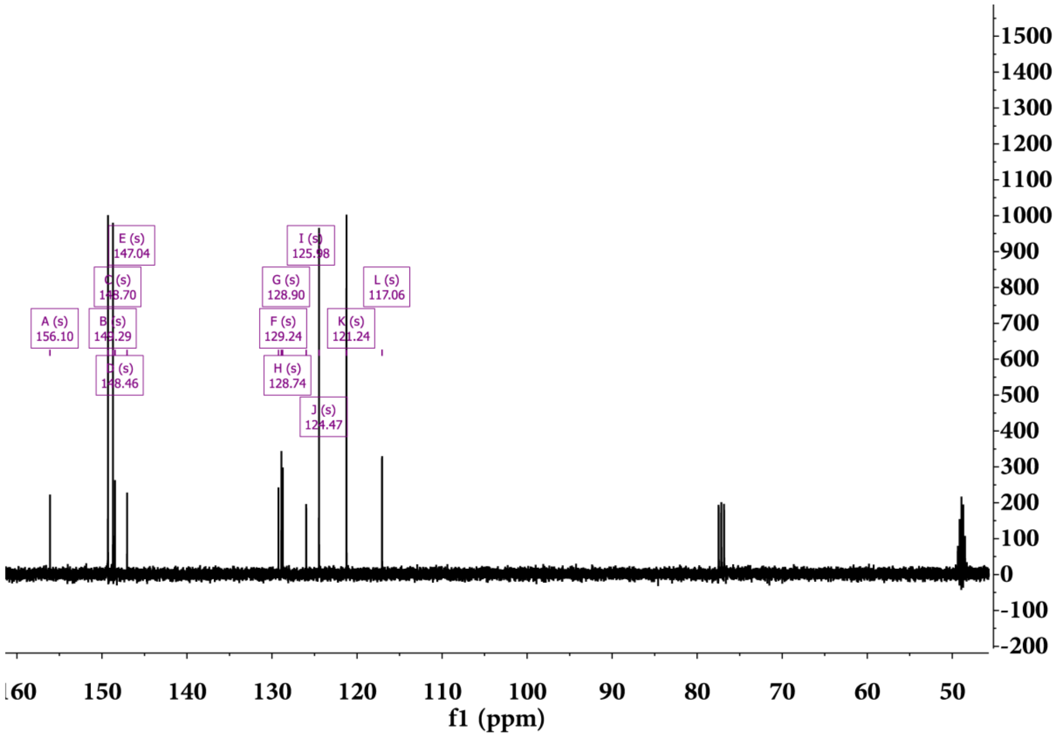

Figure S22. ^{13}C NMR spectrum of **2**.

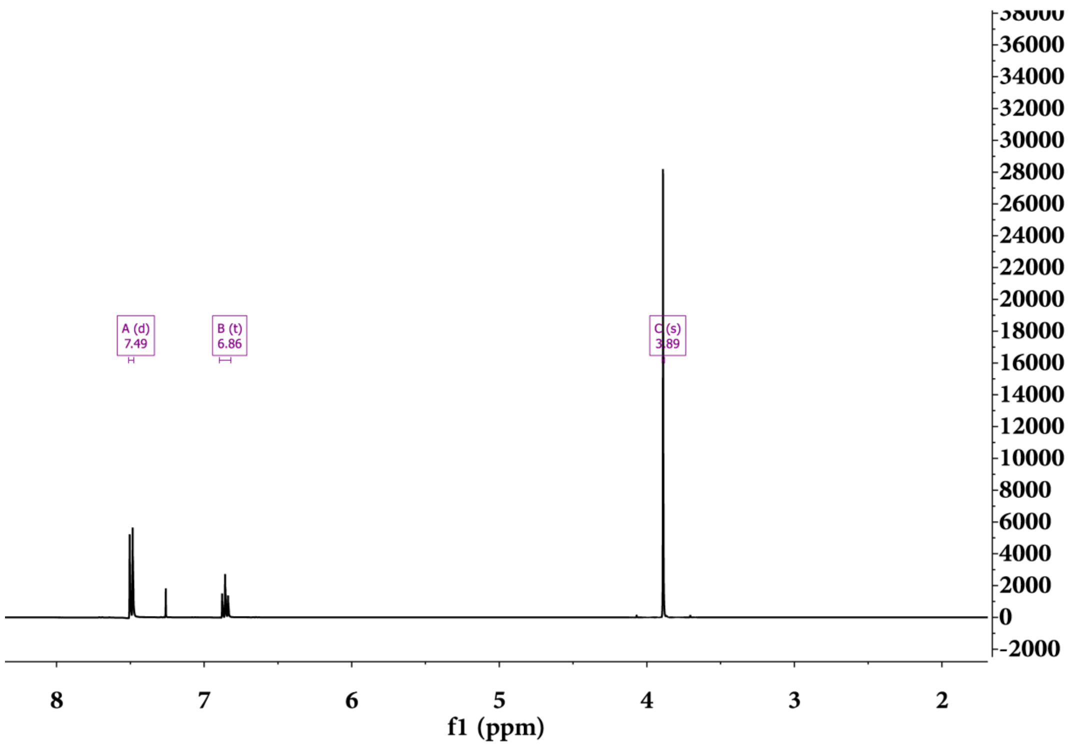

Figure S23. ^{1}H NMR spectrum of **3a**.

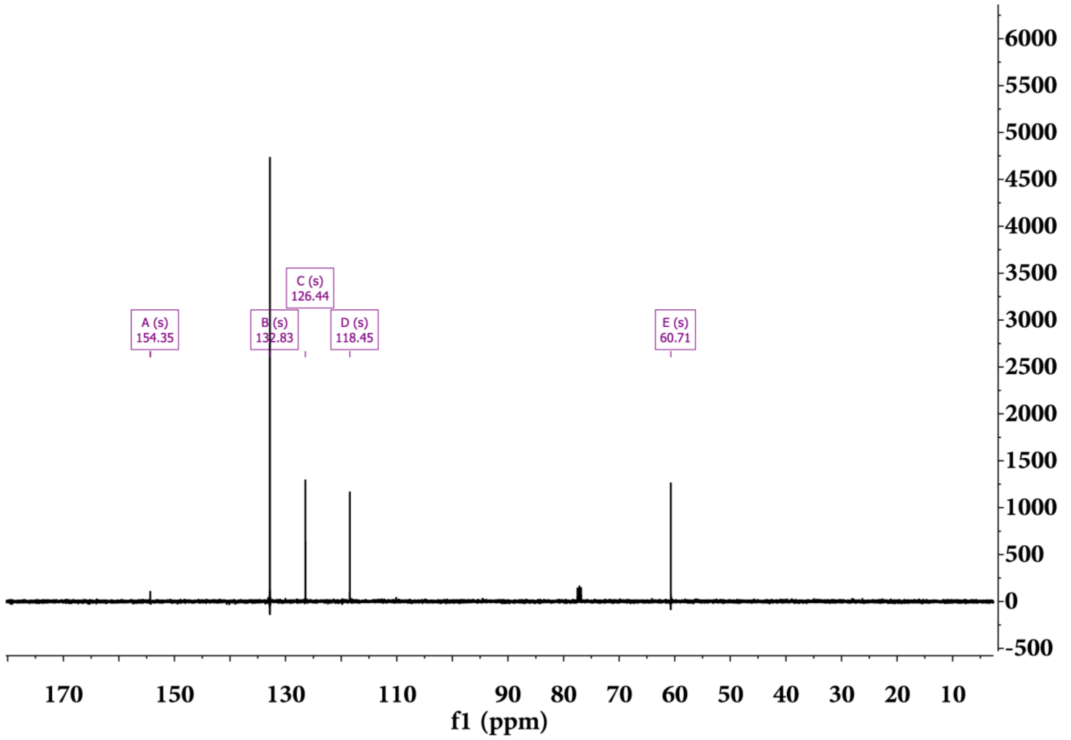

Figure S24. ^{13}C NMR spectrum of **3a**.

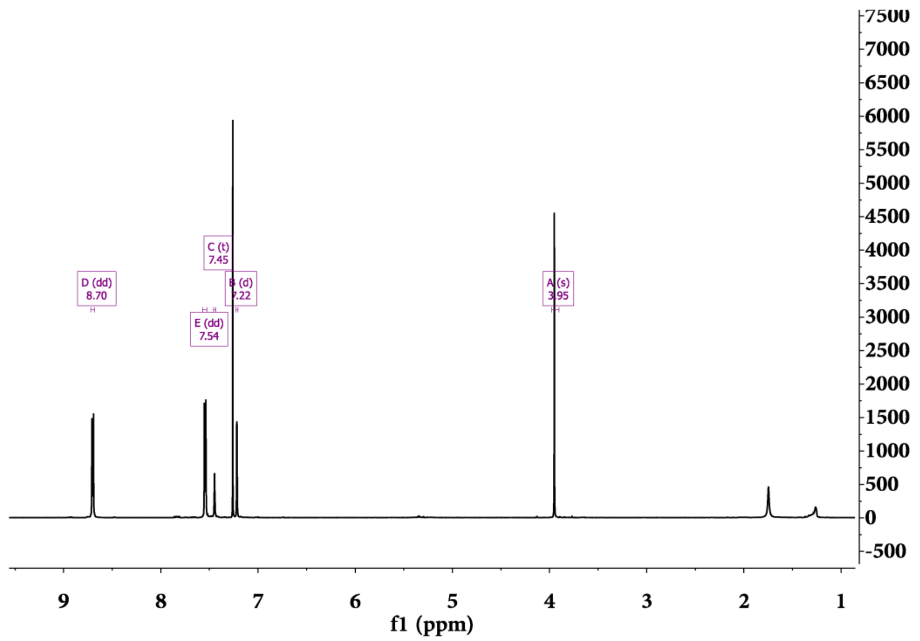

Figure S25. ^{1}H NMR spectrum of **3b**.

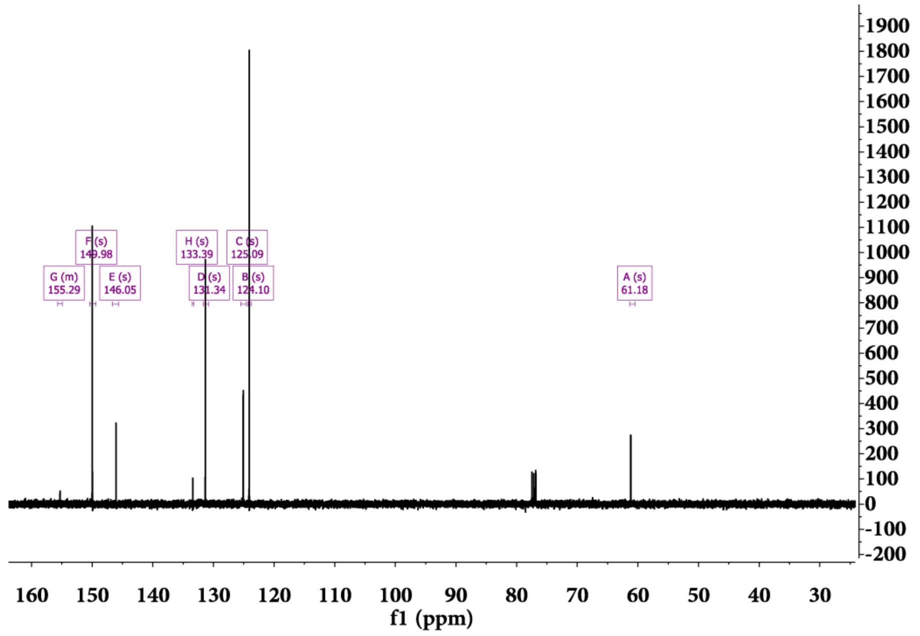

Figure S26. ^{13}C NMR spectrum of **3b**.

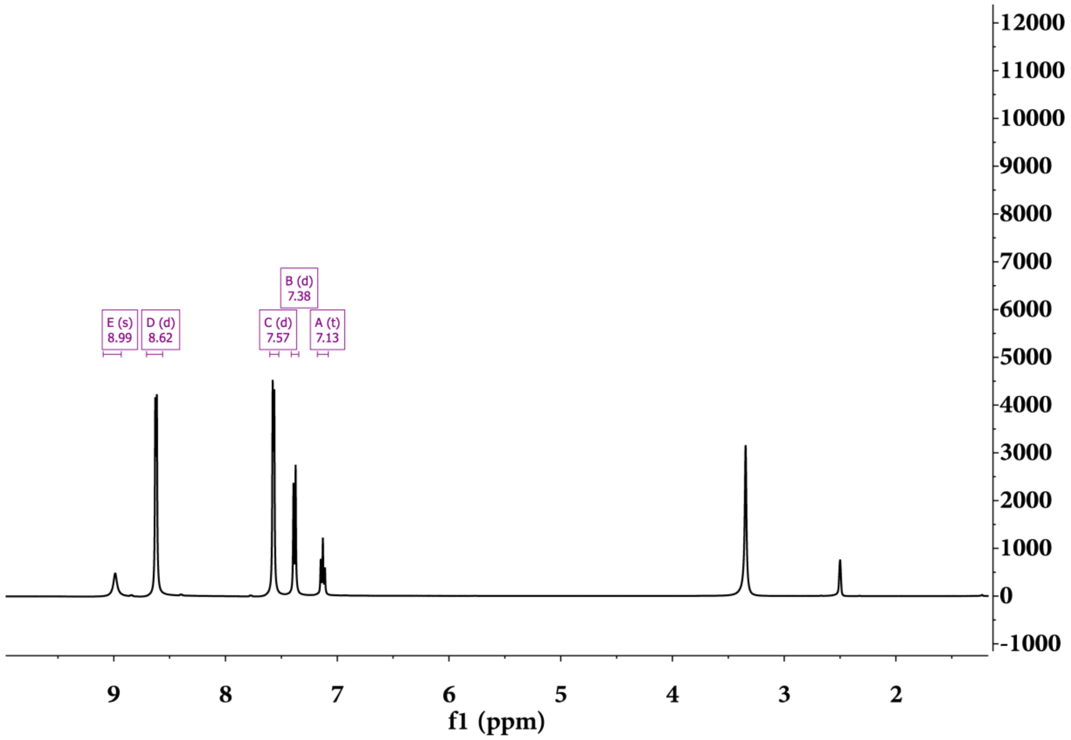

Figure S27. ^{1}H NMR spectrum of **3**.

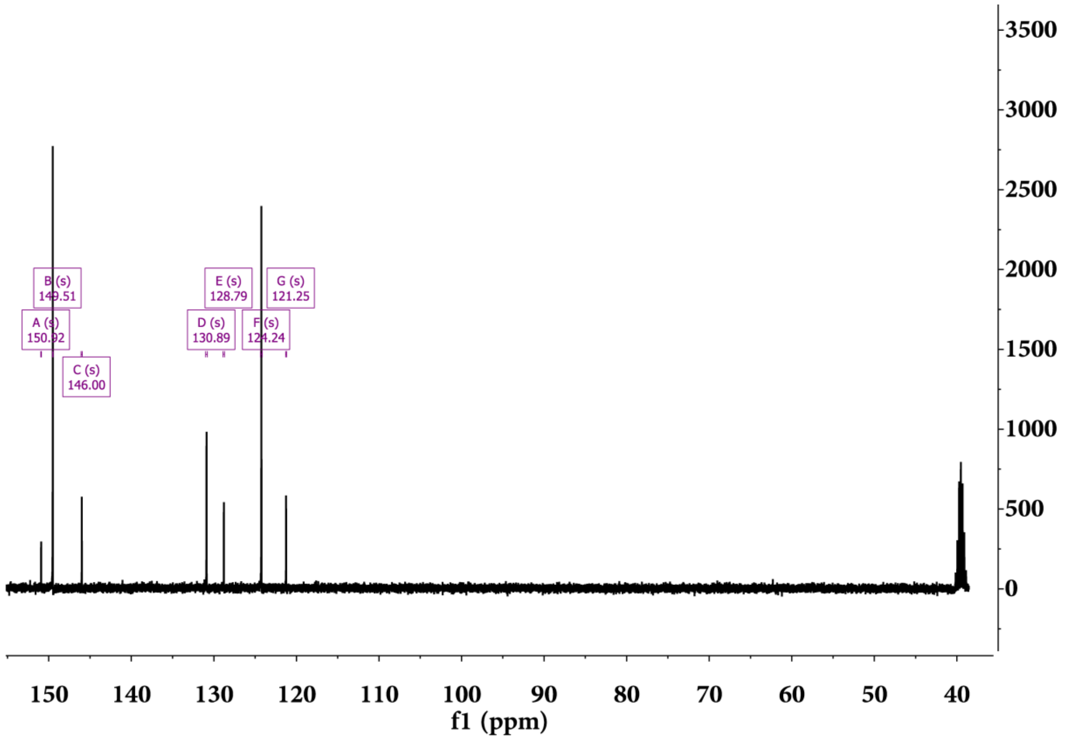

Figure S28. ^{13}C NMR spectrum of **3**.

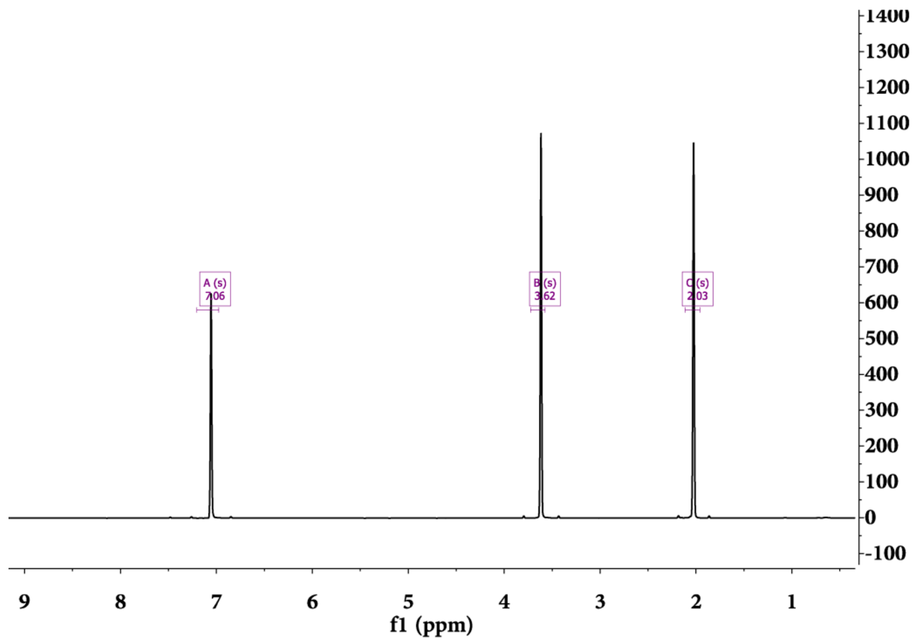

Figure S29. ^{1}H NMR spectrum of **4a**.

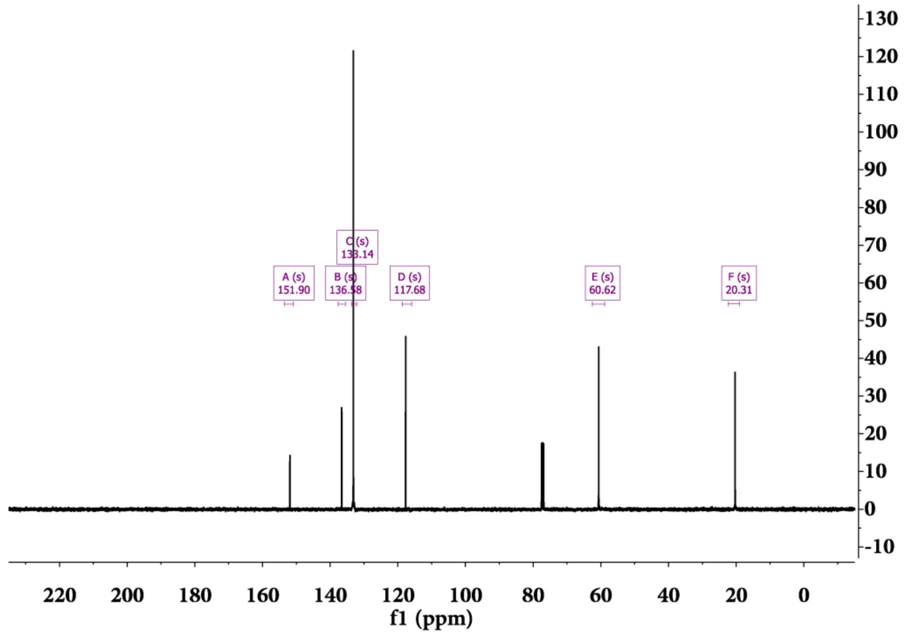

Figure S30. ^{13}C NMR spectrum of **4a**.

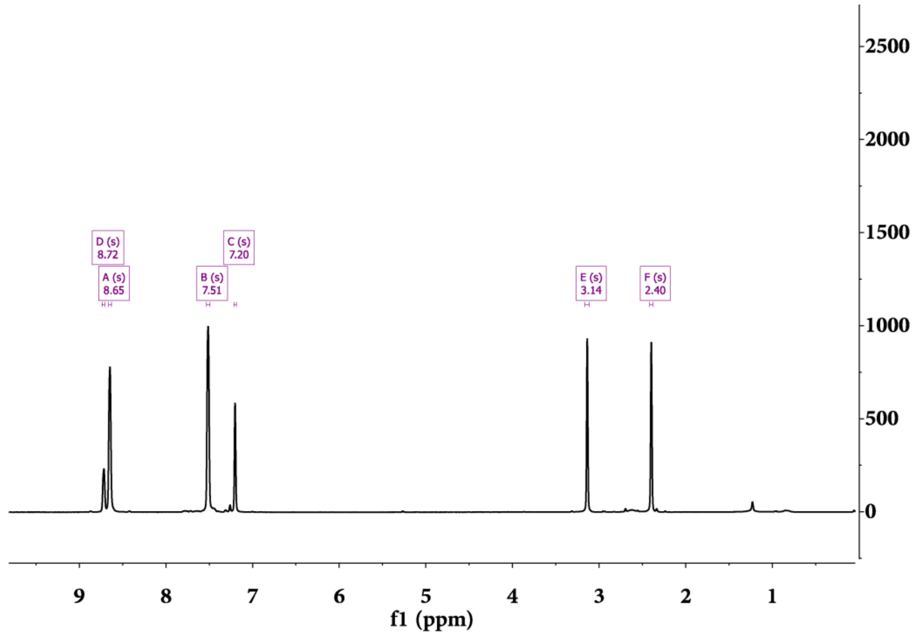

Figure S31. ^{1}H NMR spectrum of **4b**.

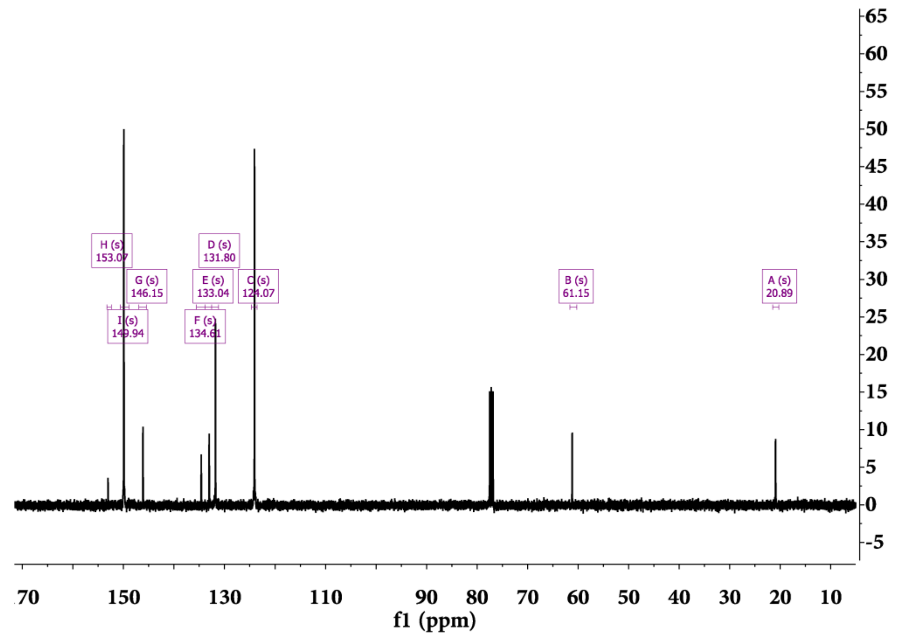

Figure S32. ^{13}C NMR spectrum of **4b**.

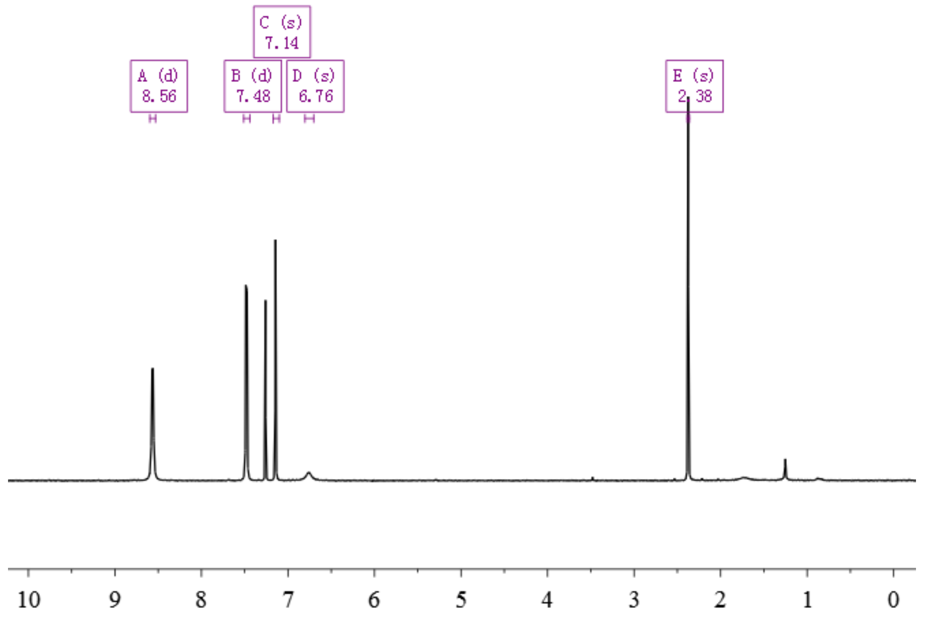

Figure S33. ^{1}H NMR spectrum of **4**.

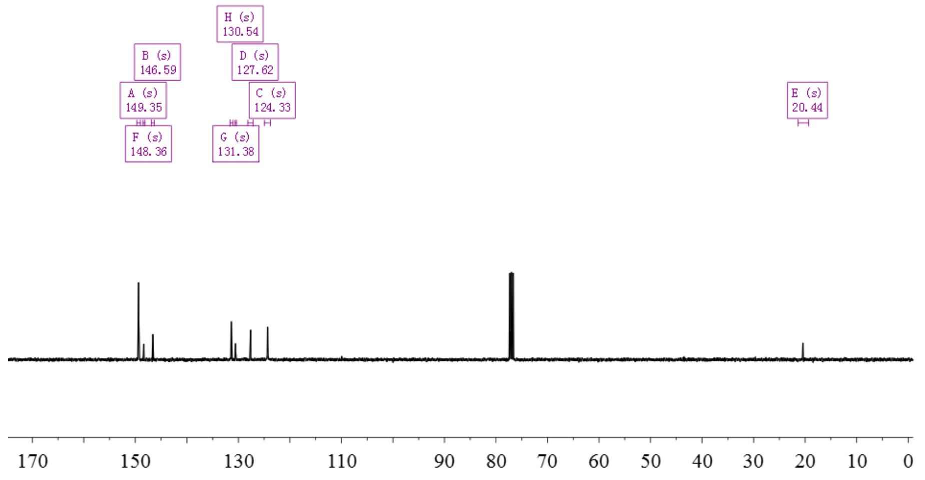

Figure S34. ^{13}C NMR spectrum of **4**.

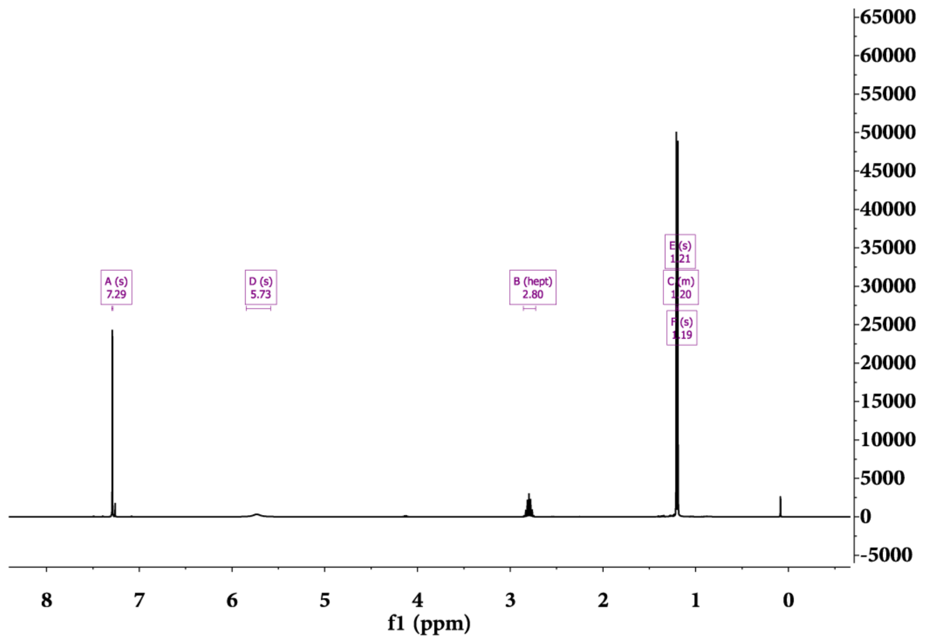

Figure S35. ^{1}H NMR spectrum of **5a**.

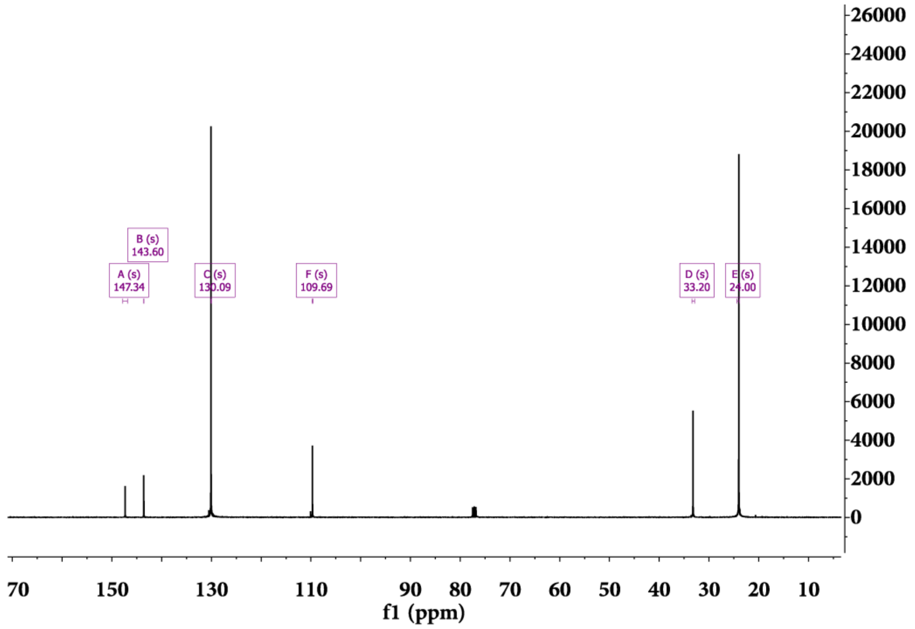

Figure S36. ^{13}C NMR spectrum of **5a**.

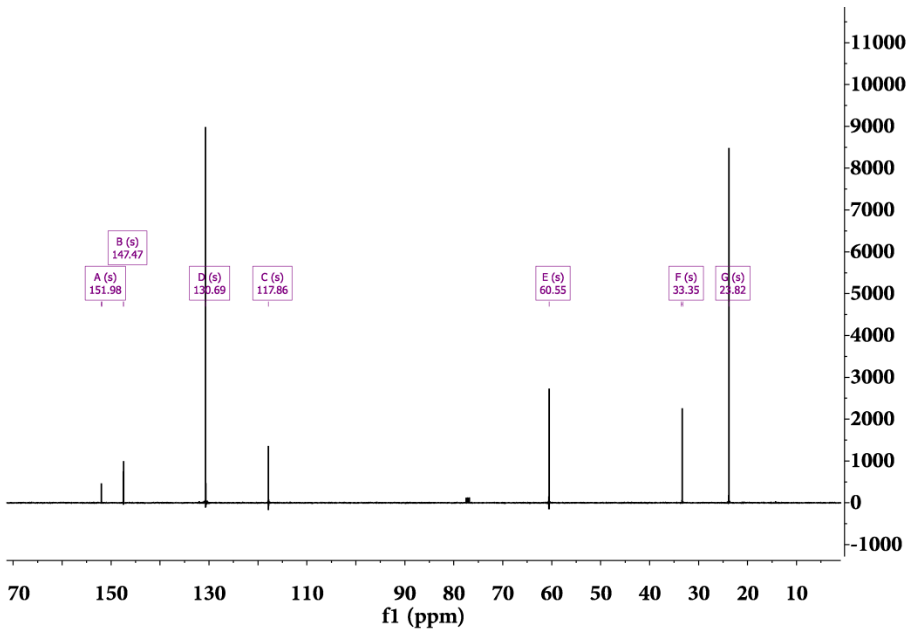

Figure S37. ^{13}C NMR spectrum of **5b**.

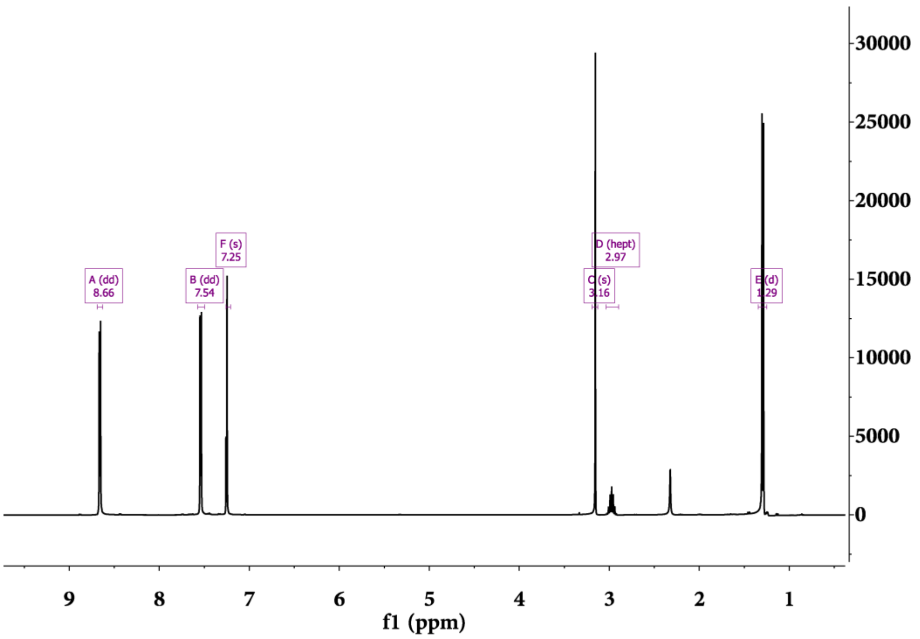

Figure S38. ^{1}H NMR spectrum of **5c**.

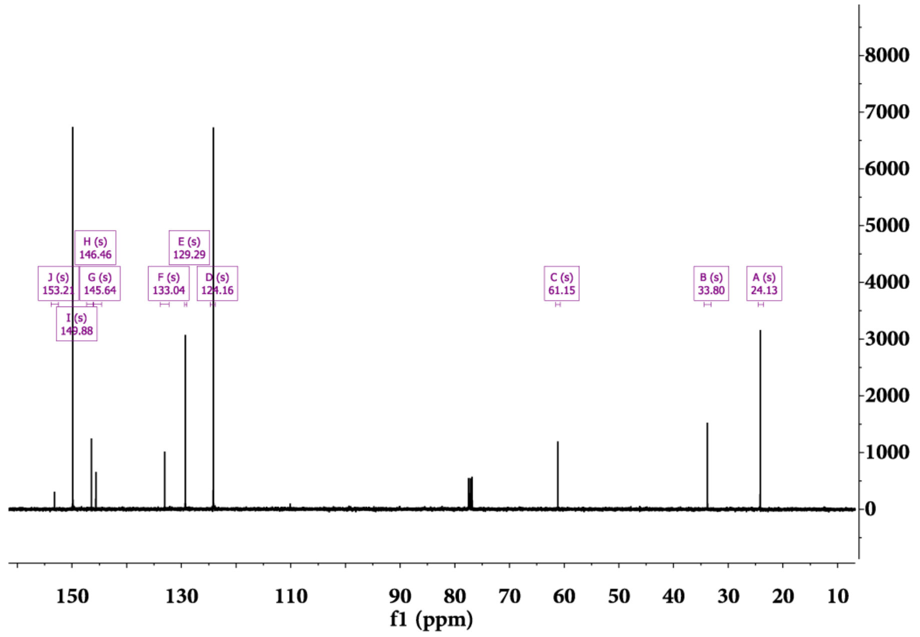

Figure S39. ^{13}C NMR spectrum of **5c**.

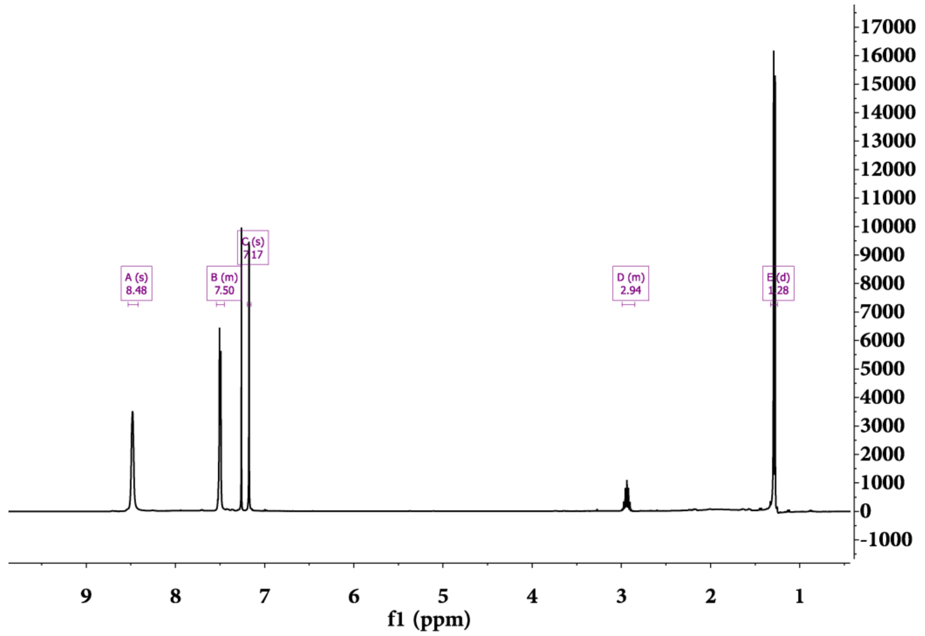

Figure S40. ^{1}H NMR spectrum of **5**.

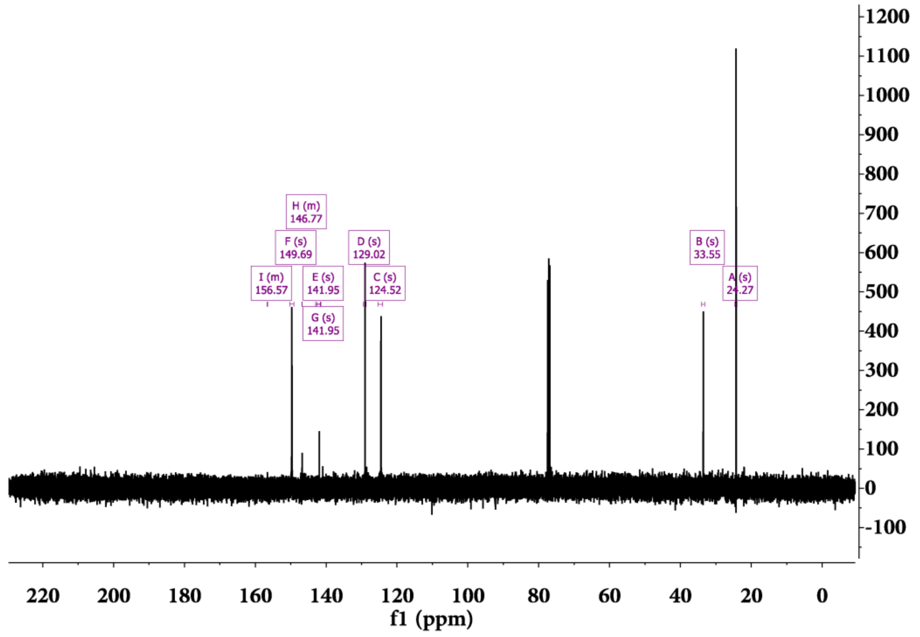

Figure S41. ^{13}C NMR spectrum of **5**.

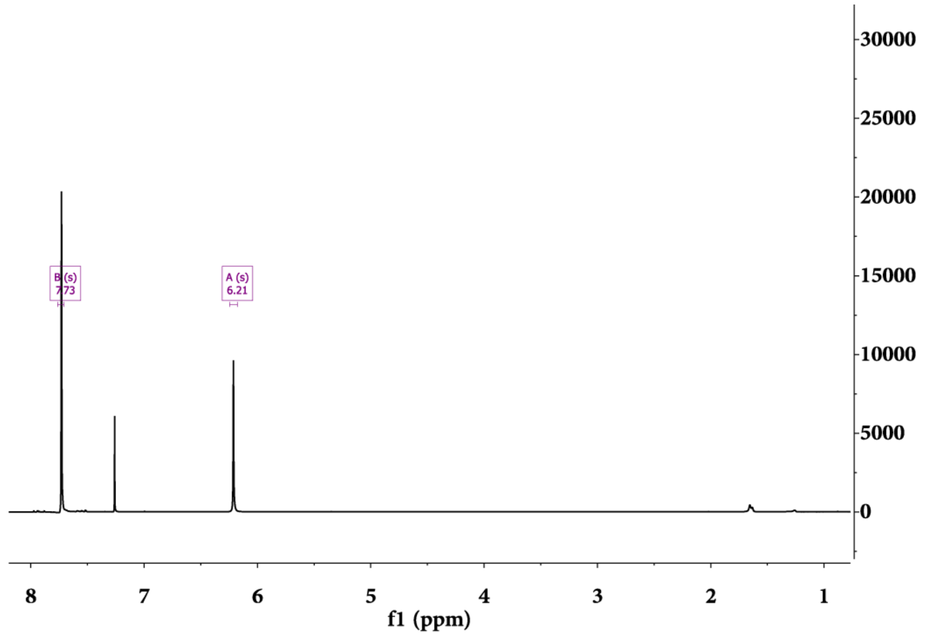

Figure S42. ^{1}H NMR spectrum of **6a**.

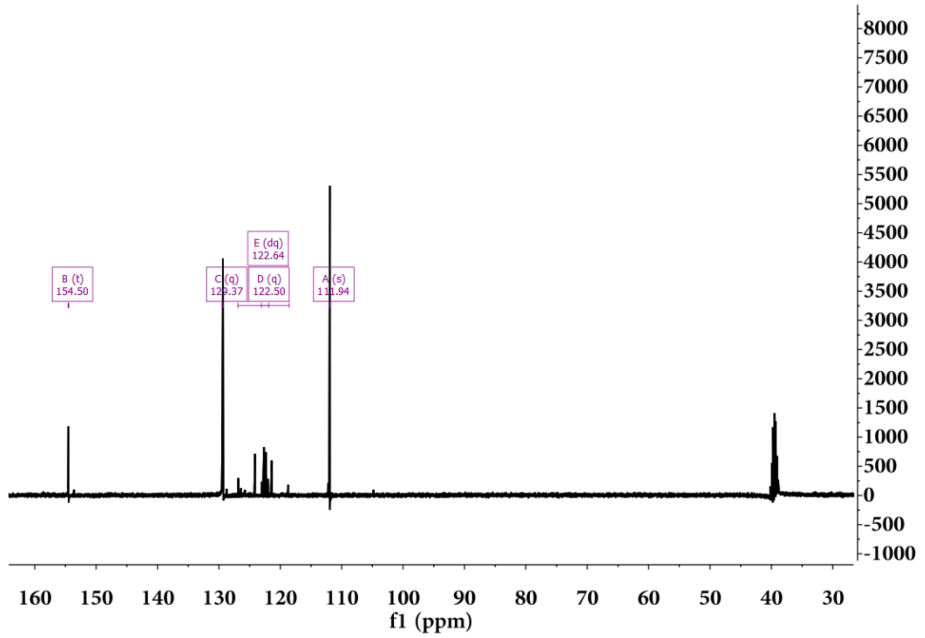

Figure S43. ^{13}C NMR spectrum of **6a**.

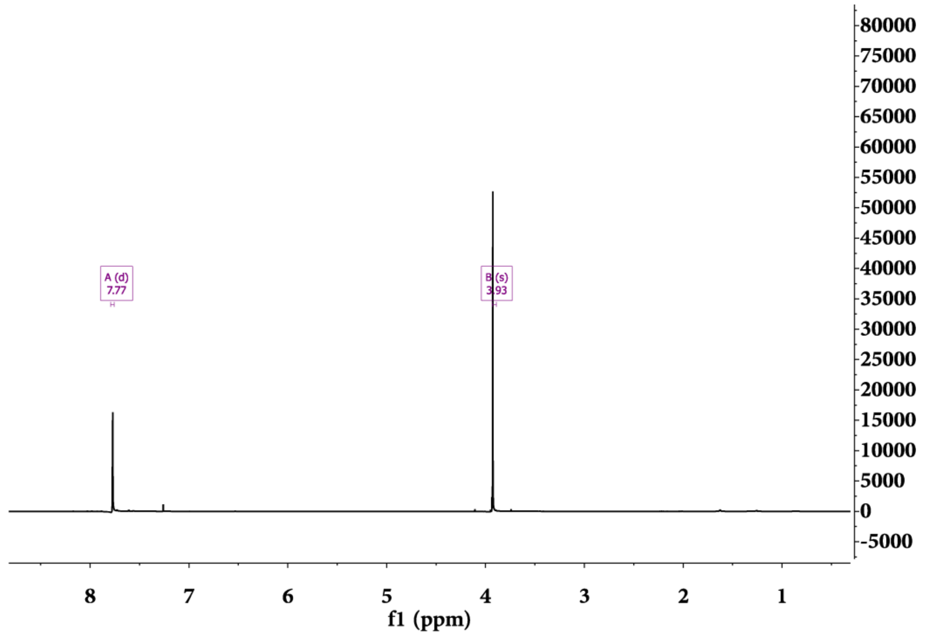

Figure S44. ^{1}H NMR spectrum of **6b**.

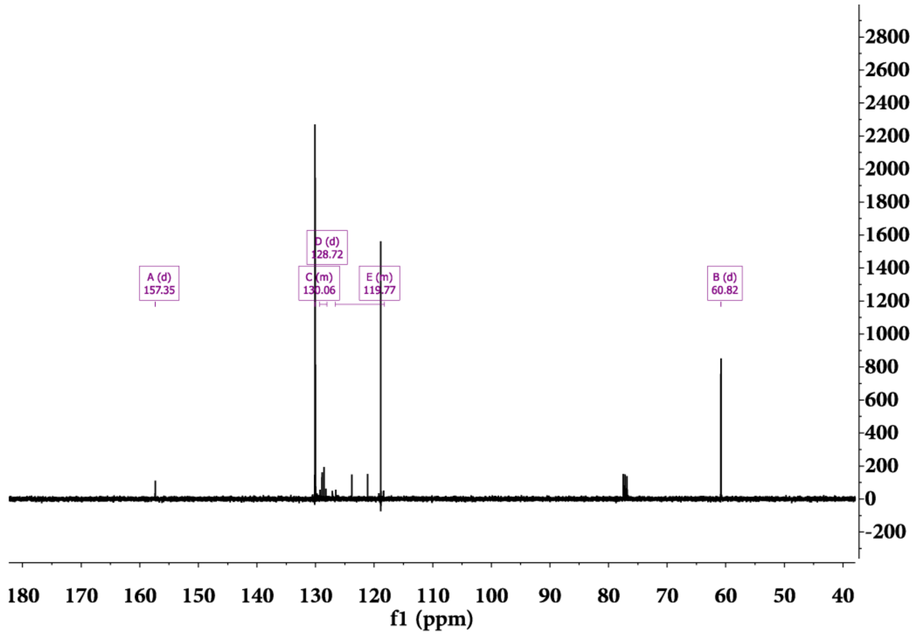

Figure S45. ^{13}C NMR spectrum of **6b**.

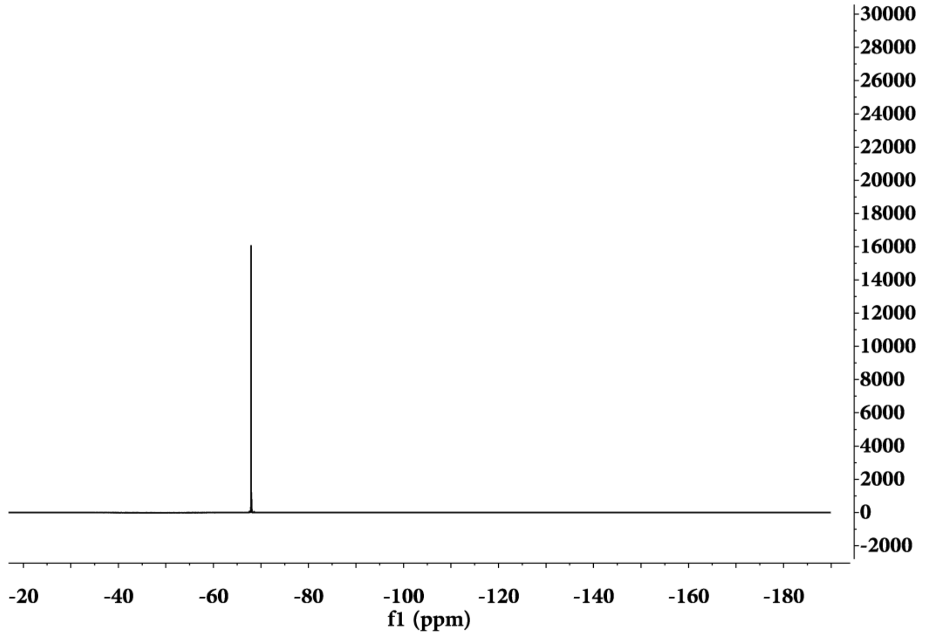

Figure S46. ^{19}F NMR spectrum of **6b**.

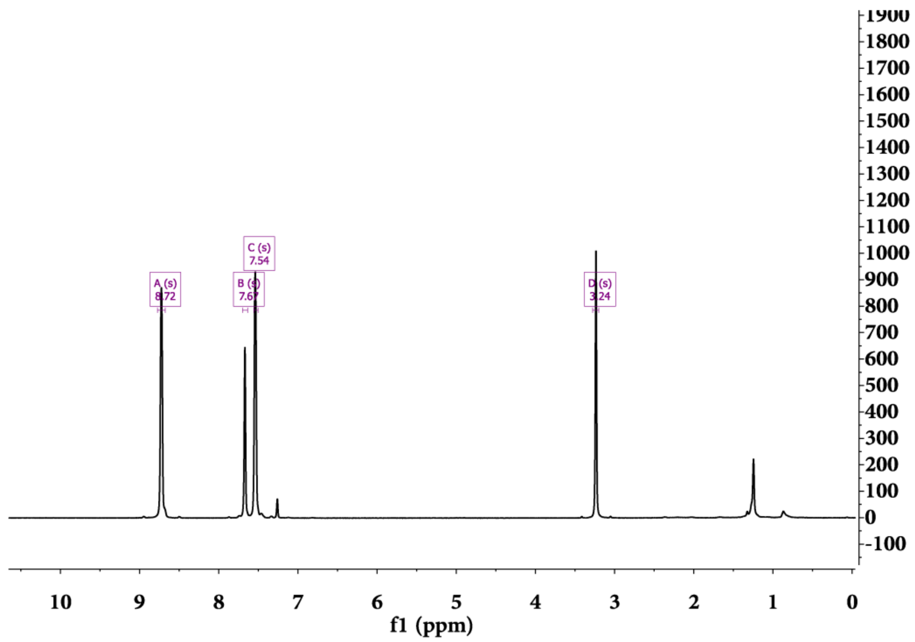

Figure S47. ^{1}H NMR spectrum of **6c**.

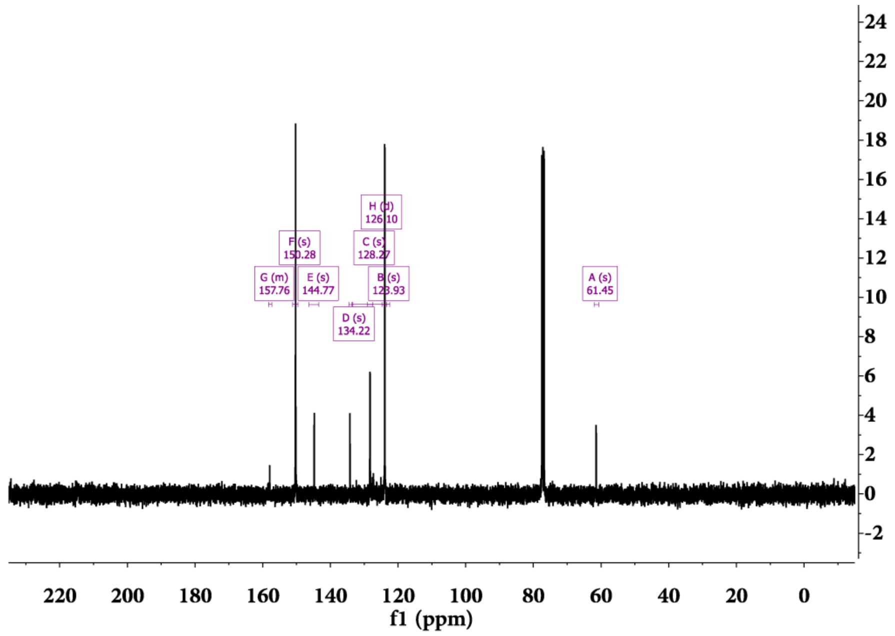

Figure S48. ^{13}C NMR spectrum of **6c**.

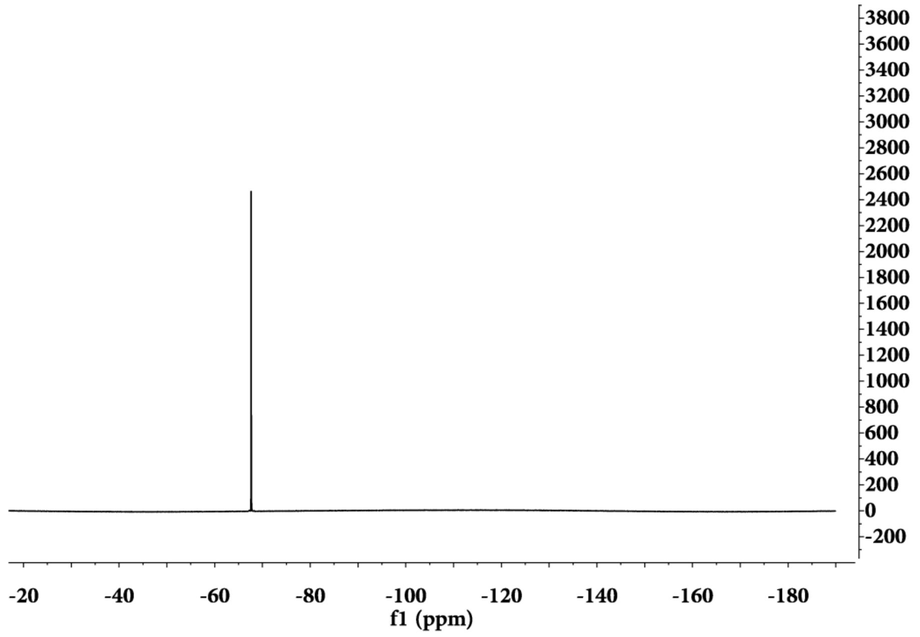

Figure S49. ${}^{19}F$ NMR spectrum of **6c**.

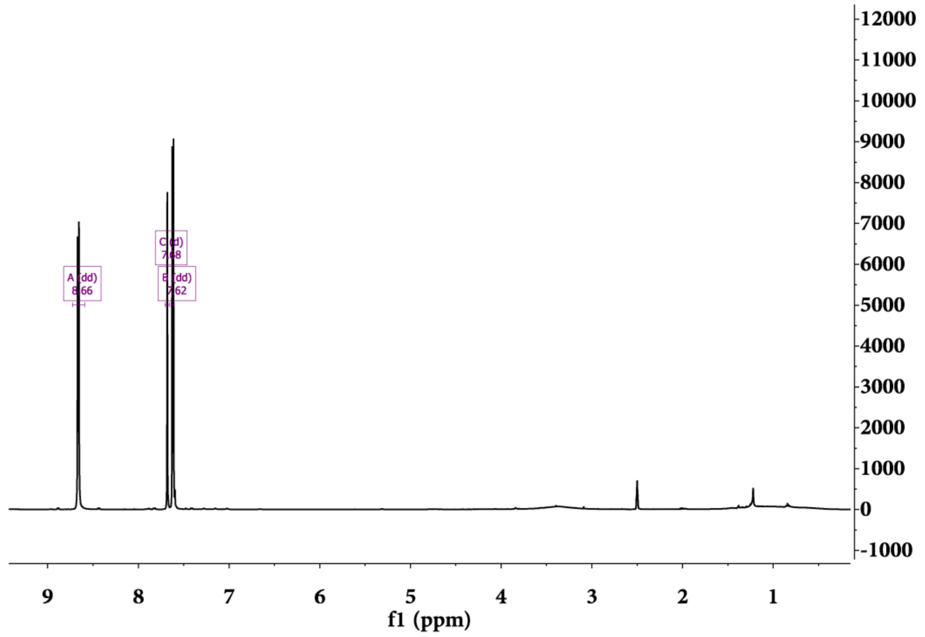

Figure S50. ${}^{1}H$ NMR spectrum of **6**.

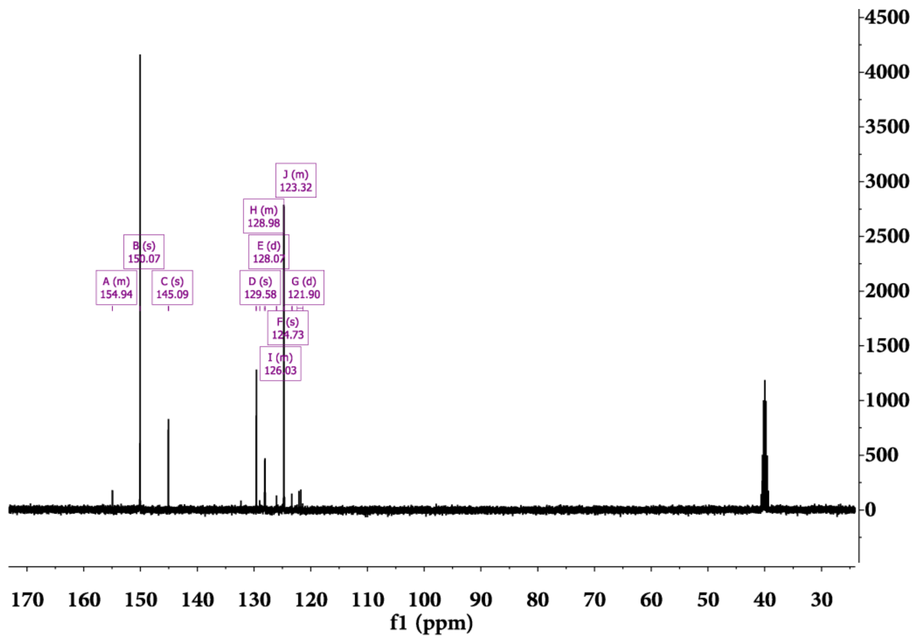

Figure S51. ^{13}C NMR spectrum of **6**.

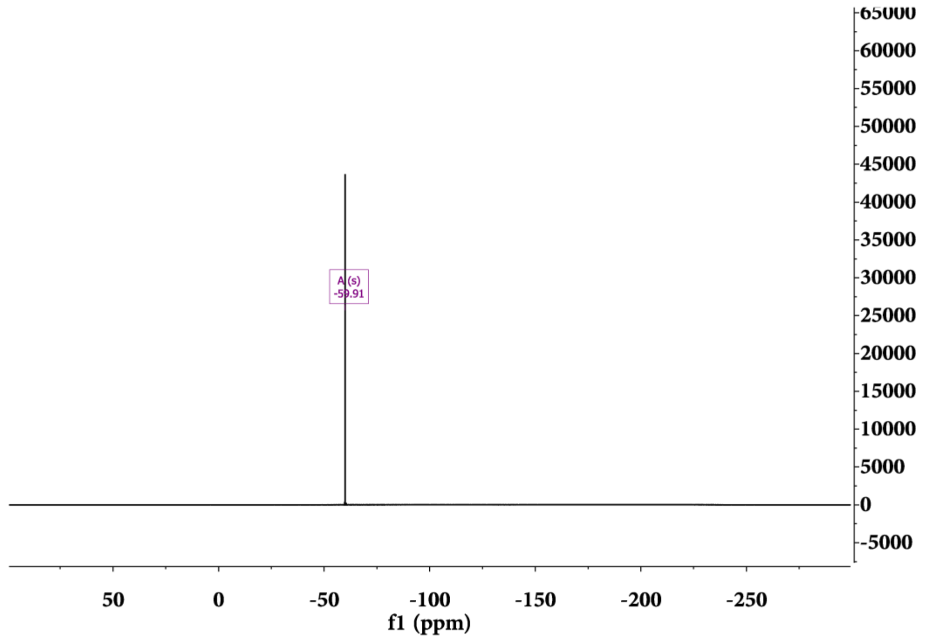

Figure S52. ^{19}F NMR spectrum of **6**.

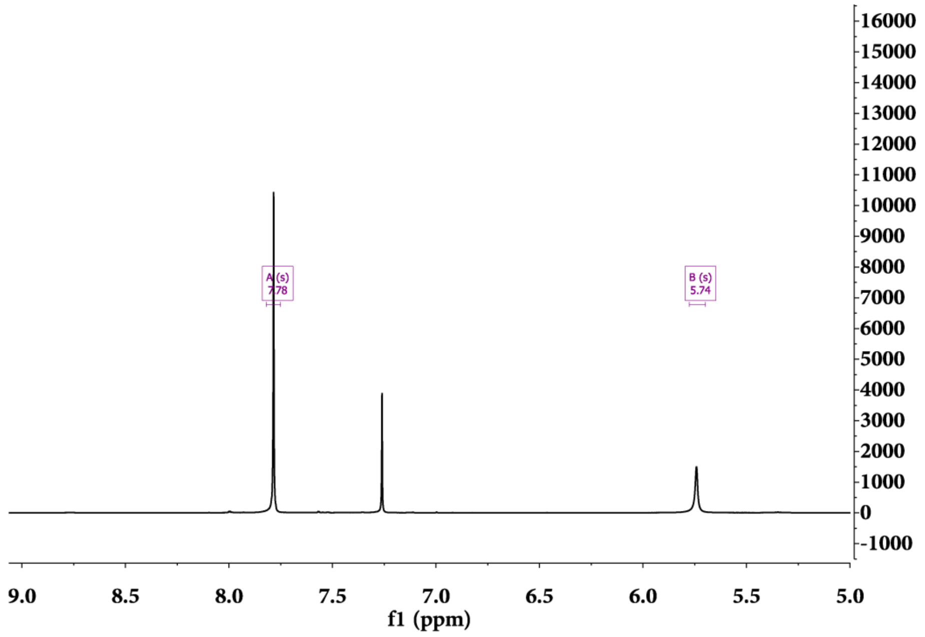

Figure S53. ^{1}H NMR spectrum of **7a**.

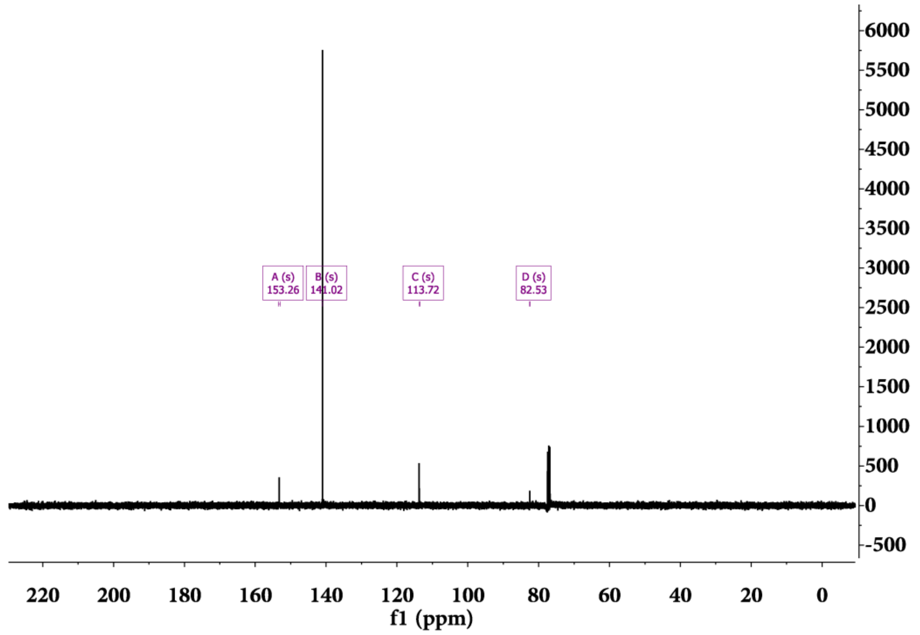

Figure S54. ^{13}C NMR spectrum of **7a**.

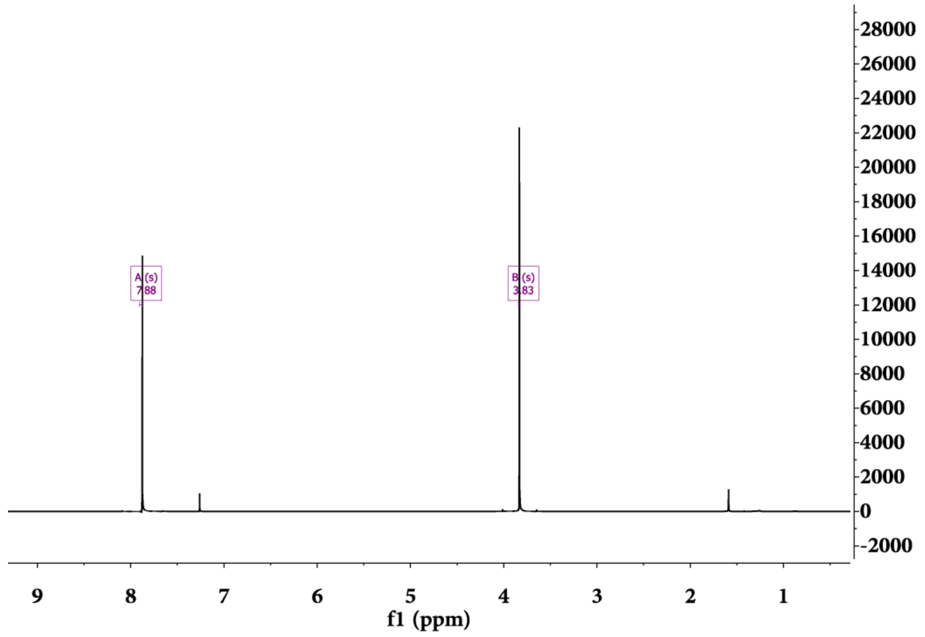

Figure S55. ^{1}H NMR spectrum of **7b**.

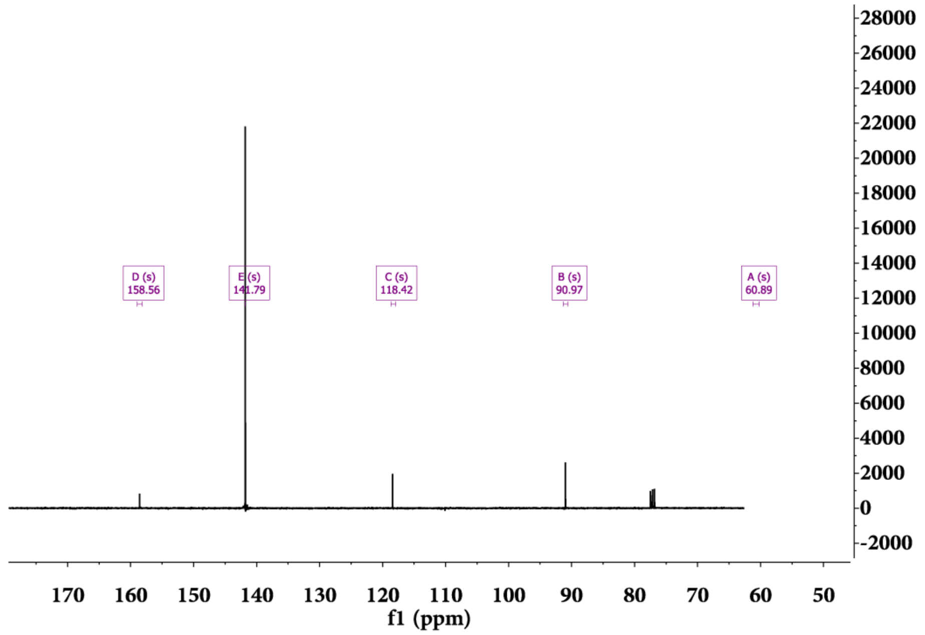

Figure S56. ^{13}C NMR spectrum of **7b**.

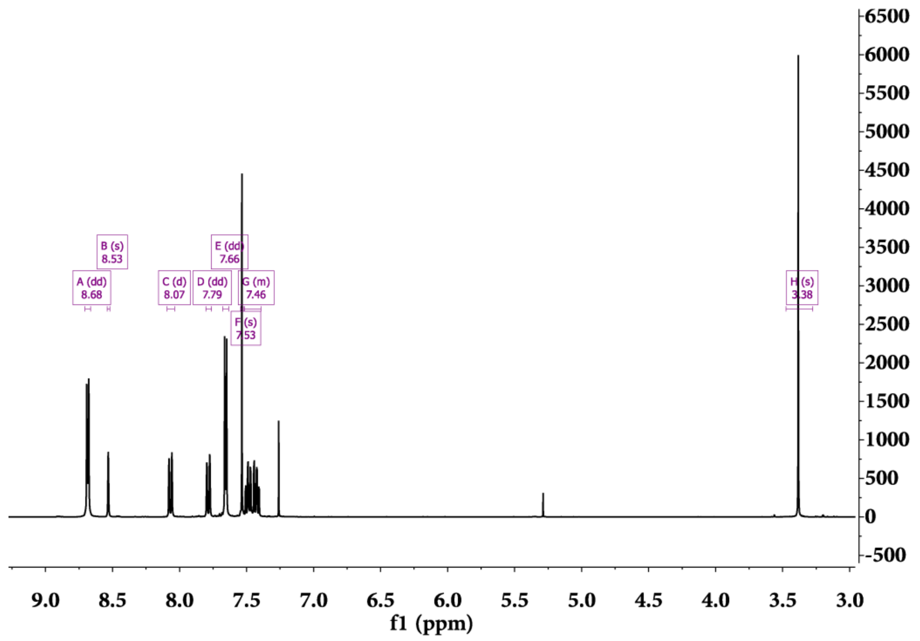

Figure S57. ^{1}H NMR spectrum of **7d**.

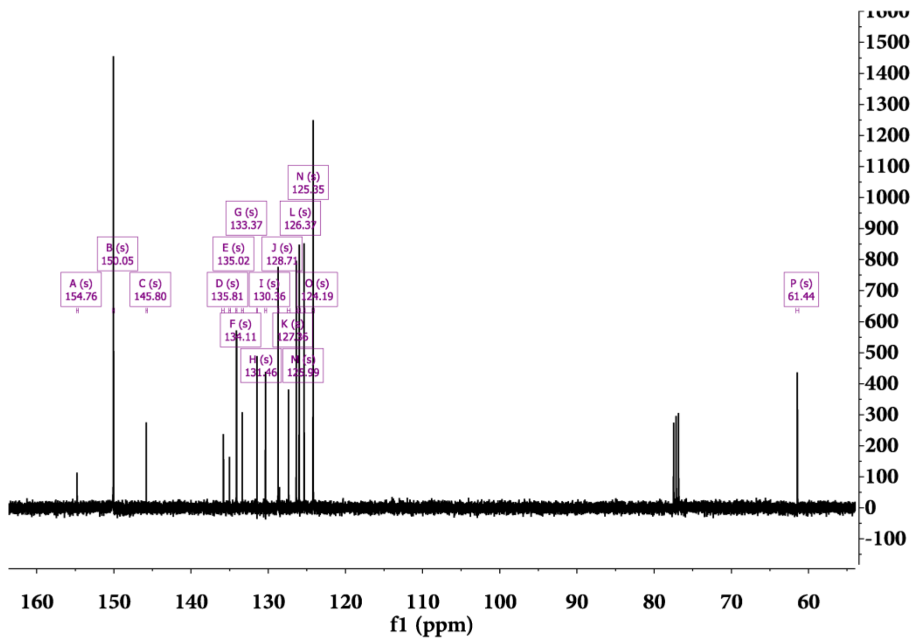

Figure S58. ^{13}C NMR spectrum of **7d**.

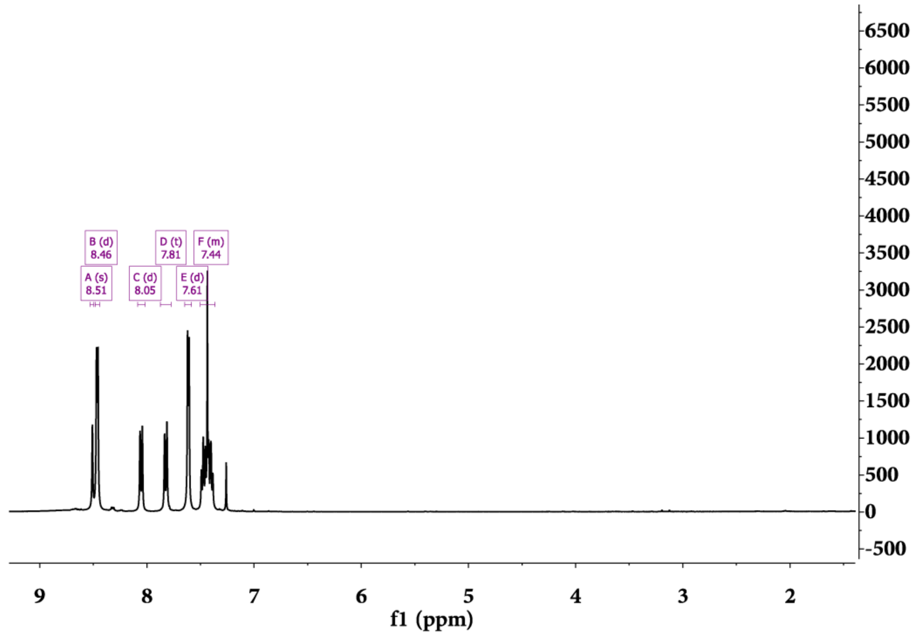

Figure S59. ^{1}H NMR spectrum of **7**.

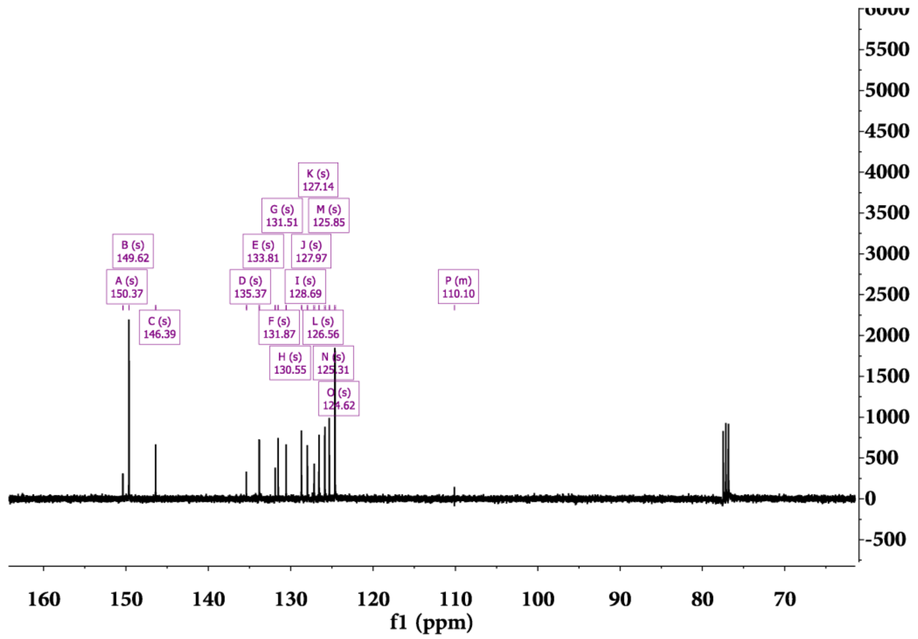

Figure S60. ^{13}C NMR spectrum of **7**.

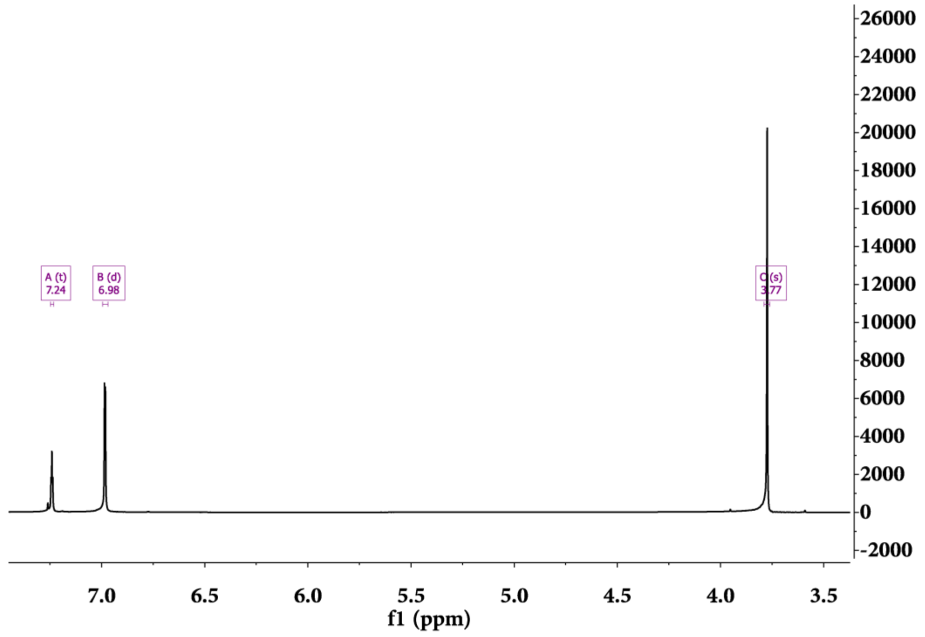

Figure S61. ^{1}H NMR spectrum of **8a**.

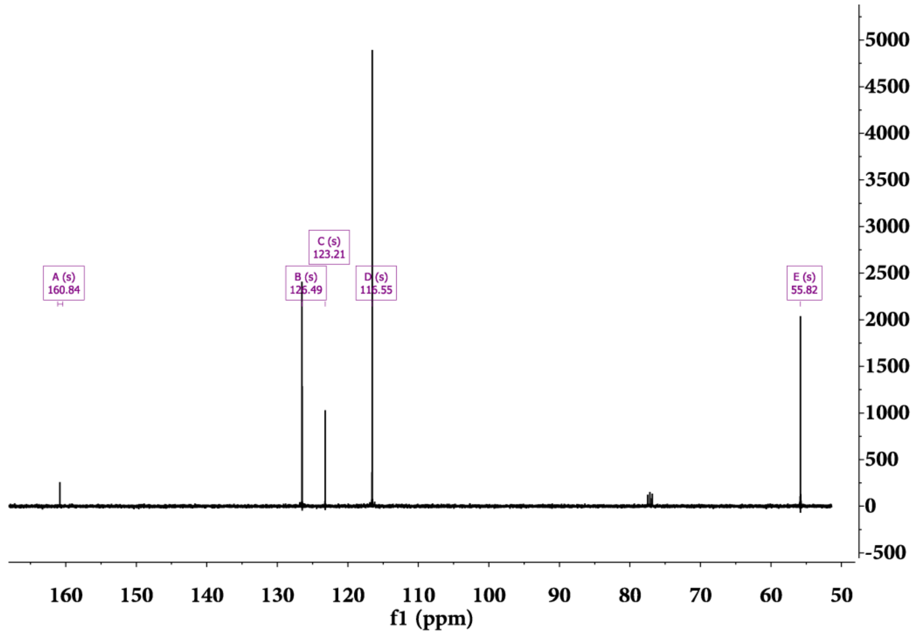

Figure S62. ^{13}C NMR spectrum of **8a**.

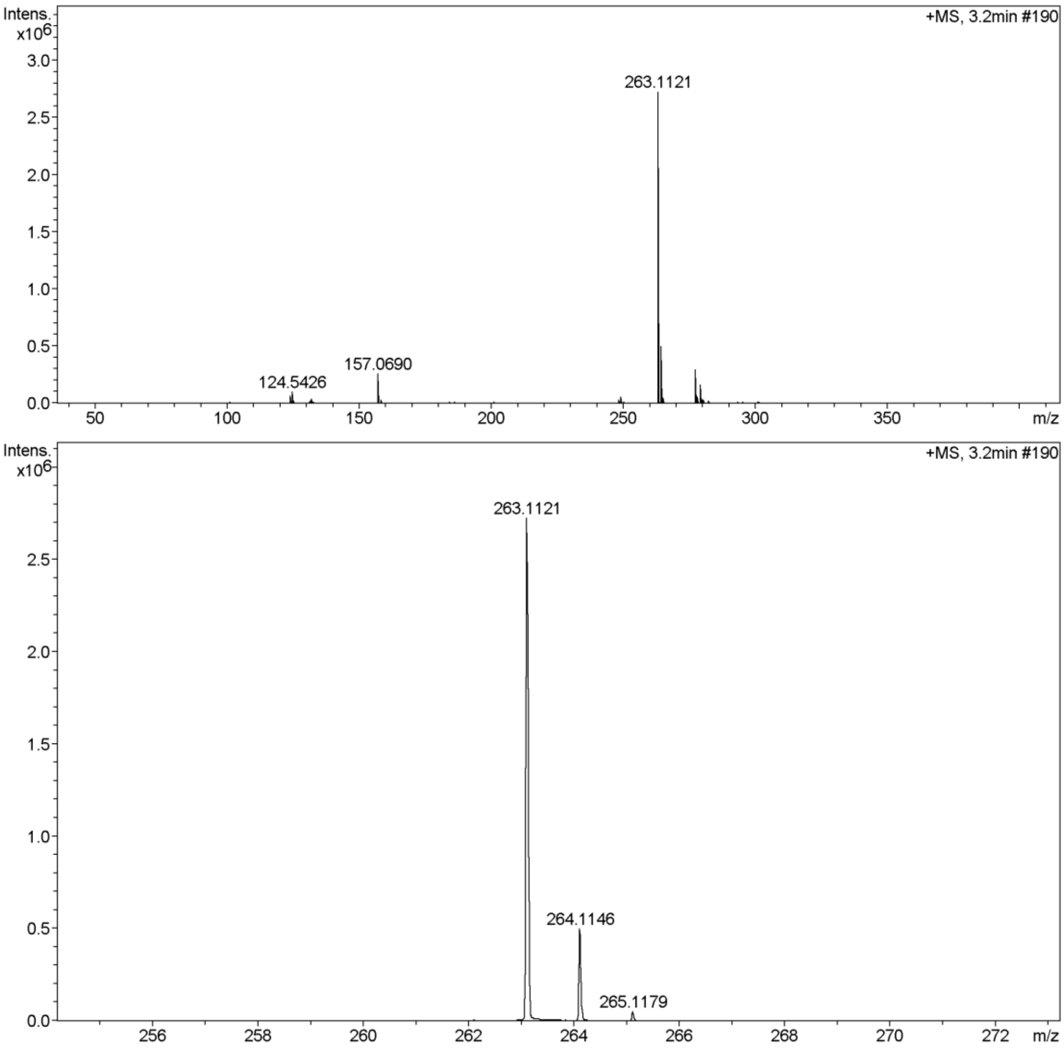

Figure S63. MS spectrum of **3b**.

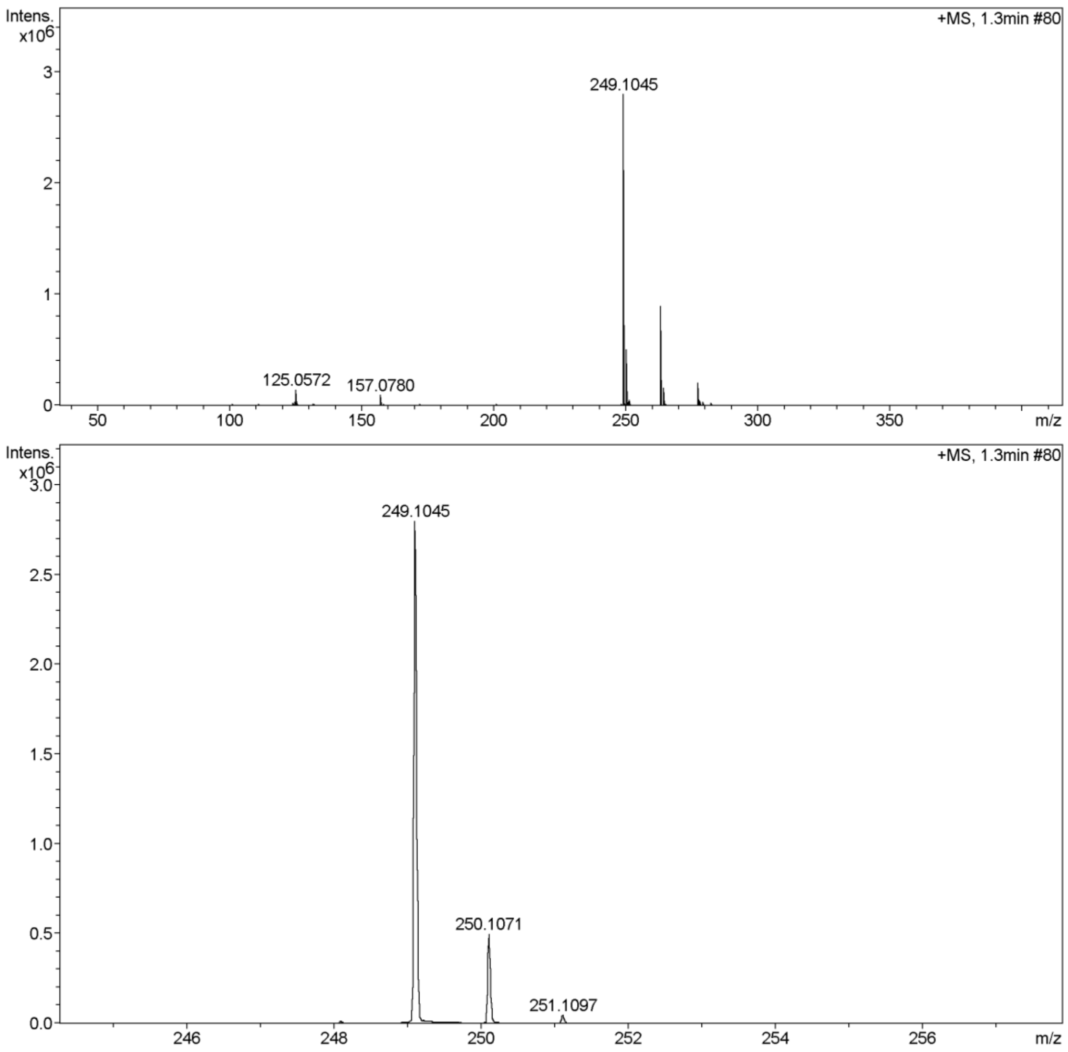

Figure S64. MS spectrum of **3**.

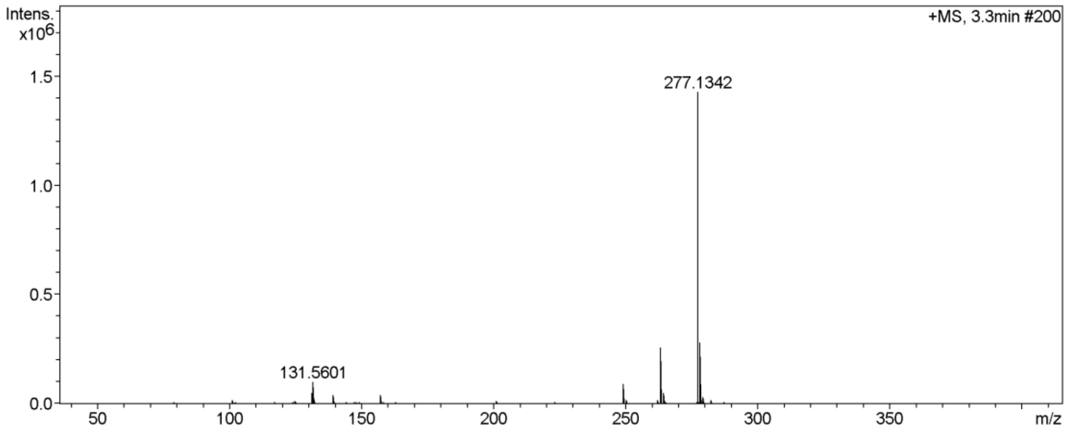

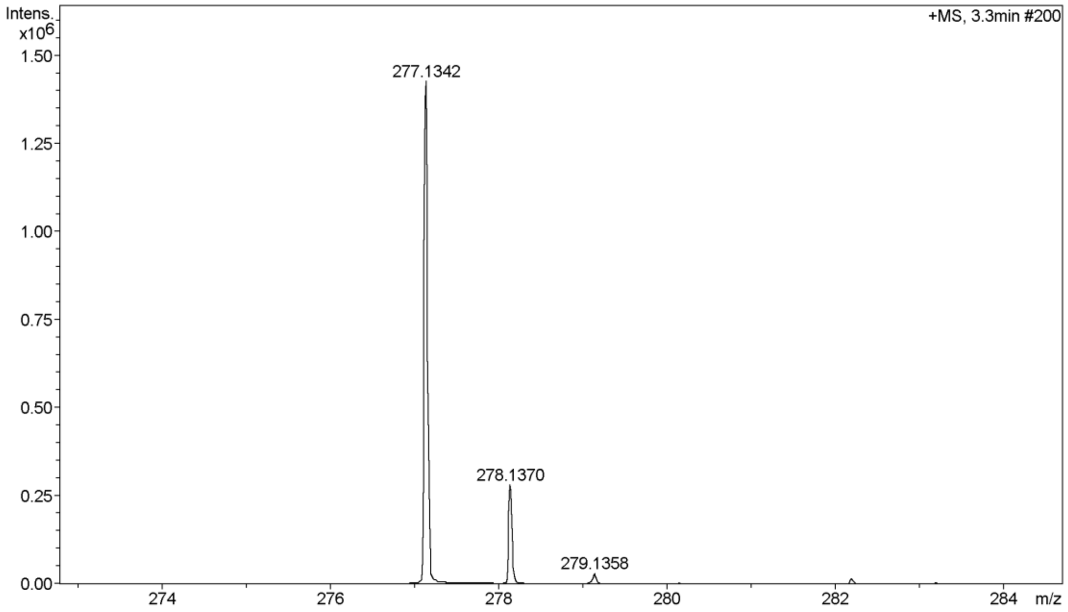

Figure S65. MS spectrum of **4b**.

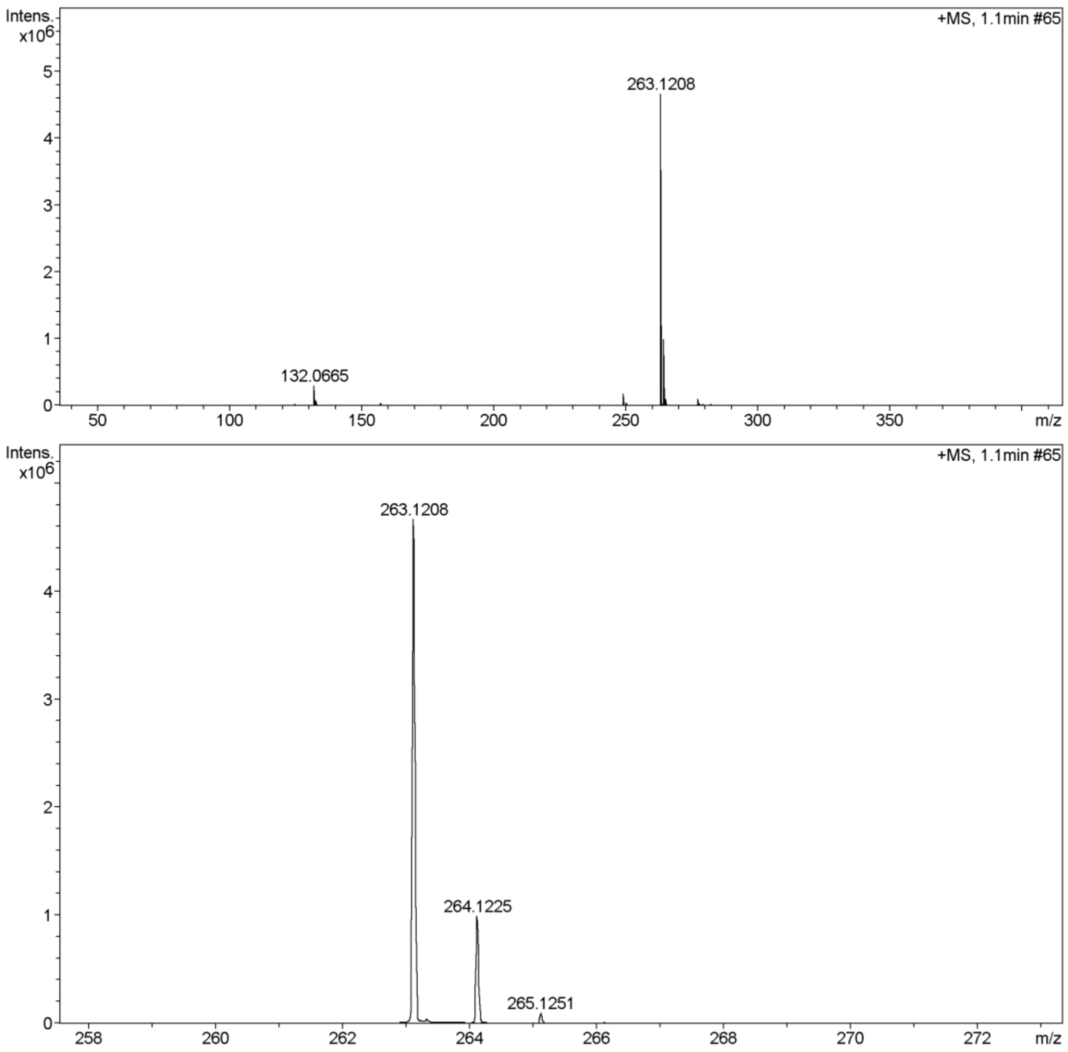

Figure S66. MS spectrum of **4**.

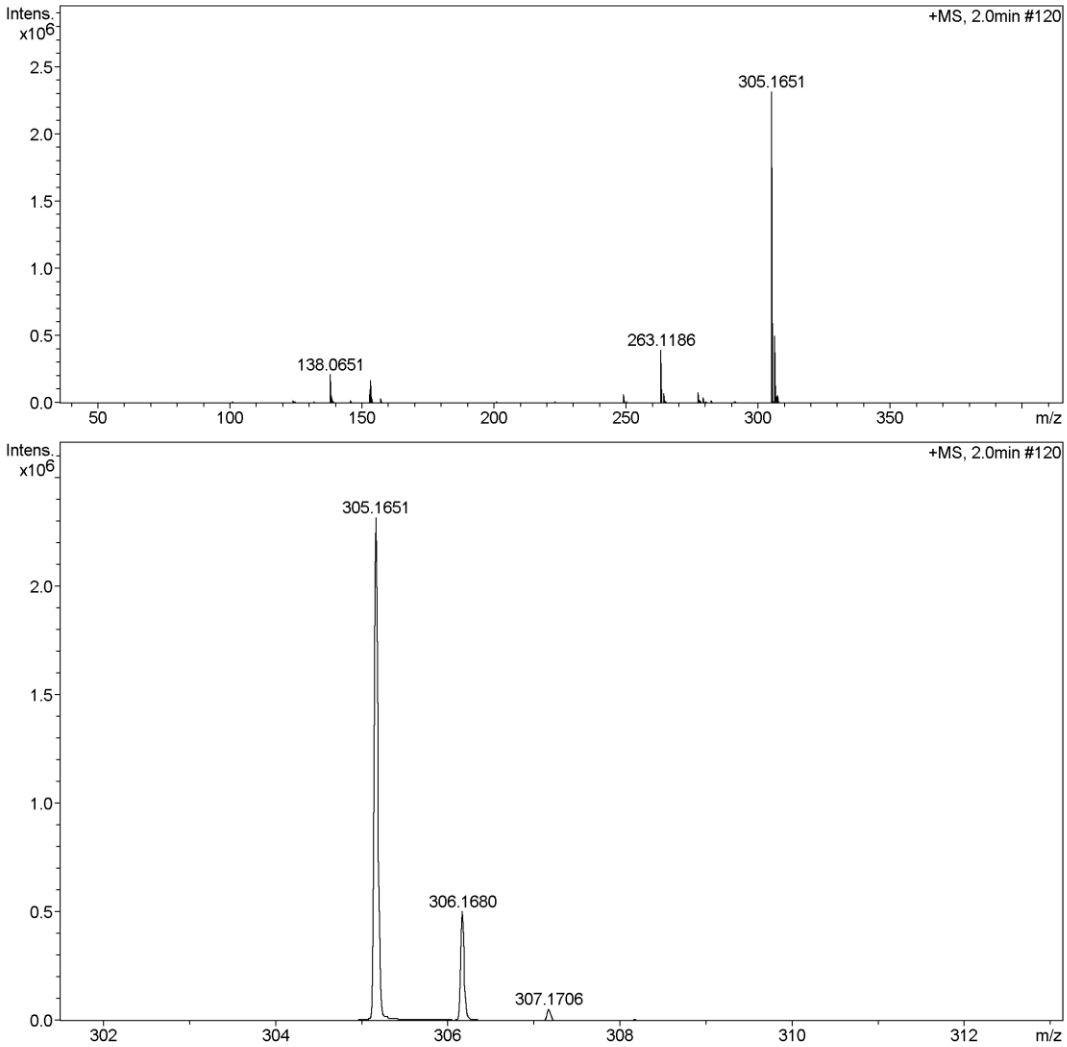

Figure S67. MS spectrum of **5c**.

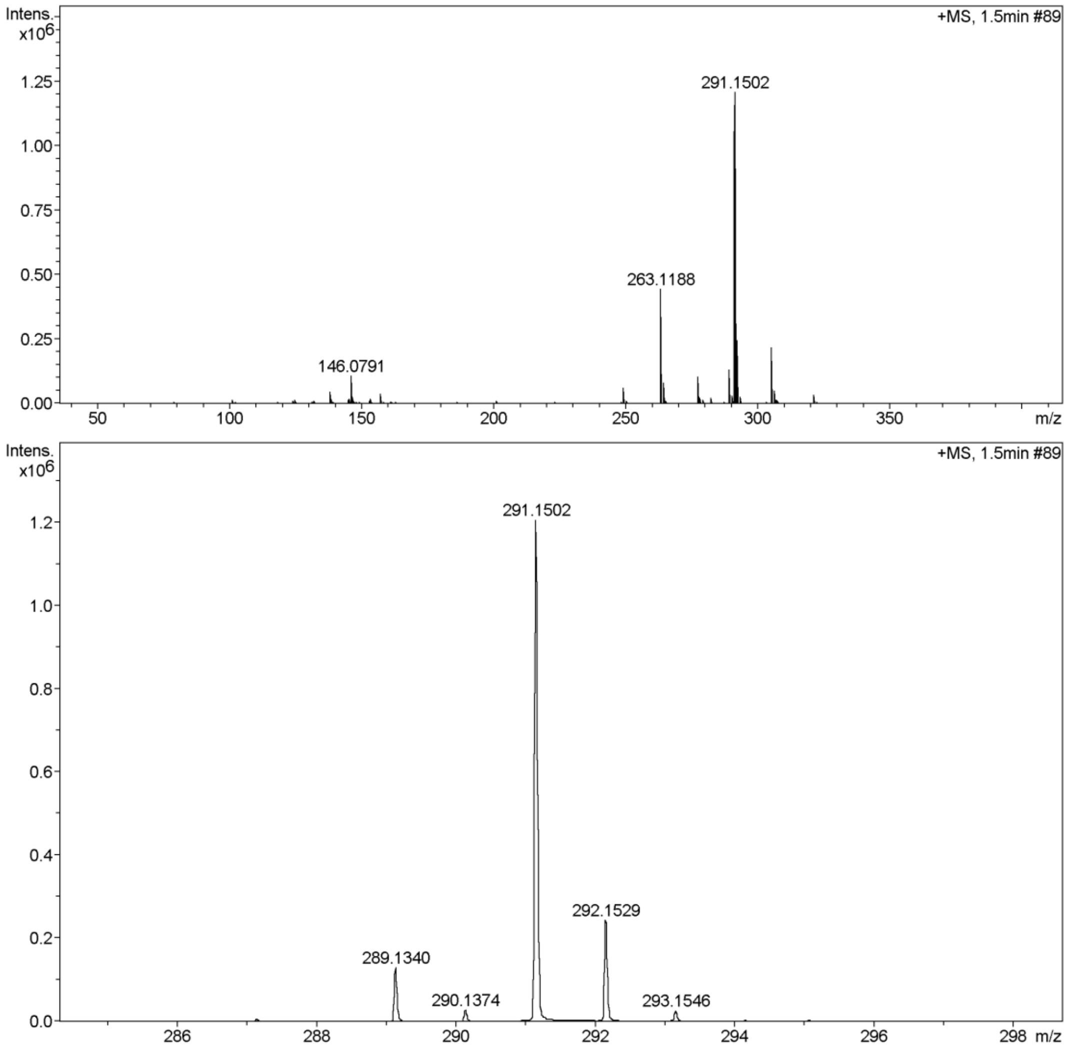

Figure S68. MS spectrum of **5**.

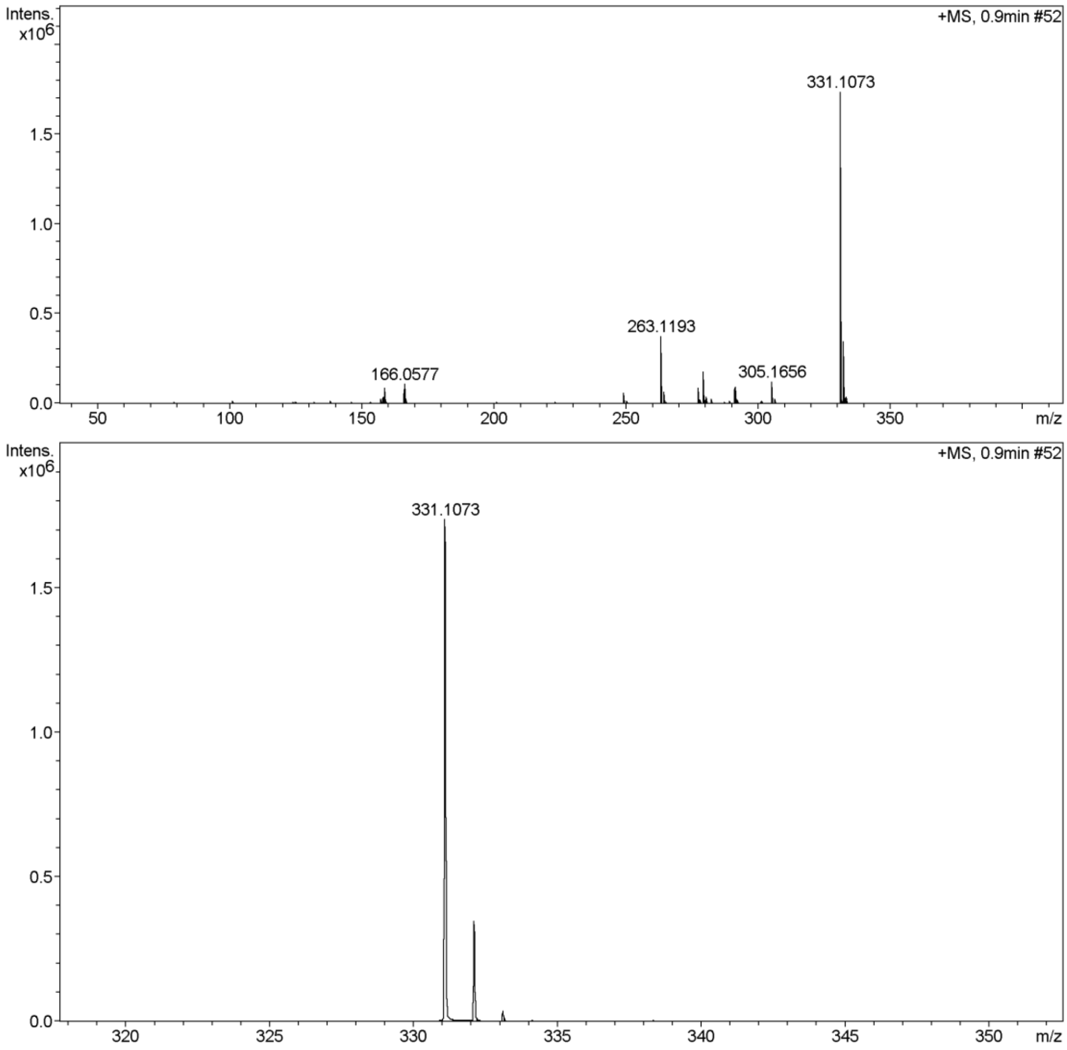

Figure S69. MS spectrum of **6c**.

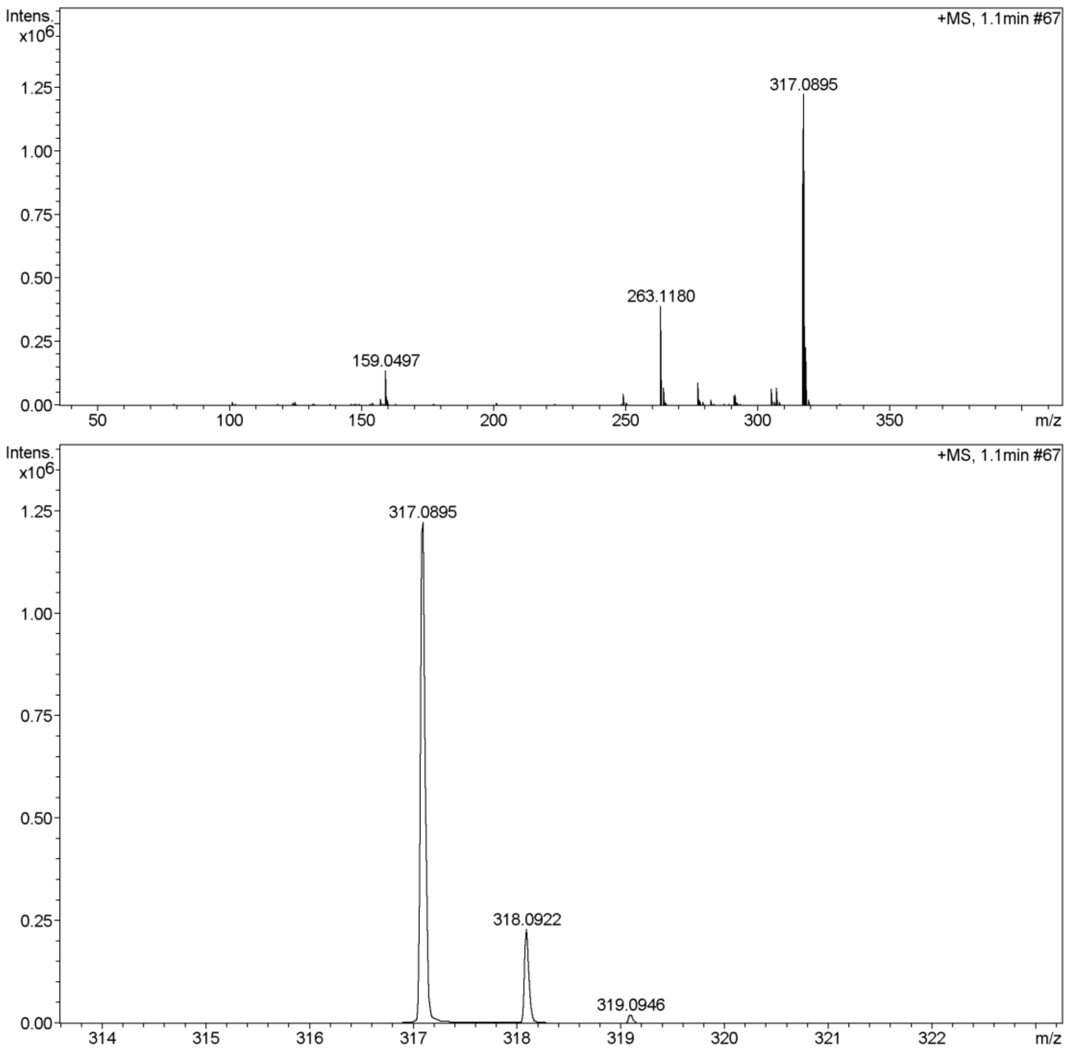

Figure S70. MS spectrum of **6**.

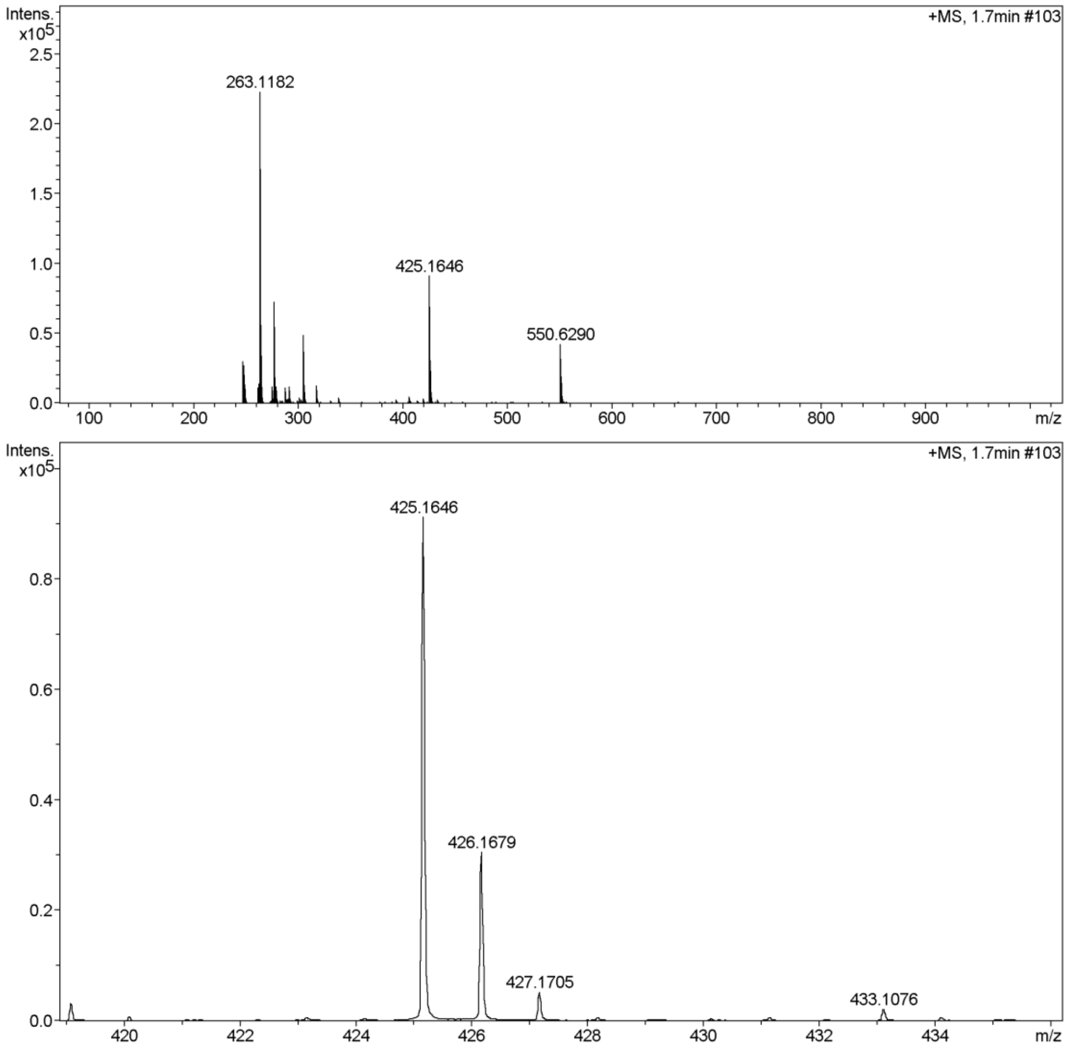

Figure S71. MS spectrum of **7**.

Reference

1. Lee, C., Yang, W. & Parr, R.G. Development of the Colle-Salvetti correlation-energy formula into a functional of the electron density. *Phys Rev B Condens Matter* **37**, 785-789 (1988).
2. Becke, A.D. Density‐functional thermochemistry. III. The role of exact exchange. *The Journal of Chemical Physics* **98**, 5648-5652 (1993).
3. Frisch, M.J., Pople, J.A. & Binkley, J.S. Self‐consistent molecular orbital methods 25. Supplementary functions for Gaussian basis sets. *The Journal of Chemical Physics* **80**, 3265-3269 (1984).
4. Ditchfield, R., Hehre, W.J. & Pople, J.A. Self‐Consistent Molecular‐Orbital Methods. IX. An Extended Gaussian‐Type Basis for Molecular‐Orbital Studies of Organic Molecules. *The Journal of Chemical Physics* **54**, 724-728 (1971).
5. Clark, T., Chandrasekhar, J., Spitznagel, G.W. & Schleyer, P.V. EFFICIENT DIFFUSE FUNCTION-AUGMENTED BASIS SETS FOR ANION CALCULATIONS. III. THE 3-21+G BASIS SET FOR FIRST-ROW ELEMENTS, LI-F. *J. Comput. Chem.* **4**, 294-301 (1983).

6. Grimme, S., Antony, J., Ehrlich, S. & Krieg, H. A consistent and accurate ab initio parametrization of density functional dispersion correction (DFT-D) for the 94 elements H-Pu. *J Chem Phys* **132**, 154104 (2010).
7. Peng, C.Y. & Schlegel, H.B. COMBINING SYNCHRONOUS TRANSIT AND QUASI-NEWTON METHODS TO FIND TRANSITION-STATES. *Israel Journal of Chemistry* **33**, 449-454 (1993).
8. Bauernschmitt, R. & Ahlrichs, R. Treatment of electronic excitations within the adiabatic approximation of time dependent density functional theory. *Chem. Phys. Lett.* **256**, 454-464 (1996).
9. Furche, F. & Ahlrichs, R. Adiabatic time-dependent density functional methods for excited state properties. *J. Chem. Phys.* **117**, 7433-7447 (2002).
10. Yanai, T., Tew, D. P. & Handy, N.C. A new hybrid exchange-correlation functional using the Coulomb-attenuating method (CAM-B3LYP). *Chem. Phys. Lett.* **393**, 51-57 (2004).
11. Gaussian 16, Revision C.01, Frisch, M. J.; Trucks, G. W.; Schlegel, H. B.; Scuseria, G. E.; Robb, M. A.; Cheeseman, J. R.; Scalmani, G.; Barone, V.; Petersson, G. A.; Nakatsuji, H.; Li, X.; Caricato, M.; Marenich, A. V.; Bloino, J.; Janesko, B. G.; Gomperts, R.; Mennucci, B.; Hratchian, H. P.; Ortiz, J. V.; Izmaylov, A. F.; Sonnenberg, J. L.; Williams-Young, D.; Ding, F.; Lipparini, F.; Egidi, F.; Goings, J.; Peng, B.; Petrone, A.; Henderson, T.; Ranasinghe, D.; Zakrzewski, V. G.; Gao, J.; Rega, N.; Zheng, G.; Liang, W.; Hada, M.; Ehara, M.; Toyota, K.; Fukuda, R.; Hasegawa, J.; Ishida, M.; Nakajima, T.; Honda, Y.; Kitao, O.; Nakai, H.; Vreven, T.; Throssell, K.; Montgomery, J. A., Jr.; Peralta, J. E.; Ogliaro, F.; Bearpark, M. J.; Heyd, J. J.; Brothers, E. N.; Kudin, K. N.; Staroverov, V. N.; Keith, T. A.; Kobayashi, R.; Normand, J.; Raghavachari, K.; Rendell, A. P.; Burant, J. C.; Iyengar, S. S.; Tomasi, J.; Cossi, M.; Millam, J. M.; Klene, M.; Adamo, C.; Cammi, R.; Ochterski, J. W.; Martin, R. L.; Morokuma, K.; Farkas, O.; Foresman, J. B.; Fox, D. J. Gaussian, Inc., Wallingford CT, 2016.
