# Supplementary material for: Lighting up solid states using a rubber
Source: Nat Commun. 2021 Feb 10;12:908. doi: 10.1038/s41467-021-21253-w (PMC7876014; doi:10.1038/s41467-021-21253-w)
Supplement: Supplementary file 2 — Description of Additional Supplementary Files [file 41467_2021_21253_MOESM2_ESM.pdf]

Description of Additional Supplementary Files

File Name: Supplementary Movie 1
Description: A rubbing-induced photoluminescence of Compound 3.
